# The Cost-Effectiveness of Two Forms of Case Management Compared to a Control Group for Persons with Dementia and Their Informal Caregivers from a Societal Perspective

**DOI:** 10.1371/journal.pone.0160908

**Published:** 2016-09-21

**Authors:** Janet MacNeil Vroomen, Judith E. Bosmans, Iris Eekhout, Karlijn J. Joling, Lisa D. van Mierlo, Franka J. M. Meiland, Hein P. J. van Hout, Sophia E. de Rooij

**Affiliations:** 1 Department of Internal Medicine, Section of Geriatric Medicine, Academic Medical Center, University of Amsterdam, Amsterdam, the Netherlands; 2 Department of Biostatistics, Yale School of Medicine, New Haven, Connecticut, United States of America; 3 Department of Health Sciences and the EMGO Institute for Health and Care Research, Faculty of Earth and Life Sciences, Vrije Universiteit Amsterdam, Amsterdam, the Netherlands; 4 Department of Epidemiology and Biostatistics, VU University Medical Center, Amsterdam, the Netherlands; 5 Department of General Practice and Elderly Care Medicine, and the EMGO Institute for Health and Care Research, VU University Medical Center, Amsterdam, the Netherlands; 6 Department of Internal Medicine, University Center of Geriatric Medicine, University Medical Center Groningen, Groningen, the Netherlands; Deakin University, AUSTRALIA

## Abstract

**Objectives:**

The objective of this article was to compare the costs and cost-effectiveness of the two most prominent types of case management in the Netherlands (intensive case management and linkage models) against no access to case management (control group) for people with already diagnosed dementia and their informal caregivers.

**Methods:**

The economic evaluation was conducted from a societal perspective embedded within a two year prospective, observational, controlled, cohort study with 521 informal caregivers and community-dwelling persons with dementia. Case management provided within one care organization (intensive case management model, ICMM), case management where care was provided by different care organizations within one region (Linkage model, LM), and a group with no access to case management (control) were compared. The economic evaluation related incremental costs to incremental effects regarding neuropsychiatric symptoms (NPI), psychological health of the informal caregiver (GHQ-12), and quality adjusted life years (QALY) of the person with dementia and informal caregiver.

**Results:**

Inverse-propensity-score-weighted models showed no significant differences in clinical or total cost outcomes between the three groups. Informal care costs were significantly lower in the ICMM group compared to both other groups. Day center costs were significantly lower in the ICMM group compared to the control group. For all outcomes, the probability that the ICMM was cost-effective in comparison with LM and the control group was larger than 0.97 at a threshold ratio of 0 €/incremental unit of effect.

**Conclusion:**

This study provides preliminary evidence that the ICMM is cost-effective compared to the control group and the LM. However, the findings should be interpreted with caution since this study was not a randomized controlled trial.

## Introduction

Dementia is a chronic disorder marked by memory loss, cognitive impairment and behavioral lapses resulting in pronounced consequences for the people with dementia, their families and society. The global prevalence of dementia is estimated at 48.1 million in 2020 and is expected to rise to approximately 90.3 million in 2040 [1]. Worldwide costs of dementia were estimated at US$ 604 billion in 2010 [2, 3]. In high-income countries, informal care (45%) and formal social care (40%) make up the majority of costs, while the contribution of direct medical costs (15%) is smaller [2, 3]. The expected increase in the number of people with dementia will lead to increased long-term care needs, while meeting these needs will become more difficult in the future due to the decreasing workforce. Therefore, the provision and financing of care options for people with dementia and their informal caregivers is an important societal and political issue [1].

Although there is a variety of community and care services available for community-dwelling people with dementia and their informal caregivers, people often lack information regarding available support that may address their care needs [4, 5]. Many experience insufficient alignment, management and continuity of care and support during the disease course [6]. Around 80 percent of caregivers are at risk of becoming overburdened, and this is one of the major risk factors for acute hospitalisation and institutionalization of persons with dementia [7, 8]. Moreover, informal caregivers of persons with dementia are at high risk of developing depressive or anxiety disorders [9, 10].

Case management has been proposed as an instrument to delay institutionalization of the person with dementia and improve the mental health of informal caregivers. Case management is defined as a “collaborative process of assessment, planning, facilitation, care coordination, evaluation, and advocacy for options and services to meet an individual’s and family’s comprehensive health needs through communication and available resources to promote quality and cost-effective outcomes”[11]. Reviews and meta-analyses have yielded inconsistent results regarding case management on person with dementia and caregivers outcomes such as care satisfaction, institutionalization, hospitalization, caregiver burden and depression, and economic outcomes [12–15].

The objective of this article was to compare the costs and cost-effectiveness of the two most prominent types of case management in the Netherlands (intensive case management and linkage models) against no access to case management (control group) for people with already diagnosed dementia and their informal caregivers.

## Methods

The COMPAS (Case management of persons with dementia and their caregivers) project was a prospective, observational, controlled, cohort study. The study protocol was registered with the Dutch Trials Registry (NTR3268) and published elsewhere [16, 17]. This study was a non-randomized control prospective observational study where the intervention was already implemented in the community prior to the start of this study making the registration of this study with the NTR a voluntary action after we started recruitment. The authors confirm that all ongoing and related trials for this intervention are registered. The Medical Ethics Committee of the VU University medical center approved this study in November 2010 and all participants gave written consent. The informal caregiver signed on behalf of the person with dementia if they were unable to understand and reproduce the study goals.

### Participants and setting

The primary informal caregivers (n = 521) and persons with dementia were recruited from various regions of the Netherlands from April 2011 to November 2012. The last follow-up interview was done at the end of June 2014. Recruitment areas included rural areas in the north of Netherlands, semi-rural areas outside of Amsterdam, and urban areas such as Amsterdam. In case management regions, case managers of the participating organizations provided lists of their clients who already receiving care and met the inclusion criteria. In the control group, recruitment took place via outpatient geriatric or neurologic (memory) clinics, Alzheimer centers and general medical practices. Persons with dementia were eligible for this study if they lived at home, had an established/formal diagnosis of dementia, were not terminally-ill, were not anticipated to be admitted to a long term care facility within 6 months, and had an informal caregiver. The informal caregivers were eligible if they were the primary informal caregiver responsible for caring for the person with dementia, had sufficient language proficiency and were not severely ill.

### Care models

The case management models that were evaluated in this study and the content of care in regions without case management were described in detail elsewhere and summarized here [16, 18]. Also, in an additional table the models are compared with each other (S2 Table).

Case managers in the Intensive Case Management Model (ICMM) are appointed at one organization which is specialized in dementia care. They guide and support people with dementia for long periods of time usually starting after diagnosis, and offer medical and psychosocial services from within their own organization [19]. The case manager works in collaboration with an ‘in-home’ multidisciplinary team to tailor care needs of the person with dementia and the informal caregiver [19].

The Linkage Model (LM) is a collaboration between multiple care providers (e.g. home care organizations, general practitioners, social care services) who were already providing health care services in the region and were given the mandate to initiate case management services. After a formal diagnosis, persons with dementia are assigned to a case manager who provides educational, emotional and practical support such as advice on disease-related issues, and gives recommendations on the availability of supportive health and social services until time of nursing home admission or death of the persons with dementia. In general, informal caregivers are involved in this process whenever possible. Expert advice can be sought through multidisciplinary meetings held regularly with experts from the various collaborating organizations.

The control group was recruited in areas without access to a case manager [20].^.^In these regions, no central coordination of dementia care is provided by a dedicated, trained and educated health care professional. Care is usually initiated by the person with dementia, his /her informal caregiver or an involved health care provider. In some cases, care may be monitored by a registered nurse working in the general practice in addition to the general practitioner. Access to home or respite care did not differ across regions.

### Data collection

Data were collected through interviews and questionnaires administered by trained interviewers at baseline and every six months for 24 months. The informal caregiver filled in cost diaries that were collected at each interview.

### Outcome measures

The primary outcome in the person with dementia was the presence of neuropsychiatric symptoms as measured with the Neuropsychiatric Inventory (NPI) which assesses twelve neuropsychiatric domains in persons with dementia [21]. The NPI was rated by a caregiver familiar with the person with dementia’s behaviour [21]. Presence, frequency, severity and the symptom specific caregiver distress in the previous month are assessed [21]. Calculation of the total score was the sum of the 12 domain scores which ranges from 0–144 points with higher scores indicating more problems [21].

The primary outcome in the informal caregiver was psychological or mental health as measured by the general health questionnaire (GHQ-12) [22]. The score ranges from 0–12 with higher scores indicating more severe psychological health problems.

Secondary outcomes included Quality-Adjusted Life-Years (QALYs) based on the EuroQol (EQ-5D-3L)[23]. EQ-5D-3L data for the person with dementia were collected by interviewing the informal caregiver [24]. The questions were specifically framed so the informal caregiver would answer taking in mind how their loved one would answer each question and not their personal opinion. Informal caregivers also filled out the EQ-5D-3L for themselves so that their own health related quality of life could be evaluated. The EQ-5D-3L includes 5 dimensions: mobility, self-care, usual activities, pain/discomfort, and anxiety/depression. The respondent answers each of the EQ-5D-3L’s 5 dimensions with 1 of 3 possible responses: “no problems,” “some problems,” or “severe problems.” The set of 5 responses defines a health state [23]. The 243 (3^5^) possible health states are weighted using a valuation set from a sample of the Dutch general population known as the Dutch EQ-5D-3L tariff resulting in a utility score [25]. This utility reflects the relative desirability of a particular health state and is measured on a scale in which 0 is anchored to death and 1 to perfect health. We calculated QALYs by multiplying the utility of each health state by the time in between two measurements and summing the results over the 24 month treatment period. QALYs were calculated using the area under the curve approach. Transitions between health states were linearly interpolated.

Subsequently, combined QALY scores for the pairs were calculated by summing the QALYs for the person with dementia and the caregiver. The rationale was that case management can influence outcomes at the level of both the informal caregiver and the person with dementia, which we tried to account for by calculating a combined QALY score.

### Costs

Resource utilization and associated costs were measured from a societal perspective. Cost diaries were used to collect data on use of care and support by persons with dementia and the informal caregiver? to estimate costs from a societal perspective. Direct healthcare costs included formal care such as general practitioner visits and medication use. Direct non-healthcare costs consisted of time spent on care by informal carers. We limited the maximum number of informal care hours per day to 18 hours. Indirect non-healthcare costs included days absent from paid work or unable to do daily activities such as housekeeping or voluntary work because of caregiver responsibilities. S1 Table lists the cost categories and prices used in the economic evaluation. All prices were adjusted for the year 2010 using consumer price index figures [26]. Healthcare utilization, and absenteeism from paid and unpaid work was valued using Dutch standard costs [27]. Medication use was valued using prices from the Royal Dutch Society for Pharmacy [28].

To estimate the costs of case management, 73 case managers (92% of all case managers that were included in this study) were interviewed about their caseloads and the number of hours they worked per week. Based on this information we calculated the time spent per client including travel time, and valued this using the median hourly wage for a case manager.

Costs and effects in the second year were discounted at 4% and 1.5% respectively based on Dutch guidelines for economic evaluations [29].

### Statistical analysis

As this was a non-randomized controlled study, advanced statistical methods were needed to control for any baseline imbalances between the different treatment groups [30]. Therefore, propensity scores were calculated using generalized boosted methods for multiple treatments using the ‘twang’ package in R [31]. Balance and overlap of propensity score distributions in the three groups were assessed. The ‘twang’ package provides propensity scores based weights for the estimation of the average treatment effects (ATE) for more than two treatment groups. All covariates that significantly differed among groups at baseline or that were associated with the baseline NPI total score were included in the calculation of propensity score and weights. We checked the weighted averages of the covariates to confirm that there were no longer any baseline differences between the groups. A separate propensity score and weight was created for secondary outcomes that included variables that significantly differed at baseline. The propensity score based weights were then exported to Stata to be used as sampling weights in the analysis [32].

Several studies showed that using complete case analysis is likely to result in bias in economic evaluations [33–35]. Therefore, multiple imputation by chained equations (MICE) using predictive mean matching (PMM) was used to impute missing values for cost and effect data [33]. Variables associated with missingness in the current study included: education, living situation, sex of the person with dementia, MMSE, care needs, mastery of the informal caregiver, relationship to the person of dementia, loneliness of informal caregiver, age of the informal caregiver, baseline NPI, multimorbidity, Katz and the carerQol. Because we felt that we could properly explain the missing data mechanism, we assumed that data was missing at random (MAR) which is a prerequisite for using multiple imputation (MI). MI was done taking into account recommendations presented in various recent papers [33, 34, 36].

We created 100 imputed datasets. We included the propensity weight as one of the predictor variables in the imputation model [30]. The analysis results from the imputed datasets were pooled together using Rubin’s rules [37]. Individual sub costs per category were imputed instead of total costs to maximize the accuracy of the imputation.

We used a generalized linear regression model with a gamma distribution and an identity link to estimate mean differences in total costs while accounting for the right skew of the cost data. The incremental effect in quality adjusted life years (QALYs) was estimated using a using a generalized linear regression model adjusted for baseline utility scores with a Gaussian distribution and an identity link [38]. A generalized estimating equation (GEE) model was used to estimate NPI and GHQ-12 scores to allow for repeated measurements. An exchangeable correlation structure was used with robust standard errors. The estimated changes in mean NPI, GHQ and sub costs that are reported are for the two year follow-up period.

We estimated the correlation between the incremental total costs and the different outcome measures in the imputed datasets. In the multiple imputation procedure, the covariance between total costs and effect outcomes measures were calculated based on the Fisher z transformation and were then pooled using Rubin’s rules [36, 39].

Incremental Cost-Effectiveness Ratios (ICERs) were calculated using the pooled cost and effect estimates. The ICER is calculated as $\frac{{\hat{\Delta }}_{c}}{{\hat{\Delta }}_{e}}$ where ${\hat{\Delta }}_{c}$ is the difference in total costs between the two intervention groups and ${\hat{\Delta }}_{e}$ is the difference in QALYs between the two intervention groups. To show uncertainty around the ICER a cost-effectiveness plane was estimated using bootstrapping with 1000 replications using the syntax developed by Faria et al [34].

Incremental net benefit (INB) estimates were calculated using the following formula: $\hat{b}(\lambda )={\hat{\Delta }}_{e}\lambda -{\hat{\Delta }}_{c}$ [40, 41] where ${\hat{\Delta }}_{e}$ is the difference in outcome (for example QALY) between the two intervention groups, *λ* is the willingness to pay, and ${\hat{\Delta }}_{c}$ is the difference in costs. The variance of INB was calculated using: $V[\hat{b}(\lambda )]=\hat{V}({\hat{\Delta }}_{e})\lambda^{2}+\hat{V}({\hat{\Delta }}_{c})-2\hat{C}({\hat{\Delta }}_{e},{\hat{\Delta }}_{c})\lambda$ where $\hat{C}$ is the covariance between the differences in total costs and QALYs [40, 41]. The probability of cost-effectiveness was calculated by estimating the probability that INB was positive using a normal-based approach. Using the pooled NMB and the pooled standard error of NMB, the probability that NMB is larger than zero was estimated for a range of willingness-to-pay values. These probabilities were then used to estimate cost-effectiveness acceptability curves (CEACs) which is a plot of the probability that the intervention is cost-effective in comparison with its comparator (y-axis) as a function of the money society might be willing to pay for one additional unit of outcome (threshold ratio (*λ*), x-axis). In this way, CEACs quantify the uncertainty due to sampling and measurement errors while taking into account that *λ* is generally unknown [40, 41]. The pooled coefficients and variance parameters from the regression models were used to estimate the CEACs. CEACs were estimated for three comparisons: ICMM versus control, LM versus control, and ICMM versus LM. The Netherlands has a “rule of thumb” value for *λ* of around 30,000 €/QALY [29]. Therefore, probabilities of cost-effectiveness for all outcomes at this value for *λ* were presented as part of the cost-effectiveness outcomes.

## Results

**Fig 1:**
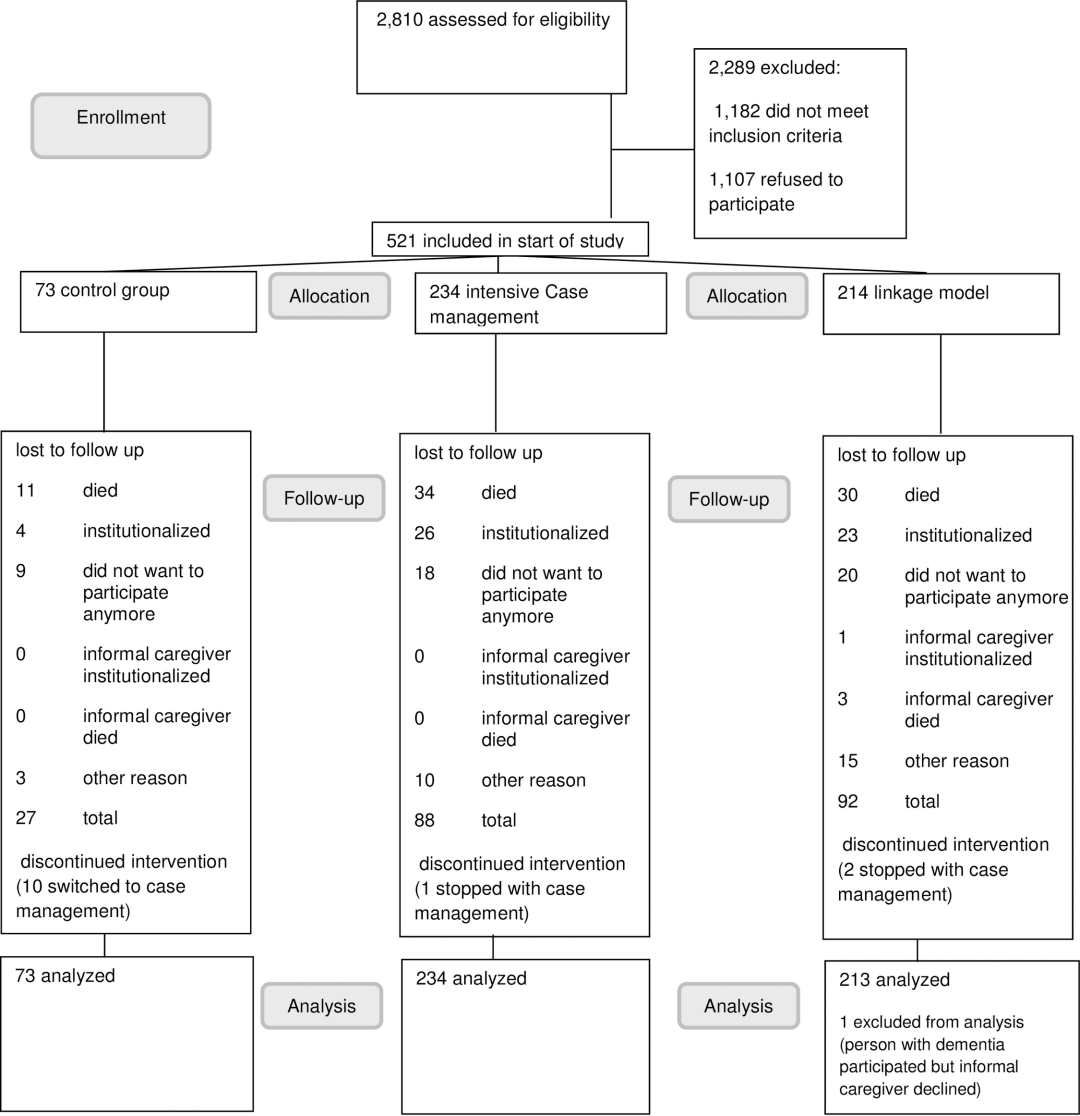
Consort diagram. Recruitment overview.

Fig 1 shows the flow of participants in the study. Of the 2,810 caregiver and person with dementia pairs assessed for eligibility, 1,628 met all inclusion criteria and were sent recruitment letters. Five hundred and twenty-one of these individuals agreed to participate (32%) and 1,107 (68%) refused to participate. We had information on the gender of the person with dementia, the gender of the caregiver and the relationship of the informal caregiver to the person with dementia for 1,172 (72%) people that were approached. The only difference we found was that informal caregivers who were the partner of the person with dementia were more willing to participate than those who had another type of relationship (χ^2^ = 11 df = 1, p<0.001).

Participant characteristics and differences between groups are shown in Table 1. Of the 521 participants included, 234 (45%) participated in the ICMM group, 73 (14%) in the control group and 214 (41%) in the LM group. The average age of informal caregivers was 65 years (range 22–91 years), and the average age of the person with dementia was 80 years (range 54–97 years). Sixty-seven percent of the informal caregivers and 55% of the persons with dementia were female. The number of participants that dropped out was 219 (42%) after 24 months. The rate of drop out did not differ per group and the main reason for drop out was death of the person with dementia (n = 82/219, 37%). There was no significant difference in the proportion of deaths between the three groups (n = 39 deaths for ICMM n = 32 for LM and n = 11 for control group). Persons with dementia who died during the study were more likely to be older (-3.1 years, 95% CI: -5.0, -1.30), had more comorbid disorders (χ2 = 4 df = 1, p = 0.04), poorer Katz scores (-2.1, 95% CI: -2.9, -1.2), and lower MMSE scores at baseline (3.2, 95% CI: 1.49, 5.0).

**Table 1:** Baseline table of characteristics of care models, persons with dementia and informal caregivers.

	Intensive Case Management	Linkage model	Control	Total group	P- value
Person with Dementia	N = 234	N = 214	N = 73	N = 521	
Age, mean (SD) ^a^	79.9	81.0	75.9	79.8	< 0.001
	(7.7)	(7.5)	(8.7)	(7.9)	
Female gender, n (%) ^b^	122	134	32	288	0.009
	(52.4)	(62.6)	(43.8)	(55.3)	
Married or in a relationship, n (%) ^b^	128	98	51	277	0.003
	(56.4)	(47.8)	(70.8)	(55.0)	
Living situation					0.065
Living alone, n (%) ^b^	92	95	19	206	
	(40.5)	(46.3)	(26.8)	(41.0)	
Living with another person	130	105	49	284	
	(57.3)	(51.2)	(69.0)	(56.5)	
Living in an elderly home	5	5	3	13	
	(2.0)	(2.4)	(4.2)	(2.6)	
Born in the Netherlands, n (%) ^b^	209	178	64	451	0.204
	(92.1)	(86.8)	(88.9)	(89.5)	
Education, n(%) ^b^					0.011
Elementary/lower	93	99	21	213	
	(41.9)	(49.5)	(29.6)	(43.2)	
Secondary	111	81	37	229	
	(50.0)	(40.5)	(52.1)	(46.5)	
Higher/University	18	20	13	51	
	(8.1)	(10.0)	(18.3)	(10.3)	
MMSE, mean (0–30) (SD) ^a^	19.6	18.7	20.4	19.3	0.150
	(5.5)	(6.4)	(4.8)	(5.8)	
Time since symptoms in years, median (IQR) ^c^	3.5	3.8	4.0	3.7	0.641
	(2.0–5.0)	(2.0–5.3)	(2.7–5.5)	(2.0–5.2)	
Time since diagnosis in years, median (IQR) ^c^	2.4	2.1	2.0	2.3	0.267
	(1.4–3.7)	(1.3–3.3)	(1.3–3.0)	(1.3–3.5)	
Time in Case Management in years, median (IQR) ^d^	2.1	1.7	NA	1.8	< 0.001
	(1.3–3.1)	(0.42–2.5)		(1.1–2.8)	
Multi-morbidity (more than two diseases),n (%) ^b^	203	172	55	430	0.032
	(88.7)	(83.5)	(76.4)	(84.8)	
Utility from the EQ-5D-Proxy (0–1) (SD) ^e^	0.7	0.7	0.7	0.7	0.299
	(0.2)	(0.3)	(0.2)	(0.2)	
EQ-5D utility from persons with dementia (0–1) (SD)	0.8	0.8	0.8	0.8	0.415
	(0.2)	(0.2)	(0.2)	(0.2)	
QOL-AD proxy (13–52) (SD)	31.9	31.7	32.7	32.0	0.483
	(5.1)	(5.2)	(5.0)	(5.1)	
Informal Caregiver	Intensive Case Management	Linkage model	Control	Total group	P- value
Age (SD) ^a^	64.5	64.4	65.8	64.6	0.687
	(12.8)	(12.4)	(11.7)	(12.5)	
Female gender, n (%) ^b^	163	136	49	348	0.390
	(70.0)	(63.6)	(67.1)	(66.8)	
Spouse of the person with dementia, n (%) ^b^	122	94	50	266	0.002
	(53.3)	(45.6)	(69.4)	(52.5)	
Living together with person with dementia, n (%)^b^	127	100	50	277	0.007
	(55.5)	(48.8)	(70.4)	(54.9)	
Multi-morbidity (one or more diseases), n (%) ^b^	149	119	50	318	0.129
	(65.1)	(57.8)	(69.4)	(62.7)	
Mean number of conditions	3.8	3.8	3.0	3.7	0.330
	(2.2)	(2.2)	(1.9)	(2.1)	
Education, n (%) ^b^					0.370
Elementary/lower	36	31	10	77	
	(16.0)	(15.3)	(13.9)	(15.4)	
Secondary	139.0	127.0	38.0	304.0	
	(61.8)	(62.6)	(52.8)	(60.8)	
Higher/University	50	45	24	119	
	(22.2)	(22.2)	(33.3)	(23.8)	
EQ-5D utility (0–1) (SD)	0.8	0.9	0.9	0.8	0.261
	(0.2)	(0.2)	(0.2)	(0.2)	

^a^ One-way-Anova

^b^ Chi-square test

^c^ Kruskall-Wallis test

^d^ Mann-Whitney test

^e^The underlined scores indicates the more positive outcomes.

### Clinical outcomes

Table 2 presents the results of the two-year clinical outcomes. Differences in the primary and secondary outcomes between groups were small and not statistically significant.

**Table 2:** Clinical outcomes raw mean scores and standard errors after 24 months.

	ICMM		LM		Control				
	(n = 234)		(n = 214)		(n = 73)				
primary outcomes	Crude^a^		Crude^a^		Crude^a^		ICMM vs control	LM vs control	ICMM vs LM
	mean	SE	mean	SE	mean	SE	Adjusted differences	Adjusted differences	Adjusted differences
							(95% CI)	(95% CI)	(95% CI)
Person with dementia QALY	1.25	0.04	1.18	0.04	1.27	0.06	-0.004	-0.01	0.01
							(-0.14; 0.13)	(-0.14; 0.12)	(-0.09; 0.10)
Person with dementia utility scores									
Baseline	0.74	0.01	0.7	0.02	0.74	0.03			
6 months	0.68	0.02	0.65	0.02	0.7	0.04			
12 months	0.64	0.02	0.58	0.03	0.68	0.04			
18 months	0.58	0.03	0.54	0.03	0.57	0.05			
24 months	0.52	0.03	0.5	0.03	0.5	0.05			
Informal caregiver+ Person with dementia QALY combined score	2.9	0.04	2.9	0.05	3.0	0.07	0.0004	-0.03	0.03
							(-0.16; 0.16)	(-0.19; 0.12)	(-0.08; 0.15)
Informal caregiver utility scores									
Baseline	0.83	0.01	0.85	0.01	0.86	0.02			
6 months	0.82	0.01	0.84	0.01	0.83	0.02			
12 months	0.82	0.01	0.82	0.02	0.84	0.02			
18 months	0.82	0.01	0.81	0.02	0.84	0.02			
24 months	0.83	0.01	0.82	0.02	0.83	0.03			
NPI							-1.54	0.33	-1.86
							(-7.15; 4.07)	(-5.37; 6.02)	(-5.4; 1.67)
Baseline	16.8	1	19.9	1.1	15	1.6			
6 months	21.7	1.6	22.1	1.7	22.7	2.7			
12 months	19.7	1.4	21.5	1.5	19.8	2.2			
18 months	22.4	1.6	25.2	1.8	25.1	3.2			
24 months	23.2	1.7	26.7	2.1	26.7	3.4			
GHQ-12							-0.11	-0.09	-0.02
							(-0.95; 0.72)	(-0.94; 0.76)	(-0.59; 0.55)
Baseline	3.2	0.2	3.3	0.2	3	0.4			
6 months	3.3	0.2	2.9	0.2	4.1	0.4			
12 months	3.5	0.2	3.4	0.3	3.9	0.4			
18 months	3.5	0.3	3.6	0.3	4.4	0.5			
24 months	3.5	0.3	3.6	0.3	4	0.5			

QALY is Quality-adjusted-life-year, ICER is Incremental Cost Effectiveness Ratio, NPI is Neuropsychiatric Inventory,GHQ-12 is General health questionnaire-12,CI is Confidence Interval

^a^ Crude group means, no adjustment using inverse-propensity-score-weights

### Costs

Table 3 presents the adjusted mean costs and the adjusted differences in costs over 2 years between the groups. We found that total costs were lowest for the ICMM followed by LM and then the control group. However, there were no significant differences in total societal costs between groups. Informal care costs in the ICMM and were significantly lower than in the control (95% CI -17,080;-1,674) and the LM group (95% CI -11,473;-1,660). Day center costs were significantly lower in the ICMM group than the control group (95% CI -8,693;-50).

**Table 3:** Adjusted sub costs and costs along with differences between groups over 24 months (in Euros).

	ICMM	LM	Control			
	n = 234	n = 214	n = 73			
cost category	Mean	Mean	Mean	ICMM vs Control^a^	LM vs Control^a^	ICMM vs LM^a^
	Cost^a^	Cost^a^	Cost^a^			
	(SE)	(SE)	(SE)	(95% CI)	(95% CI)	(95% CI)
General practice	1,279	1,362	1,088	-83	-274	192
	-143	-225	-139	(-602;437)	(-797;248)	(-197;581)
Hospital and outpatient clinics	2,642	3,336	4,835	-2,193	-1,500	-693
	-533	-770	-1,607	(-5,515;1,129)	(-4,945; 1,946)	(-2,534;1,147)
Overnight care	313	227	318	-5	-91	86
	-162	-138	-321	(-708;697)	(-768;586)	(-332;504)
Day center	6,135	7,190	10,506	-4,371	-3,317	-1,055
	-861	-893	-2,028	(-8,693;-50)*	(-7,635;1,002)	(-3,457;1,347)
Home care	18,109	22,468	16,555	1,554	5,912	-4,359
	-3,460	-4,340	-5,264	(-10,731;13,838)	(-7,360;19,185)	(-15,293;6,576)
Home-making services	5,085	7,732	9,420	-4,335	-1,688	-2,647
	-961	-1,384	-3,559	(-11,608;2,938)	(-9,180;5,804)	(-5,826;532)
Long term institutionalization	6,017	5,688	11,227	-5,210	-5,539	328
	-1,091	-1,149	-3,281	(-11,972;1,551)	(-12,318;1,240)	(-2,793;3,450)
Welfare services	3,043	4,050	20,784	-17,742	-16,735	-1,007
	-1,545	-2,674	-16,887	(-50,999;15,516)	(-50,184;16,715)	(-6,966;4,952)
Medications	2,220	1,867	1,766	454	100	354
	-559	-425	-677	(-1,263;2,171)	(-1,445;1,646)	(-1,020;1,727)
Informal care costs	21,475	28,042	30,852	-9,377	-2,810	-6,567
	-1,573	-1,958	-3,629	(-17,080;-1,674)**	(-10,783;5,162)	(-11,473;-1,660)***
Case management costs	3,120	2,469	N/A			
	-12	0				
Total costs	69,435	84,155	107,627	-38,192	-23,472	-14,720
	-5,980	-7,134	-23,411	(-85,606;9,222)	(-71,386;24,442)	(-33,014;3,575)

SE is standard error, CI is confidence interval, ICMM is Intensive Case Management model, LM is Linkage model, CI is confidence interval

*p = 0.047

** p = 0.017

***p = 0.009

^a^ Inverse-propensity-score-weighted generalized linear model.

### Economic evaluation

#### QALY of the person with dementia

**Fig 2:**
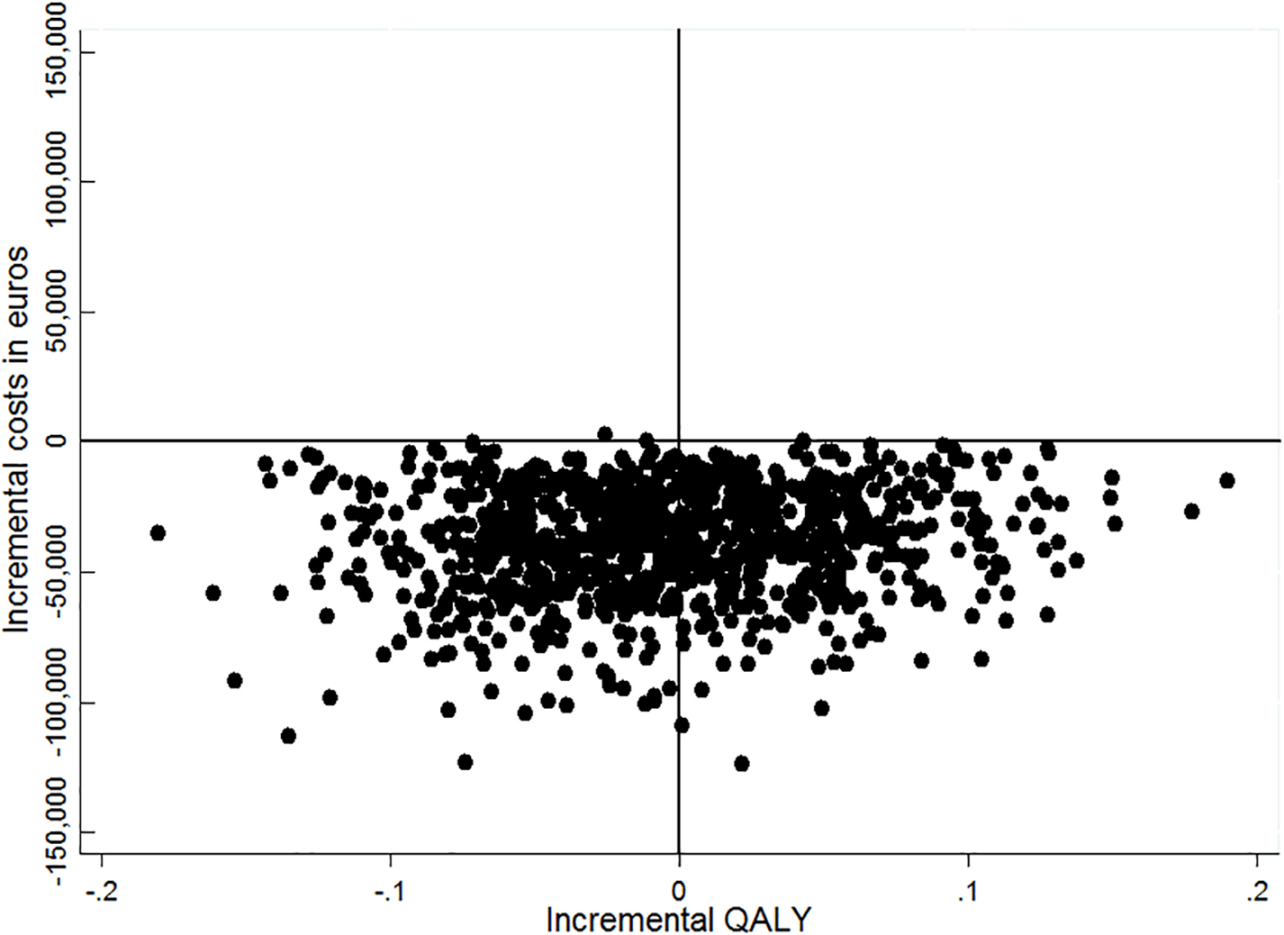
Cost-effectiveness plane. Cost-effectiveness plane for QALY of the person with dementia in the ICMM vs. control group.

**Fig 3:**
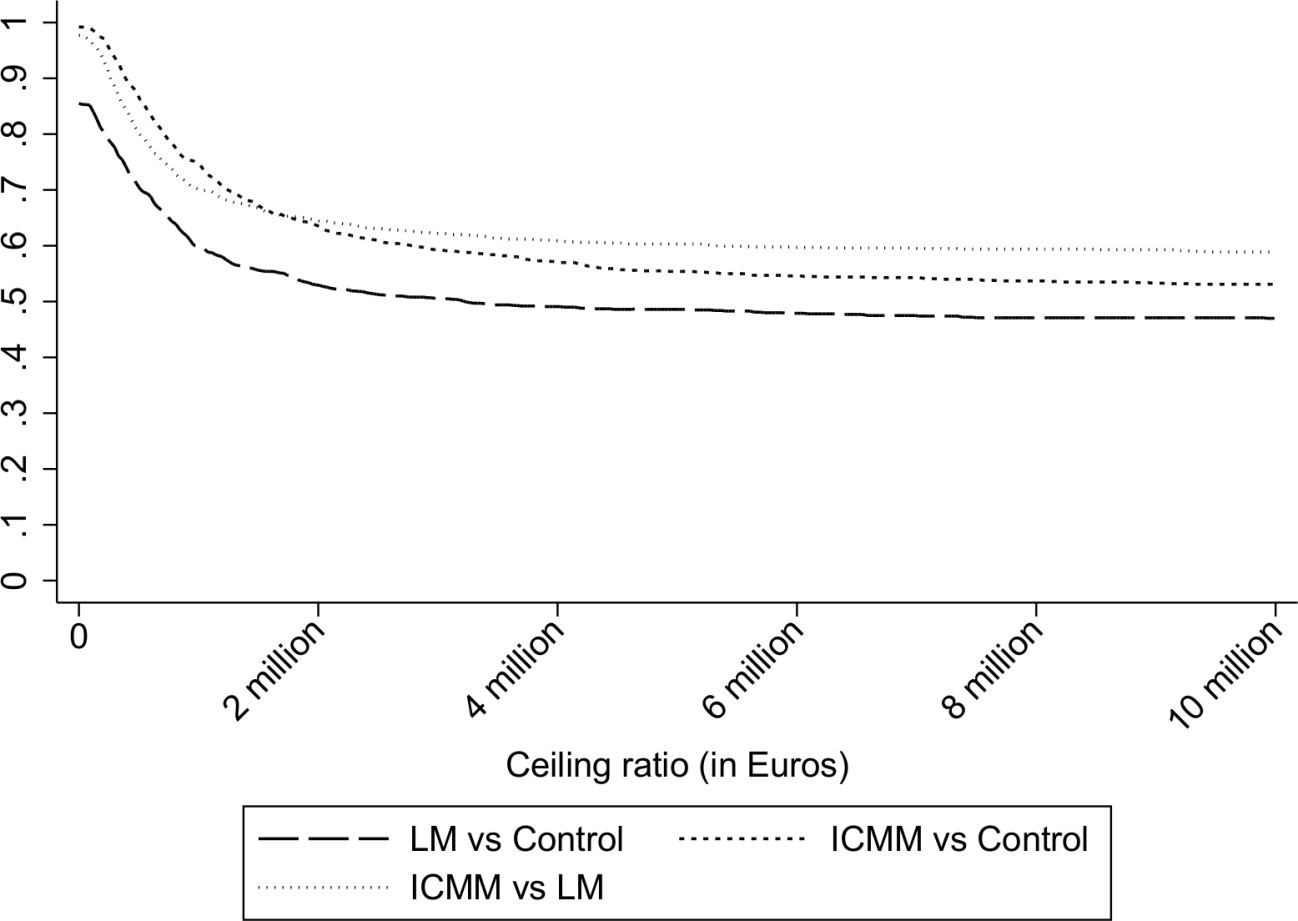
Cost-effectiveness curves. Cost-effectiveness curves for all pairwise comparisons for QALY of the person with dementia. The long dash represented the comparisons between LM versus control group. The short dash compared the cost-effectiveness of the ICMM versus Control group. Dots were used to represent the comparison of the ICMM compared to LM.

The CE plane (Fig 2) shows that the majority of the bootstrapped cost-effect pairs were located in the Southern quadrants (0.456 were in the Southeastern and 0.541 was in the Southwestern) and very few pairs were in the northern quadrants (0.001 was in the Northwestern and 0.002 in Northeastern quadrants, respectively) for the comparison of ICMM versus control confirming the non-significant difference in QALYs [42]. This was confirmed in the CEA curve as the function goes to a probability of nearly 0.50 at very high willingness-to-pay values (up to 10,000,000 €/QALY). The CEA curve showed that the probability that ICMM was cost-effective in comparison with control was 0.992 at a WTP value of 0 €/QALY and remained 0.992 at a WTP of 30,000 €/QALY (see Fig 3 for the CEAC of all comparisons). Table 4 presents the results of the economic evaluation.

**Table 4:** Adjusted mean differences in outcomes and ICERs.

	N	Adjusted mean differences in costs (EUR)	Effects	CE Plane	P_CE_ at WTP = € 0	P_CE_ at WTP = €30,000
ICMM vs. Control				ICER		
QALY^a^^,^^b^	521	-38,192	-0.004	9,581,433	0.992	0.992
		(-85,606; 9,222)	(-0.14; 0.13)			
Combined	521	-38,192	0.0004	-91,127,631	0.992	0.992
QALY^a^^,^^b^		(-85,606; 9,222)	(-0.16; 0.16)			
NPI ^c^	521	-40,490	-1.54	26,345	1	0.89
		(-89,014; 8,035)	(-7.15; 4.07)			
GHQ-12 ^c^	521	-40,490	-0.11	361,012	1	1
		(-89,014; 8,035)	(-0.95; 0.72)			
LM vs. control						
QALY^a^^,^^b^	521	-23,472	-0.01	2,236,139	0.855	0.853
		(-71,386; 24,442)	(-0.14; 0.12)			
Combined	521	-23,472	-0.03	686,587	0.84	0.855
QALY^a^^,^^b^		(-71,386; 24,442)	(-0.19; 0.12)			
NPI ^c^	521	-25,698	0.33	-78,356	1	0.63
		(-74,654; 23,257)	(-5.37; 6.02)			
GHQ-12 ^c^	521	-25,698	-0.09	288,562	1	0.98
		(-74,654; 23,257)	(-0.94; 0.76)			
ICMM vs. LM						
QALY^a^^,^^b^	521	-14,720	0.01	-2,260,818	0.977	0.977
		(-33,014; 3,575)	(-0.09; 0.10)			
Combined QALY^a^^,^^b^	521	-14,720	0.03	-425,349	0.977	0.977
		(-33,014; 3,575)	(-0.08; 0.15)			
NPI ^c^	521	-14,791	-1.86	7,932	1	0.93
		(-33,887; 4,305)	(-5.4; 1.67)			
GHQ-12 ^c^	521	-14,791	-0.02	640,340	1	0.96
		(-33,887; 4,305)	(-0.59; 0.55)			

ICMM is Intensive Case Management model, LM is Linkage model, CI is confidence interval, QALY is Quality-adjusted-life-year, ICER is Incremental Cost Effectiveness Ratio, NPI is Neuropsychiatric Inventory, GHQ-12 is General health questionnaire-12. P_CE_ = probability that the intervention is cost-effective as compared to its control condition.

^a^ Inverse-propensity-score-weighted generalized linear models

^b^ Including time after death

^c^ Inverse-propensity-score-weighted generalized estimating equations

The ICER for LM versus control was 460,135 indicating that the loss of 1 QALY in the LM is associated with cost savings of €460,135 in comparison with control. The majority of the bootstrapped cost-effect pairs were in the Southern quadrants (0.369 in the Southeastern quadrant and 0.499 in the Southwestern quadrant) and few pairs were in the Northern quadrants (0.055 in Northeastern quadrant and 0.077 in Northwestern quadrant). The CEAC showed that the probability that the LM model was cost-effective in comparison to the control group was 0.853 at a WTP value of 0 and this slightly decreased to 0.81 at a WTP value of 30,000 €/QALY, and to 0.5 at WTP of €3.24 million per QALY.

The ICER for the ICMM group versus the LM was -549,962 which indicated that there is a saving of €-549,962 per additional QALY in the ICMM as compared to the LM. The comparison between ICMM and LM showed that the cost-effect pairs were again mostly in the Southern quadrants (0.556 in southeast quadrant and 0.421 in Southwest quadrant) and a minority of pairs in the Northern quadrants (0.014 in Northeastern quadrant and 0.009 in the Northwestern quadrant). The CEAC showed that for the QALY for the person with dementia the probability that the ICMM was cost-effective in comparison to the LM model was 0.977 at a WTP value of 0 €/QALY and 0.977 at a WTP of 30,000 €/QALY.

#### Combined QALY of the person with dementia and informal caregiver

**Fig 4:**
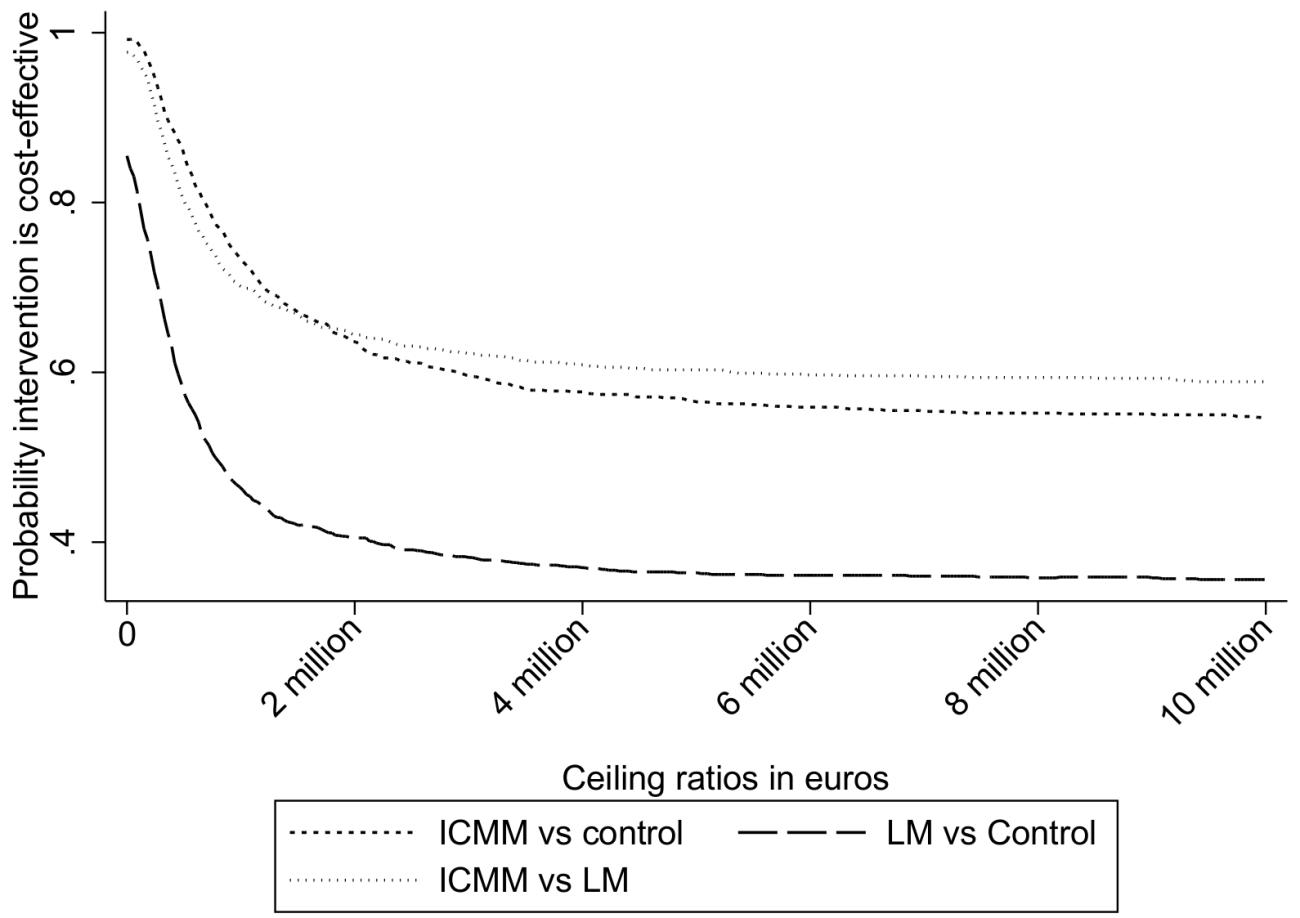
Cost acceptability curves for combined informal caregiver-person with dementia QALY for Intensive case management, the linkage and control model. Cost-effectiveness curves for all pairwise comparisons for the combined QALY of the person with dementia and informal caregiver. The long dash represented the comparisons between LM versus control group. The short dash compared the cost-effectiveness of the ICMM versus Control group. Dots were used to represent the comparison of the ICMM compared to LM. The threshold value used was 30,000 euros per QALY.

The ICER for the ICMM versus the control group was 9,495.671 indicating that the loss of one combined (informal caregiver and person with dementia) QALY there would be a cost-saving of €9,495,671. Based on the CE planes the majority of the bootstrapped cost-effect pairs were in the Southern section (0.472 in Southwestern and 0.52 in Southeastern quadrant) for the comparison between ICMM compared to the control group (see S1 Fig for CEA plane). Fig 4 presents the CEAC curves per pairwise group comparison for the combined-QALY. At WTP values of 0 and 30,000 €/QALY, the probability of cost-effectiveness for the ICMM versus control was 0.992 and this probability decreased with increasing WTP values. The ICER for the LM versus the control group indicated €430,871 in cost savings per QALY lost. The comparison between LM and the control groups showed the majority of the cost-effect pairs (see S2 Fig for CEA plane) in the Southwestern quadrant (0.559) and then smaller in amounts in the other quadrants (0.296 in Southeastern, 0.096 in Northwestern and 0.049 in Northeastern quadrants). At a WTP value of 0 €/QALY the probability of cost-effectiveness as 0.855 and this decreased to 0.5 at a WTP of 7.5 million €/QALY.

The ICER for the ICMM versus the LM was –291,741 indicating that for one combined-QALY gained, there are savings of €291,741 for the ICMM versus the LM. The majority of the cost-effect pairs were in the Southern quadrants (0.556 in the Southeastern quadrants and 0.421 in the Southwestern quadrant). The CEAC showed that for combined-QALYs the probability of cost-effectiveness for ICMM versus LM was 0.977 at WTP values of 0 and 30,000 €/QALY and that this probability decreased with increasing ceiling ratios.

#### NPI

The ICER for NPI was 26,345 indicating that a 1 point improvement on the NPI in the ICMM is associated with cost savings of €26,345 compared to the control group. For the LM versus the control group, the ICER indicated that there were savings of €78,356 per one point improvement in NPI score. For ICMM versus LM, the ICER indicates that there were savings of €7,932 per one point improvement in NPI score. For all comparisons, the CEAC was a decreasing function with increasing WTP values.

#### GHQ-12

The ICER for GHQ-12 was 361,012 indicating that a 1 point improvement on the GHQ-12 in the ICMM group was associated with cost-savings of €361,012 as compared to the control group. The ICER for the LM in comparison with control is 288,562 indicating that 1 point improvement on the GHQ-12 in the LM was associated with cost-savings of €288,562 as compared to the control group. For ICMM versus LM, the ICER was 640,340 indicating that 1 point decrease on the GHQ-12 in the ICMM was associated with cost-savings of €640,340 as compared to the LM. For all comparisons, the CEAC decreased as the WTP value increases.

## Discussion

This study evaluated the cost-effectiveness of two case management models and no access to case management (control). Informal care costs were significantly lower in the ICMM than the control and LM group. Daycare costs were also significantly lower in the ICMM group than the control group. For all outcomes, the probability that the ICMM was cost-effective in comparison with LM and the control group was larger than 0.97 at a threshold ratio of 0 €/incremental unit of effect. Cost savings were accompanied by a small non-significant negative effect on quality of life for the person with dementia in both case management groups compared to the control group. Health policy makers will have to decide whether this small negative effect on QALYs is acceptable based on the generated cost savings that the ICMM model appeared to provide.

In the clinical paper, the ICMM model appeared to have a positive impact on caregiver’s utility scores measured by the EQ-5D compared to the LM model but the differences could not be considered clinically relevant [17]. However, this difference in quality of life did not translate into a positive effect on QALYs in the economic evaluation. Total needs, met and unmet care needs were significantly less in the ICMM compared with the control group [17].

A recent Cochrane review showed that case management participants used more social care services and general practitioner consultations than control group participants but used similar amounts of health care services [43]. This review found no differences in informal care time between the case management group and usual care group [43]. In contrast, in our study we found that informal care costs in ICMM were significantly lower than in LM and control. However, like the Cochrane review, we did not find differences in long term care between groups at two years follow-up [43].

This study has several strengths. Persons with dementia and their informal caregivers were followed over a period of 2 years. This is a relatively long period of time and provides good insight into resource utilization, quality of life, NPI scores of persons with dementia and mental health of informal caregivers. Although 36 percent of persons with dementia died during this study, we included them in our analysis and, thus, the high costs that are associated with care just before death. Another strength is that the case management models were implemented for many years previous to the start of this study. Therefore, the results have high external validity. The economic evaluation was performed from a societal perspective and also included informal care costs, thereby providing detailed insight into the economic consequences of dementia care in the community.

### Limitations

#### Study design

There are also some limitations that should be considered. The observational design of the study led to baseline differences as well as possible selection bias. By using propensity scores, we tried to overcome this limitation in the analyses. There was potentially more heterogeneity in the people recruited into this study compared to a randomized control trial which may have resulted in greater uncertainty around outcomes.

#### Use of the EQ-5D

Another possible limitation is the use of the EQ-5D to measure quality of life, as it may not be sensitive enough to pick up relevant changes. Furthermore, the EQ 5D for the person with dementia was completed by a proxy (the informal caregiver). Approximately 263 persons with dementia also filled in EQ 5Ds themselves, but these were not used in the economic evaluations because the data was not sufficiently complete and prone to bias. Based on the paper of McPhail et al [24] we carefully rephrased the EQ-5D questions for the proxy-report by the informal caregiver. We evaluated the dyadic effect of case management on both the informal caregiver and the person with dementia by adding the two QALYs together Based on theory, QALYs can be aggregated across individuals (A QALY reflects a QALY regardless of who loses it or gains it) [44]. However, there is a risk that this may lead to double counting if the caregiver adjusts his/her health state valuation to incorporate some of the impact on the person with dementia or the other way around. However, we are not aware of any studies showing evidence of this effect.

#### Content of case management

Based on this study, we are not able to identify specific components of case management that drive the cost-savings associated with case management as compared to control. Although we did publish a process analysis on the barriers and implementation of case management [18] and interviewed case managers to understand the content of their roles, we did not review care plans and evaluate multidisciplinary meetings content.

#### Measuring informal care

Our results show that the savings in informal care costs are the main contributor to societal cost savings. It is possible that the case manager is effective in helping informal caregivers to find alternatives to informal care for their loved one, although this was not reflected by our cost estimates since daycare costs increased in the ICMM group compared to the control group. However, the costs of home care were increased in the case management groups as compared to control which may indicate a shift from informal care to home care.

Future research should evaluate what the case manager actually does and which components of case management are effective. Moreover, since informal care time is notoriously hard to measure, future studies should find more reliable methods to measure informal care time accurately.

#### Cost measurement

Another potential limitation is that we used cost diaries to measure costs. We tried to limit recall bias in the assessment of healthcare utilization rates by collecting these data prospectively. We collected the cost diaries at each follow up visit during which the diaries were checked for completeness.

#### Study power

Moreover, because cost-effectiveness was not the primary outcome of the original study our results may be under-powered. However, it is for this reason that in economic evaluations the focus is on estimation of intervention effects instead of on hypothesis testing so that they can still provide useful information even when they are underpowered [45].

This study provides preliminary evidence that the intensive case management model is cost-effective compared to a control group and the LM model as it appears to decrease total costs. However, further research is still needed, since this was not a randomized controlled trial. Therefore, all results should be interpreted with caution. Despite this methodological limitation, results of the study are quite robust in showing that ICMM is cost-effective as compared to LM and control.

## Supporting Information

S1 Appendix Background article Comparing Dutch case management care models for people with dementia and their caregivers: The design of the COMPAS study. (PDF)

S2 Appendix Background article Community-Dwelling Persons with Dementia and Their Informal Caregivers With and Without Case Management: 2-Year Outcomes of a Pragmatic Trial. (PDF)

S1 Case Record Form Caregiver interview file. Interview with informal caregiver (in Dutch).
(DOC)

S2 Case Record Form Person with dementia interview file. Interview with the person with dementia (in Dutch).
(DOC)

S3 Case Record Form Caregiver questionnaire file. Questionnaire filled in by informal caregiver (in Dutch).
(DOC)

S4 Case Record Form Cost diary file. Cost diary filled in by the informal caregiver (in Dutch).
(DOC)

S1 Dataset Combined data set wide. (DTA)

S2 Dataset Bootstrapped file for pwd QALY reference control group. (DTA)

S3 Dataset Bootstrapped file for pwd QALY reference LM. (DTA)

S4 Dataset Bootstrapped file for combined QALY reference control group. (DTA)

S5 Dataset Bootstrapped file for combined QALY reference LM group. (DTA)

S6 Dataset CEAC curve for pwd QALY. (DTA)

S7 Dataset CEAC curve dataset for combined QALY. (DTA)

S1 Fig CEA plane for the combined QALY ICM vs control. (TIF)

S2 Fig CEA plane for the combined QALY LM vs control. (TIF)

S1 Stata Syntax The syntax do file. (DO)

S1 Table Table of cost prices. Unit costs used for economic evaluation. All prices were adjusted for the year 2010 using consumer price index figures [26]. Healthcare utilization, and absenteeism from paid and unpaid work was valued using Dutch standard costs [27]. Costs of medications were valued using prices from the Royal Dutch Society for Pharmacy [28].
(DOCX)

S2 Table Characteristics of different care models. (DOCX)
