# Supplementary material for: The Cost-Effectiveness of Two Forms of Case Management Compared to a Control Group for Persons with Dementia and Their Informal Caregivers from a Societal Perspective
Source: PLoS One. 2016 Sep 21;11(9):e0160908. doi: 10.1371/journal.pone.0160908 (PMC5031395; doi:10.1371/journal.pone.0160908)
Supplement: S2 Appendix — (PDF) [file pone.0160908.s002.pdf]

JAMDA xxx (2015) e1–e8

ELSEVIER

JAMDA

journal homepage: www.jamda.com

Original Study

Community-Dwelling Patients With Dementia and Their Informal Caregivers With and Without Case Management: 2-Year Outcomes of a Pragmatic Trial

Janet MacNeil Vroomen MSc [a,*], Judith E. Bosmans PhD [b], Peter M. van de Ven PhD [c], Karlijn J. Joling PhD [d], Lisa D. van Mierlo PhD [d,e], Franka J.M. Meiland PhD [d,e], Eric P. Moll van Charante MD, PhD [f], Hein P.J. van Hout PhD [d], Sophia E. de Rooij MD, PhD [a,g]

[a] *Section of Geriatric Medicine, Department of Internal Medicine, Academic Medical Center, University of Amsterdam, Amsterdam, The Netherlands*
[b] *Faculty of Earth and Life Sciences, Department of Health Sciences and the EMGO Institute for Health and Care Research, VU University Amsterdam, Amsterdam, The Netherlands*
[c] *Department of Epidemiology and Biostatistics, VU University Medical Center, Amsterdam, The Netherlands*
[d] *Department of General Practice and Elderly Care Medicine and the EMGO Institute for Health and Care Research, VU University Medical Center, Amsterdam, The Netherlands*
[e] *Department of Psychiatry and the EMGO Institute for Health and Care Research, Alzheimer Center, VU University Medical Center, Amsterdam, The Netherlands*
[f] *Department of General Practice, Academic Medical Center, University of Amsterdam, Amsterdam, The Netherlands*
[g] *University Center of Geriatric Medicine, University Medical Center Groningen, Groningen, The Netherlands*

Keywords:
Case management
longitudinal study
dementia
informal caregivers

ABSTRACT

Objective: To evaluate outcomes for persons with dementia and primary informal caregivers of 2 types of implemented case management (intensive case management [ICMM] and linkage [LM] models) with no case management (control group).
Design: A pragmatic trial using a prospective, observational, controlled, cohort study.
Setting: Community care in the Netherlands.
Participants: A total of 521 dyads.
Intervention: Case management provided within one care organization (ICMM), case management where multiple case management organizations are present within one region (LM), and a group with no access to case management (control).
Measurements: Neuropsychiatric problems in persons with dementia assessed by the Neuropsychiatric Inventory (NPI) and psychological health in informal caregivers as measured with the General Health Questionnaire (GHQ-12). Secondary outcomes included care and support needs, quality of life, and institutionalization. Comparability of groups at baseline was secured by inverse-propensity-score-weighted mixed models.
Results: No significant differences in changes in total NPI or GHQ-12 scores between the groups over 2 years were found. Secondary outcomes showed better quality-of-life scores for informal caregivers in the ICMM than the LM. Total needs, met and unmet care needs were significantly less in the ICMM compared with the control group.
Conclusion: Neither case management type affected clinical outcomes of dyads meaningfully. The ICMM has positive impact on caregivers' quality of life and patient's number of needs compared with persons in LM and persons without access to case management respectively.

© 2015 AMDA – The Society for Post-Acute and Long-Term Care Medicine.

Dementia is a chronic and devastating disorder marked by memory loss, cognitive impairment, and behavioral lapses resulting in pronounced consequences for the people with dementia, their families, and society.

A systematic review reported a global dementia prevalence of 48.1 million for 2020 and approximately 90.3 million in 2040.[1] The review

The authors declare no conflicts of interest.

Research funding was provided by a governmental grant by the Dutch National Programme for Improving Care for Older persons (ZonMw no. 313080201). The Medical Ethics Committee of the VU University medical center approved the study protocol.

* Address correspondence to Janet MacNeil Vroomen, MSc, Academic Medical Center, Internal Medicine, Meibergdreef 9, PO Box 22660, Amsterdam, 1100 DD, The Netherlands.

E-mail address: j.l.macneil-vroomen@amc.uva.nl (J. MacNeil Vroomen).

http://dx.doi.org/10.1016/j.jamda.2015.06.011
1525-8610/© 2015 AMDA – The Society for Post-Acute and Long-Term Care Medicine.

noted that in high-income countries, the numbers of people with dementia will continue to grow and the provision and financing of measures to meet their long-term care needs, including support for their informal caregivers, will become an increasingly important societal and political issue.[1]

Studies show that people age 65 and older survive an average of 4 to 8 years after a diagnosis of Alzheimer disease, and some live as long as 20 years.[2] Although there are a variety of services available for community-dwelling people with dementia and their informal caregivers, people often lack information regarding all available services that may address their care needs.[3] Also, many experience insufficient alignment, management, and continuity of care and support during the disease trajectory.[4]

Several studies have evaluated the effects of case management in many countries.[5–8] However, reviews and meta-analyses have yielded inconsistent results regarding case management on patient and caregiver outcomes in care satisfaction, institutionalization, hospitalization, caregiver burden, depression, and economic outcomes.[5–7,9]

In the Netherlands, various models of case management are implemented. These differ with respect to how services are delivered and by whom, the training provided to its staff, and the means of financing. In the COMPAS study (Case management of dementia patients and their caregivers), the 2 most prominent Dutch case management models, the intensive case management model (ICMM) and the linkage model (LM), are compared with care in regions where there was no access to case management (control group) and with each other. The objective of this current article was to compare clinical effects for persons with dementia and their informal caregivers over 2 years between the 2 case management models and the control group.

Design

Study Design

This study was a prospective, observational, controlled, cohort study to evaluate the clinical effects of 2 case management models and care in regions where patients have no access to case management (control group). Persons with dementia and their primary informal caregivers were followed for 2 years. The Medical Ethics Committee of the VU University medical center approved the study protocol.

Participants and Setting

Persons with dementia and their primary informal caregivers were recruited from various regions of the Netherlands. Persons with dementia were eligible for this study if they lived at home, had a diagnosis of dementia, were not terminally ill, were not anticipated to be admitted to a long-term care facility within 6 months, and had an informal caregiver. The informal caregivers were eligible if they were the primary informal caregiver responsible for looking after the patient, had sufficient language proficiency, and were not severely ill. In case management regions, case managers of the participating organizations provided lists of their patients who met these criteria. In the control group, recruitment took place via outpatient geriatric or neurologic (memory) clinics, Alzheimer centers, and general medical practices. Recruitment areas included rural areas in the north of Netherlands, semi-rural areas outside of Amsterdam, and urban areas such as Amsterdam.

Care Models

Case management is defined as “a collaborative process in which a case manager assesses, plans, implements, coordinates, monitors and evaluates all options and services required to meet an individual’s health, social care, educational and employment needs, using communication and available resources to promote quality and cost effective outcomes.”[10]

The case management models that were evaluated in this study as well as the content of care in regions without case management were described in detail elsewhere.[11,12] We therefore summarize their most important characteristics only (see Table 1).

The LM is a collaboration between independent care providers (eg, home care organizations, general practitioners, social care services) who were already providing health care services in the region and who then were given the mandate to initiate case management services. After a formal diagnosis, persons with dementia are connected to a case manager who provides educational, emotional, and practical support, such as advice on disease-related issues, and recommends supportive health and social services until time of nursing home admission or death of the patient. In general, caregivers are involved in this process whenever possible. Expert advice can be sought through multidisciplinary meetings held regularly with experts from the various collaborating organizations. Case managers often work 2 job positions; therefore, they only work part-time as a case manager and have another part-time position such as a district nurse.[12] Van Mierlo et al[12] found that the case managers in the LM group often had to deal with time constraints and issues around multitasking. The mean case load of a case manager based on one full-time equivalent (36 hours over 5 days) was 53.9 (SD 23.3).

Case managers in the ICMM are appointed to one organization that is specialized in dementia care. They guide and support people with dementia for long periods of time mostly starting after diagnosis, and offer medical and psychosocial services from their organization.[13] The case manager works in collaboration with an “in-home” multidisciplinary team to tailor care needs of the person with dementia and the informal caregiver.[13] Most of the case managers in the ICMM group work full-time and are expected to have more focus on their patients compared with case managers in LM.[12] The mean case load of a case manager in the ICMM group was 61.6 (SD 16.7) per full-time equivalent, which is not significantly different from the case load in the LM group.

The control group was recruited in areas without access to a case manager.[14] In some cases, care may be monitored by a registered nurse working in the general practice in addition to the general practitioner. In these regions, no central coordination of dementia care is provided by a specific health care professional. Care is usually initiated by the patient, his or her informal caregiver, or health care provider involved in the care for the patient dependent on local service configuration. Access to home or respite care did not differ across regions.

Data Collection

Persons with dementia and their primary informal caregivers were interviewed at their homes by trained research interviewers using case record forms. Before the baseline interviews, all participants signed an informed consent form. The informal caregiver signed on behalf of the person with dementia if he or she was unable to understand and reproduce the study goals. Interviews and questionnaires were completed at baseline, and 6, 12, 18, and 24 months. When people dropped out of the study we asked if they could complete an exit interview that contained reasons for dropping out as well as the General Health Questionnaire (GHQ-12), EuroQol-5 Dimensions (EQ-5D) for the informal caregiver, and relevant care resource utilization questions. We also collected the date of institutionalization or death if that was the reason for dropout.

Table 1
Baseline Table of Characteristics of Care Models, Persons With Dementia, and Informal Caregivers

	ICMM	LM	Control	Total Group	*P*
Persons with dementia	n = 234	n = 214	n = 73	n = 521	
Age, mean (SD)*	79.9 (7.7)	81.0 (7.5)	75.9 (8.7)	79.8 (7.9)	<.001
Female gender, n (%)†	122 (52.4)	134 (62.6)	32 (43.8)	288 (55.3)	.009
Married or in a relationship, n (%)†	128 (56.4)	98 (47.8)	51 (70.8)	277 (55.0)	.003
Living situation, n (%)†					.065
Living alone	92 (40.5)	95 (46.3)	19 (26.8)	206 (41.0)	
Living with another person	130 (57.3)	105 (51.2)	49 (69.0)	284 (56.5)	
Living in an elderly home	5 (2)	5 (2.4)	3 (4.2)	13 (2.6)	
Born in the Netherlands, n (%)†	209 (92.1)	178 (86.8)	64 (88.9)	451 (89.5)	.204
Education, n (%)†					.011
Elementary/lower	93 (41.9)	99 (49.5)	21 (29.6)	213 (43.2)	
Secondary	111 (50.0)	81 (40.5)	37 (52.1)	229 (46.5)	
Higher/University	18 (8.1)	20 (10.0)	13 (18.3)	51 (10.3)	
MMSE, mean (0–30‖) (SD)*	19.6 (5.5)	18.7 (6.4)	20.4 (4.8)	19.3 (5.8)	.150
Time since symptoms in years, median (IQR)‡	3.5 (2.0–5.0)	3.8 (2.0–5.3)	4 (2.7–5.5)	3.7 (2.0–5.2)	.641
Time since diagnosis in years, median (IQR)‡	2.4 (1.4–3.7)	2.1 (1.3–3.3)	2.0 (1.3–3.0)	2.3 (1.3–3.5)	.267
Time in case management, y, median (IQR)§	2.1 (1.3–3.1)	1.7 (0.42–2.5)	NA	1.8 (1.1–2.8)	<.001
Multimorbidity (more than 2 diseases), n (%)†	203 (88.7)	172 (83.5)	55 (76.4)	430 (84.8)	.032
Utility from the EQ-5D-Proxy (0–1)‖ (SD)	0.74 (0.2)	0.71 (0.3)	0.74 (0.2)	0.73 (0.23)	.299
EQ-5D utility from persons with dementia (0–1) (SD)	0.82 (0.2)	0.79 (0.2)	0.83 (0.2)	0.81 (0.21)	.415
QOL-AD proxy (13–52) (SD)	31.89 (5.1)	31.73 (5.2)	32.70 (5.0)	32.0 (5.1)	.483
Informal Caregivers					
Age (SD)*	64.5 (12.8)	64.4 (12.4)	65.8 (11.7)	64.6 (12.5)	.687
Female gender, n (%)†	163 (70.0)	136 (63.6)	49 (67.1)	348 (66.8)	.390
Spouse of the person with dementia, n (%)†	122 (53.3)	94 (45.6)	50 (69.4)	266 (52.5)	.002
Living together with person with dementia, n (%)†	127 (55.5)	100 (48.8)	50 (70.4)	277 (54.9)	.007
Multimorbidity (1 or more diseases), n (%)†	149 (65.1)	119 (57.8)	50 (69.4)	318 (62.7)	.129
Education, n (%)†					.370
Elementary/lower	36 (16.0)	31 (15.3)	10 (13.9)	77 (15.4)	
Secondary	139 (61.8)	127 (62.6)	38 (52.8)	304 (60.8)	
Higher/University	50 (22.2)	45 (22.2)	24 (33.3)	119 (23.8)	
EQ-5Dd utility (0–1) (SD)	0.83 (0.2)	0.85 (0.2)	0.86 (0.2)	0.84 (0.21)	.261

IQR, interquartile range.
*One-way analysis of variance.
†χ^2 test.
‡Kruskall-Wallis test.
§Mann-Whitney test.
‖The underlined scores indicate the more positive outcomes.

Outcome Measurements at the Person With Dementia Level

Information on the time of first symptoms, the dementia diagnosis, and other baseline information was gathered from the informal caregiver.

The primary outcome in the person with dementia was the presence of neuropsychiatric symptoms as measured with the Neuropsychiatric Inventory (NPI), which assesses 12 neuropsychiatric domains in persons with dementia.[15] The NPI was rated by a caregiver familiar with the person with dementia's behavior.[15] It assesses presence, frequency, severity, and the symptom specific caregiver distress in the previous month.[15] Calculation of the total score is the sum of the 12 domain scores, which ranges from 0 to 144 points with higher scores indicating more problems.[15]

Secondary outcomes in persons with dementia included institutionalization, death, quality of life measured with the Quality of Life-Alzheimer's Disease (QOL-AD),[16] care and support (met and unmet) needs that were measured with the Camberwell Assessment of Needs for the Elderly (CANE),[17] and performance of basic activities of daily life as measured with the original Katz activities of daily living (ADL) index score (KATZ-6) and the modified Katz ADL index (15 ADL+instrumental ADL items).[18] Information regarding (date of) institutionalization was collected via the informal caregiver if relevant.

Outcome Measurements at the Informal Caregiver Level

The primary outcome in the primary informal caregiver was severity of psychological health as measured by the GHQ-12.[19] Secondary outcomes included health-related quality of life (as measured by the EQ5D[20]), feelings of mastery (the Pearlin mastery scale[21]), sense of competence (the short sense of competence scale, SSCQ[22]), loneliness (the "Jong-Gierveld" loneliness scale[23]), and care burden, as measured with the CarerQOL.[24] The EQ-5D includes 5 dimensions: mobility, self-care, usual activities, pain/discomfort, and anxiety/depression.[20] The respondent answers each of the EQ-5D's 5 dimensions with 1 of 3 possible responses: "no problems," "some problems," or "severe problems." The set of 5 responses defines a health state. The 243 (3^5) possible health states are weighted using a valuation set from a sample of the Dutch general population known as the Dutch EQ-5D tariff,[25] resulting in a utility score. This utility reflects the relative desirability of a particular health state and is measured on a scale in which 0 refers to death and 1 refers to perfect health. The health states from the EQ-5D were subsequently converted to utilities using the Dutch tariff.[25]

Data Analyses

Baseline demographic, clinical, and prognostic characteristics among the 3 groups were compared using χ^2 tests for categorical variables, analysis of variance for continuous variables, and Kruskall-Wallis or Mann-Whitney *U* tests if the baseline variables were skewed.

As this was a nonrandomized study, advanced statistical methods were needed to control for any baseline imbalances between the different treatment groups.[26] First, propensity scores were calculated using generalized boosted methods for multiple treatments using the

ARTICLE IN PRESS

twang package in R.[27] Balance and overlap of propensity score distributions of the 3 treatment groups were assessed. The twang package provides propensity score–based weights for the estimation of the average treatment effects (ATE) for more than 2 treatment groups. All covariates where groups differed on baseline or that were associated with the baseline NPI total score were included in the calculation of propensity score and weights. The propensity score–based weights were then exported to Stata (Stata Corp, College Station, TX) to be used as sampling weights. As we had longitudinal data with repeated measurements for each pair of participants, we assumed equal weighing for all measurements within a pair.

Differences in change over time in outcomes were compared between groups using multilevel models with 2 levels: measurements nested within pairs. All models included a main effect of treatment group, a linear term for time, and the interaction between time and treatment group. In cases in which the interaction between group and time was not significant, a simpler model was fitted including only a main effect of treatment group and a linear effect of time (assuming equal changes over time in the groups). Normality of residuals was assessed by means of QQ-plots. Estimated means were plotted as a function of time separately for the 3 treatment groups.

A sensitivity analysis was performed by re-rerunning the main analysis after exclusion of persons that switched treatment group during the 2-year study.

Results

Participant Recruitment and Flow Chart

Participants were recruited from April 2011 to November 2012. Figure 1 reports the number of individuals who were approached. Of the 2810 caregiver and patient pairs assessed for eligibility, 1628 met all inclusion criteria and were sent recruitment letters. A total of 521 of these individuals agreed to participate (32%) and 1107 (68%) refused to participate. We had information on the gender of the person with dementia, the gender of the caregiver, and the relationship of the informal caregiver to the person with dementia for 1172 (72%) people who were approached. The only difference we found was that informal caregivers who were the partner to the person with dementia were more willing to participate than those who had another type of relationship ($\chi^2 = 11$, df = 1, $P < .001$).

The number of participants that dropped out was 207 (40%). The main reason for dropout was death (36%). Variables associated with dropout included lower Mini-Mental State Examination score, older informal caregiver, older persons with dementia who were men, and poorer Katz scores at baseline.There was no significant difference in the number of dropouts among the 3 groups. There was no difference in rates of institutionalization or death among groups.

Table 1 presents an overview of the baseline characteristics of the persons with dementia and their caregivers. Persons with dementia in the case management models were more likely to be older, women, and not married, and had lower education and greater multimorbidity than those in the control group. Two-thirds (n = 348, 67%) of the informal caregivers were women. In the control group, the informal caregiver was more often a spouse as compared with the case management groups ($\chi^2 = 12.24$, df = 2, $P = .002$). Seventy percent of the informal caregivers in the control group lived with the person with dementia compared with the LM group (49%) or the ICMM (55%) ($\chi^2 = 10.03$, df = 2, $P = .007$).

Outcomes for the Person With Dementia

Estimated means of the total NPI scores over time are plotted over 2 years (Figure 2). No differences in the rate of change of NPI scores over time between the care groups were found. Differences between the means could therefore be assumed to be constant over time and were estimated to be −0.7 (95% −4.8–3.5) for ICMM versus control, and 1.7 (95% −2.5–5.8) for the LM versus the control group, and 2.3 (95% −0.8–5.4) for LM versus ICMM (Figure 2). The results of the secondary outcomes are available in the supplementary section.

The secondary outcomes are reported in the supplementary table and summarized here. Total, met, and unmet care needs were less in the ICMM group compared with the control group. Total met needs were less in the LM group versus the control group. This indicates that the control group had more needs overall than the intensive case management group. All other comparisons were not statistically significant.

Outcomes for Informal Caregiver

Means scores on the GHQ-12 are plotted in Figure 3. Mean GHQ-12 increased at a faster rate (indicating more psychological complaints) in persons in the control group compared with the ICMM group (time by treatment interaction 0.5 points per year, 95% CI 0.01–1.03, $P = .047$), but with the Bonferroni correction this difference is no longer significant using the prespecified alpha. There was no difference in mean scores over time between the control group compared with LM group or the LM group versus the ICMM group. The results of the secondary outcomes are available in the supplementary section.

Mean utility scores for the informal caregiver were 0.02 points per year higher in the ICMM more than the LM group (95% CI −0.01 to −0.04, $P = .0012$).

Sensitivity Analysis

We repeated the original analyses excluding the observations of persons with dementia that switched care groups during the study (n = 13). We found no differences in the results for neuropsychiatric symptoms (NPI). Differences between the means could therefore be assumed to be constant over time and estimated to be 0.9 (95% CI −3.4–5.1) for ICMM versus control, and 3.3 (95% CI −1.0–7.5) for the LM versus the control group, 2.4 (95% CI −0.7–5.4) and for LM versus IC.

Differences between the GHQ-12 means were not significant and could therefore be assumed to be constant over time and estimated to be −0.2 (95% CI −1.0–0.6) for ICMM versus control, and −0.2 (95% CI −1.1–0.6) for the LM versus the control group, −0.03 (95% CI −0.6–0.5) and for LM versus IC.

Discussion

Key Results

Few studies exist that evaluate clinical outcomes in pairs of persons with dementia and their primary informal caregiver receiving 2 types of case management versus pairs in a control group with no access to case management. The analyses showed no differences in NPI scores in persons with dementia between the control group, the ICMM group and the LM. However, in informal caregivers, the control group performed worse on the GHQ-12 than the ICMM group although the significant effect was lost when corrected for multiple groups and when persons who switched groups during the study were excluded. There was no difference in psychological health (GHQ-12 scores) between the LM group and the control group or the LM versus the ICMM group. In all 3 groups, mean GHQ-12 scores were far above the suggested cutoff point for the GHQ-12 of 1 or 2 points to

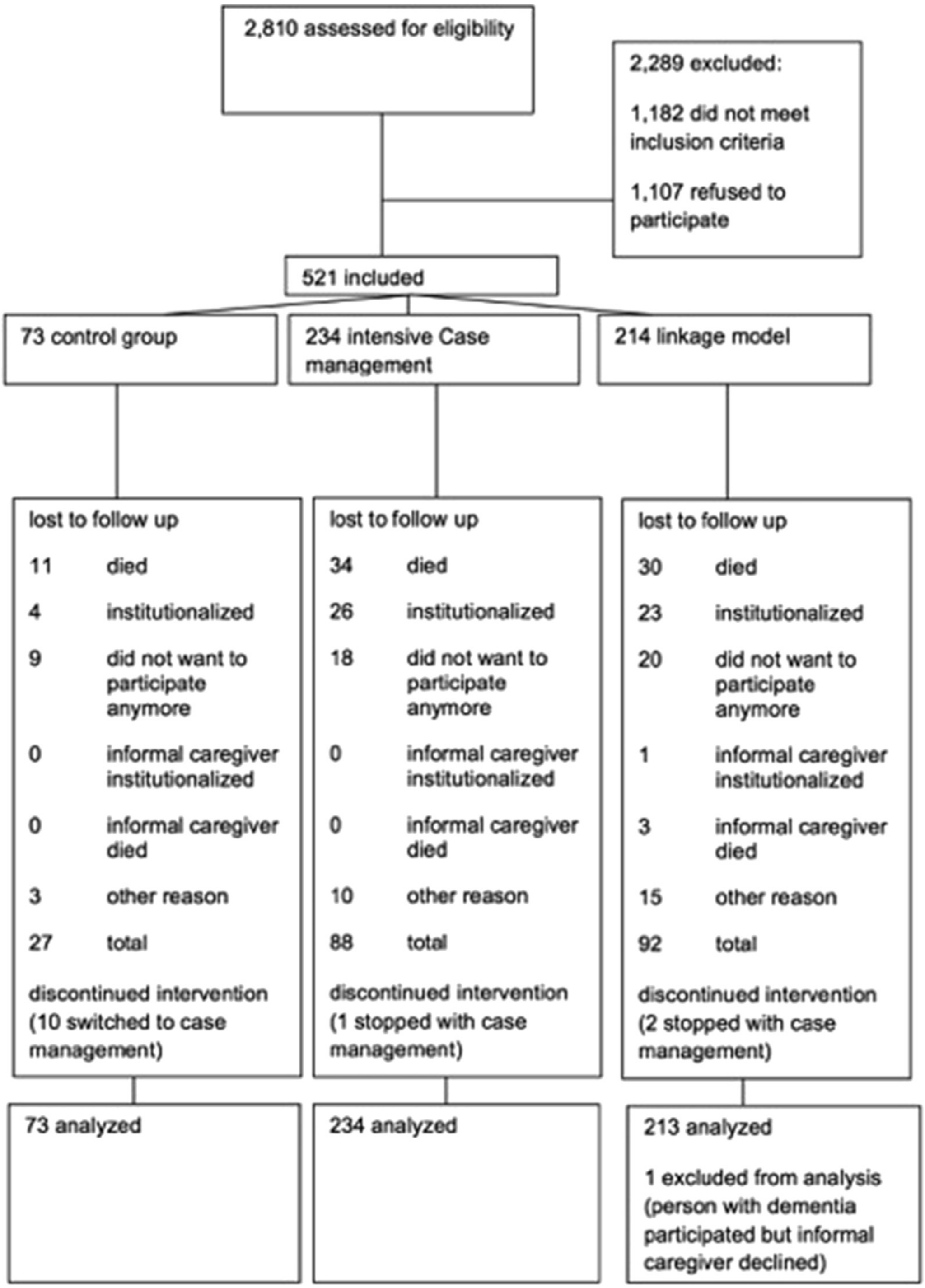

Fig. 1. Recruitment overview.

identify people with mental health problems,[28,29] which indicates that the informal caregivers were already at increased risk of mental health problems at the start of the study.

Comparison With Literature

Few studies exist that compare different case management models with general practitioner–based care.[30] In the study by Newcomer et al,[30] there were 2 types of case management compared against general practitioner–based care. They had a randomized study design and primary outcomes were caregiver burden and depression. The distinction between case management models was based on ratios of patients to case managers. Newcomer et al[30] found no differences between groups on primary outcomes. In our study, the case management provided starts at different times in the disease spectrum (either before or after dementia diagnosis), as well as by the type of organization providing case management. Usual care in the Netherlands includes case management and care supervised by only

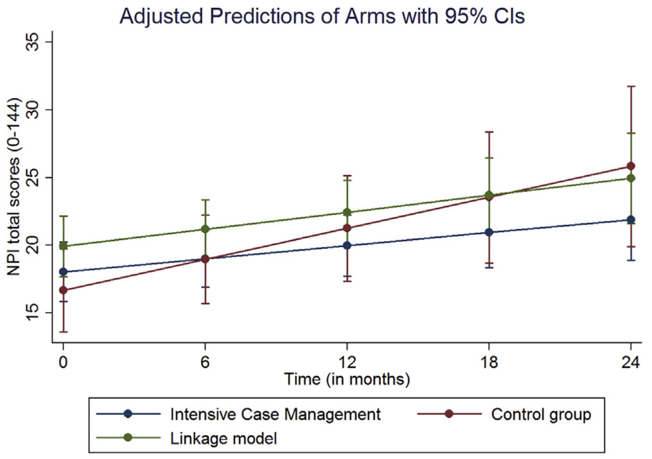

Fig. 2. Adjusted NPI scores with 95% CIs. Intensive Case Management mean NPI baseline value is 18.1 (95% CI 15.9–20.2), and the estimated mean increase is 1.9 of a point per year (95% CI 0.6–3.2). The mean baseline value of the control group is 16.7 (95% CI 13.6–19.7), and estimated mean increase is 4.6 of a point per year (95% CI 1.9–7.2). The LM has a mean baseline value of 19.9 (95% CI 17.7–22.2), and the estimated mean increase is 2.5 of a point per year (95% CI 1.0–4.1).

the general practitioner is uncommon, which is the opposite in Newcomer et al.[30]

Previously, several reviews and meta-analyses have been performed to evaluate the effects of case management in many different countries on a variety of outcomes.[5–7] Callahan et al[31] compared case management to a control group in the United States and showed a decrease in NPI scores at 12 months in the case management group, whereas in our study over 2 years the NPI total score went up in all 3 groups. A possible alternative explanation for the effect of Callahan et al[31] was the large amount of antipsychotics prescribed in the intervention group, so it might reflect not so much the psychosocial but the medical component of case management. It is possible that participants in our study had more behavior problems, as total NPI mean baseline scores were higher in our population compared with the patient population in Callahan et al.[31]

A review that included both qualitative and quantitative studies looked at facilitators and barriers to successful case management implementation in the primary health care setting.[9] Results indicated that high-intensity case management was necessary and sufficient to produce positive clinical outcomes for patients and to optimize service use. The reviewers described high-intensity case management as (1) a caseload of fewer than 50 patients per full-time equivalence, (2) regular meetings with the informal caregiver and the patient with at least 50% of these meetings being face to face, (3) education on health conditions, (4) close contact with family physicians,[9,32] and (5) proactive and timely follow-up[9,33] and following up with patients during hospitalizations and short-term institutionalizations.[9,34] In our study, both case management groups exceed this case load recommendation and it is unclear how they rate on the other factors. The average caseloads being over the recommendation of the review could be a possible reason for there being little difference among all groups. The status quo in the Netherlands is to have access to case management as usual care, although the coverage and delivery varies widely. This study shows that there are some benefits to case management but caregivers' needs are not sufficiently addressed, as shown by the impaired psychological health of the informal caregiver.

Reviews of case management show mixed results, which may be due to the complex nature of the number of psychometric scales used to assess the effect of the interventions and the individualized customization of interventions to the needs of the informal caregiver and the person with dementia.[6,35]

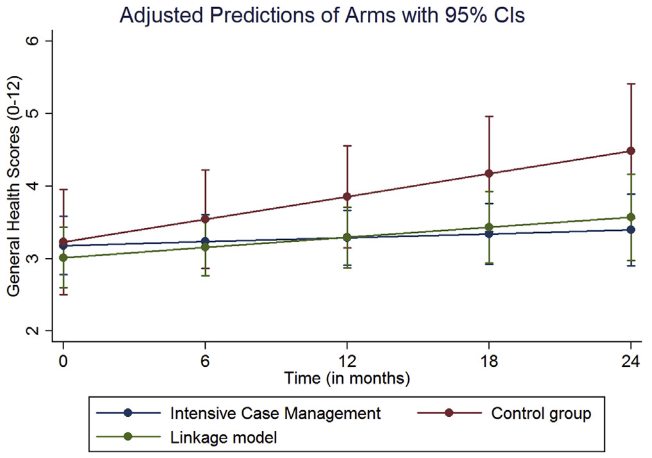

Fig. 3. Adjusted General Health Questionnaire-12 scores with 95% CIs. Intensive Case Management mean GHQ-12 baseline value is 3.2 (95% CI 2.8–3.6), and the estimated mean increase is 0.1 of a point per year (95% CI −0.1–0.4). The mean baseline value of the control group is 3.2 (95% CI 2.5–4.0), and estimated mean increase is 0.6 of a point per year (95% CI 0.2–1.1). The LM mean baseline value is 3.0 (95% CI 2.6–3.4), and the estimated mean increase is 0.3 of a point per year (95% CI −0.02–0.6).

Limitations and Strengths

The Dutch Ministry of Healthcare stipulated in 2008 that "any form of case management" had to be part of usual care for persons with dementia in all regions in the Netherlands at the end of 2011. By 2011, case management was available in most regions and subregions, although there were still regions where case management was not yet implemented or had limited capacity. Moreover, 14% of the participants in the control group switched into case management over time. However, a sensitivity analysis excluding these participants led to the same results. The observational design of the study may have led to baseline differences as well as selection bias. By using propensity scores, we tried to overcome this in the analyses. As we were comparing multiple groups, we took a conservative estimate by using a Bonferroni correction, although this results in a loss of power.

In this study, persons with dementia and their informal caregivers were followed over a period of 2 years. This is a relatively long period of time and provides good insight into the course of NPI scores of persons with dementia and mental health of informal caregivers over time. Despite this long period of follow-up, dropout levels were relatively low. Another strength is that the case management models were implemented for many years already at the time of this study. Therefore, the results have high external validity. The wide range of secondary outcomes assessed in this study provides relevant insights into the problems of persons with dementia and their informal caregivers. Finally, the use of multilevel analyses with an adjustment for time allowed us to use all available data and to estimate effects over time.

Implications for Research and Practice

Our results show that there were no differences in NPI scores between the 2 case management groups and the control group. Although GHQ-12 scores in the ICMM were better than in the control group, this difference was not statistically significant when taking into account the necessary Bonferroni correction. It is widely known

ARTICLE IN PRESS

in the literature that prolonged high stress levels in caregivers is one of the highest risk factors for nursing home admission.[36,37] The fact that GHQ-12 scores were above the cutoff scores during all times and interviews indicates that all caregivers experienced high psychological stress levels. Therefore, a future research agenda based on reducing informal caregiver stress is imperative. This is a problem that will only become worse, as it is often the preference of the family and the government for informal caregivers to provide care for persons with dementia as long as possible.

We would recommend policy makers to consider decreasing the caseloads per full-time equivalent and increase the interaction with the informal caregiver as recommended by the mixed review by Khanassov et al.[9] When we look at effective ways to deal with caregiver burden there have been no clear solutions. Some interventions are more effective in certain subgroups of informal caregivers, so it is important to align the support to needs and characteristics of the caregivers.[38] In a review by Brodaty and Arasaratnam,[39] it was concluded that non pharmacological interventions taught to family caregivers could reduce the frequency and severity of behavioral and psychological symptoms of dementia as well as improve caregiver responses to these behaviors.

From previous meta-analyses and reviews, we know that intervention programs that focus on both the caregiver and person with dementia were often effective in delaying long-stay care admittance, but to a lesser extent in improving caregivers' mental health.[40] The most successful interventions used a psychoeducational or psychotherapeutic approach, addressed multiple stressors, were better adapted to the individual needs of the caregivers, and provided a higher amount and intensity of support.[41,42] A review by Pinquart and Sörensen[5] found that psychoeducational interventions have the widest-ranging effects, but only if they call for active participation of the informal caregiver. Incorporation of this kind of support into case management may improve outcomes in informal caregivers. Case managers are the most suitable health professionals to deliver these interventions because they are in close contact already with the informal caregivers and are trusted by them.

Conclusion

Our study found no differences between groups in primary outcomes. However, the analysis of the secondary outcomes indicated that there might be positive effects in the intensive case management as compared with the other 2 groups on quality of life, unmet, met, and total care needs. This warrants further research, as informal caregivers require more forms of psychological support than are currently available.

Supplementary Data

Supplementary data related to this article can be found online at http://dx.doi.org/10.1016/j.jamda.2015.06.011.

References

1. Prince M, Bryce R, Albanese E, et al. The global prevalence of dementia: A systematic review and metaanalysis. Alzheimers Dement 2013;9:63–75.e2.
2. Alzheimer's Association. 2013 Alzheimer's Disease Facts and Figures. Alzheimers Dement 2013;9:208–245.
3. Alzheimer Europe. Who Cares? The State of Dementia Care in Europe. Luxembourg: Alzheimer Europe; 2006.
4. Peeters JM, Van Beek AP, Meerveld JH, et al. Informal caregivers of persons with dementia, their use of and needs for specific professional support: A survey of the National Dementia Programme. BMC Nurs 2010;9:9.
5. Pinquart M, Sorensen S. Helping caregivers of persons with dementia: Which interventions work and how large are their effects? Int Psychogeriatr 2006;18: 577–595.
6. Pimouguet C, Lavaud T, Dartigues JF, Helmer C. Dementia case management effectiveness on health care costs and resource utilization: A systematic review of randomized controlled trials. J Nutr Health Aging 2010;14:669–676.
7. Schoenmakers B, Buntinx F, Delepeleire J. Factors determining the impact of care-giving on caregivers of elderly patients with dementia. A systematic literature review. Maturitas 2010;66:191–200.
8. John M. Eisenberg Center for Clinical Decisions and Communications Science. Effectiveness of Outpatient Case Management for Adults With Medical Illness and Complex Care Needs. Comparative Effectiveness Review Summary Guides for Clinicians. Rockville, MD: Agency for Healthcare Research and Quality; 2013.
9. Khanassov V, Vedel I, Pluye P. Case management for dementia in primary health care: A systematic mixed studies review based on the diffusion of innovation model. Clin Interv Aging 2014;9:915–928.
10. Case Management Society of America. What is a Case manager? 2015. Available at: http://www.cmsa.org/Home/CMSA/WhatisaCaseManager/tabid/224/Default.aspx. Accessed February 24, 2015.
11. MacNeil Vroomen J, Van Mierlo LD, van de Ven PM, et al. Comparing Dutch case management care models for people with dementia and their caregivers: The design of the COMPAS study. BMC Health Serv Res 2012;12:132.
12. Van Mierlo LD, Meiland FJ, Van Hout HP, Droes RM. Towards personalized integrated dementia care: A qualitative study into the implementation of different models of case management. BMC Geriatr 2014;14:84.
13. Banks P. Case management. In: Berman NPC, ed. Integrating Services for Older People—A Resource Book for Managers. Little Rock, AR, EHMA, 2004, p. 101–112.
14. Jansen AP, van Hout HP, van Marwijk HW, et al. (Cost)-effectiveness of case-management by district nurses among primary informal caregivers of older adults with dementia symptoms and the older adults who receive informal care: Design of a randomized controlled trial [ISCRTN83135728]. BMC Public Health 2005;5:133.
15. Cummings JL, Mega M, Gray K, et al. The Neuropsychiatric Inventory: Comprehensive assessment of psychopathology in dementia. Neurology 1994; 44:2308–2314.
16. Logsdon RG, Gibbons LE, McCurry SM, Teri L. Assessing quality of life in older adults with cognitive impairment. Psychosom Med 2002;64:510–519.
17. Reynolds T, Thornicroft G, Abas M, et al. Camberwell Assessment of Need for the Elderly (CANE). Development, validity and reliability. Br J Psychiatry 2000; 176:444–452.
18. Katz S, Downs TD, Cash HR, Grotz RC. Progress in development of the index of ADL. Gerontologist 1970;10:20–30.
19. Goldberg DP, Hillier VF. A scaled version of the General Health Questionnaire. Psychol Med 1979;9:139–145.
20. Brooks R. EuroQol: The current state of play. Health Policy 1996;37:53–72.
21. Pearlin LI, Schooler C. The structure of coping. J Health Soc Behav 1978;19: 2–21.
22. Vernooij-Dassen MJ, Felling AJ, Brummelkamp E, et al. Assessment of caregiver's competence in dealing with the burden of caregiving for a dementia patient: A Short Sense of Competence Questionnaire (SSCQ) suitable for clinical practice. J Am Geriatr Soc 1999;47:256–257.
23. De Jong Gierveld J, Kamphuis FH. The development of a Rasch-type loneliness-scale. Applied Psychological Measurement 1985;9:289–299.
24. Brouwer WB, van Exel NJ, van Gorp B, Redekop WK. The CarerQol instrument: A new instrument to measure care-related quality of life of informal caregivers for use in economic evaluations. Qual Life Res 2006;15:1005–1021.
25. Lamers LM, Stalmeier PF, McDonnell J, et al. [Measuring the quality of life in economic evaluations: The Dutch EQ-5D tariff]. Ned Tijdschr Geneeskd 2005; 149:1574–1578. Dutch.
26. McCaffrey DF, Griffin BA, Almirall D, et al. A tutorial on propensity score estimation for multiple treatments using generalized boosted models. Stat Med 2013;32:3388–3414.
27. R: A language and environment for statistical computing [computer program]; 2014. Vienna, Austria, R Foundation for Statistical Computing.
28. Goldberg DP, Gater R, Sartorius N, et al. The validity of two versions of the GHQ in the WHO study of mental illness in general health care. Psychol Med 1997; 27:191–197.
29. Goldberg DP, Oldehinkel T, Ormel J. Why GHQ threshold varies from one place to another. Psychol Med 1998;28:915–921.
30. Newcomer R, Yordi C, DuNah R, et al. Effects of the Medicare Alzheimer's Disease Demonstration on caregiver burden and depression. Health Serv Res 1999;34:669–689.
31. Callahan CM, Boustani MA, Unverzagt FW, et al. Effectiveness of collaborative care for older adults with Alzheimer disease in primary care: A randomized controlled trial. JAMA 2006;295:2148–2157.
32. Baugh RF, Freeman M. Ingredients of a successful case management program. Physician Exec 2003;29:30–33.
33. Huston CJ. The role of the case manager in a disease management program. Lippincotts Case Manag 2001;6:222–227.
34. Drennan V, Goodman C. Nurse-led case management for older people with long-term conditions. Br J Community Nurs 2004;9:527–533.
35. Somme D, Trouve H, Drame M, et al. Analysis of case management programs for patients with dementia: A systematic review. Alzheimers Dement 2012;8: 426–436.
36. Eska K, Graessel E, Donath C, et al. Predictors of institutionalization of dementia patients in mild and moderate stages: A 4-year prospective analysis. Dement Geriatr Cogn Dis Extra 2013;3:426–445.

37. Alzheimer's Disease International. World Alzheimer Report 2013. Journey of Caring: An Analysis of Long-Term Care for Dementia. London: Global Observatory for Ageing and Dementia Care, Health Service and Population Research Department, King's College London, 2013.
38. Van Mierlo LD, Meiland FJ, Van der Roest HG, Droes RM. Personalised caregiver support: Effectiveness of psychosocial interventions in subgroups of caregivers of people with dementia. Int J Geriatr Psychiatry 2012;27:1–14.
39. Brodaty H, Arasaratnam C. Meta-analysis of nonpharmacological interventions for neuropsychiatric symptoms of dementia. Am J Psychiatry 2012;169:946–953.
40. Smits CH, de Lange J, Droes RM, et al. Effects of combined intervention programmes for people with dementia living at home and their caregivers: A systematic review. Int J Geriatr Psychiatry 2007;22:1181–1193.
41. Brodaty H, Green A, Koschera A. Meta-analysis of psychosocial interventions for caregivers of people with dementia. J Am Geriatr Soc 2003;51:657–664.
42. Zarit S, Femia E. Behavioral and psychosocial interventions for family caregivers. Am J Nurs 2008;108:47–53. quiz 53.
