# Supplementary material for: The Cost-Effectiveness of Two Forms of Case Management Compared to a Control Group for Persons with Dementia and Their Informal Caregivers from a Societal Perspective
Source: PLoS One. 2016 Sep 21;11(9):e0160908. doi: 10.1371/journal.pone.0160908 (PMC5031395; doi:10.1371/journal.pone.0160908)
Supplement: S1 Case Record Form — Interview with informal caregiver (in Dutch). (DOC) [file pone.0160908.s003.doc]

## Interview mantelzorger

**T0**

**Inhoud**

Inleiding/registratie door interviewer

Inleidende vragen

Enkele vragen over uw naaste + schema

Kwaliteit van leven (EQ5D+C)

Gedragssymptomen (NPI)

Kwaliteit van leven van uw naaste (QoL AD)

Hoe uw naaste zich voelt (MH5)

Zorggebruik van uw naaste (MDS)

Medicijngebruik

Tijd besteed aan zorg (MDS)

Verzuim betaald werk

Zorgbehoefte (CANE)

Opmerkingen van de mantelzorger

Observaties van de interviewer

**Instructies**

*Veel instructies zullen in een box zoals deze staan. Verder is schuin gedrukte tekst alleen voor de interviewer bedoeld.*

**Inleiding voor de mantelzorger**

*Onderstaande tekst is slechts een voorbeeld en mag aangepast worden naar eigen inzicht.*

Fijn dat ik u mag interviewen voor de COMPAS studie. Het interview zal vandaag ongeveer een uur duren.

Ik zal u veel verschillende vragen stellen. Zo wil ik wat weten over de zorg die u en of uw naaste ontvangen, ik zal vragen stellen over hoe u zich voelt, gedragssymptomen van uw naaste, zorggebruik van uw naaste en nog meer.

Uw antwoorden worden gebruikt in onderzoek naar verbetering van de zorg voor ouderen. Uw ervaringen zijn dus waardevol, ook voor andere mensen met geheugenproblemen en hun mantelzorgers.

*Bij sommige patiënten is het onverstandig om ‘geheugenproblemen’ te noemen. Hou hier rekening mee als de patiënt in de buurt is en praat dan slechts over ouderen.*

Uw antwoorden worden anoniem verwerkt en uw gegevens worden niet verder verspreid.

Uw antwoorden op de vragen hebben geen invloed op de zorg die u en uw naaste ontvangen.

- Als u het moeilijk vindt om de vragen te begrijpen of te beantwoorden, vraagt u mij dan om hulp of uitleg.
- Sommige vragen lijken misschien dubbel maar het antwoord is toch heel waardevol. Ze zijn bedoeld om uw situatie nog eens van een andere kant te bekijken.

| **Registratie, in te vullen door interviewer** (MDS) |
| --- |

Studienummer: NL32949.029.10

Interviewer:

Respondentnummer (patiënt):

Geboortedatum mantelzorger:

Geboortedatum patiënt:

Datum afname:

Hoe afgenomen? (omcirkel)

1= a face‐to‐face interview

2= a telephone interview

3=a written/mailed questionnaire/ a webbased questionnaire (internet)

4=by a mailed questionnaire that was checked afterwards in a personal interview

5=by a mailed questionnaire that was checked afterwards in a telephone interview

| **Inleidende vragen** |
| --- |

Ik wil u graag eerst een paar vragen stellen over de zorg die u en uw naaste ontvangen in verband met de geheugenproblemen van uw naaste.

1. Ontvangen u en uw naaste momenteel begeleiding van een hulpverlener in verband met de geheugenproblemen van uw naaste?

*Meerdere antwoorden mogelijk, kies de best passende omschrijving. Als de mantelzorger antwoord: Geen begeleiding dan mag je opties A-H oplezen als voorbeeld.*

*Dit geldt ook voor vraag 2 ‘van welke organisatie ontvangen u en uw naaste deze begeleiding’ Onderaan het schema bij het sterretje staan voorbeelden.*

*Bij de vragen over hoe vaak het contact plaatsheeft gevonden in de afgelopen zes maanden kun je het totaal aantal bezoeken in de afgelopen zes maanden noteren, ook als de begeleiding nog geen zes maanden actief is. We rekenen dit later uit. (bijvoorbeeld sinds 3 maanden een case manager, deze is 3 maanden geleden en 1 maand geleden langs geweest dan noteer je bij vraag 4b: 2x*

***(Vul het schema op de volgende pagina in)***

| **1. Begeleider** | **2. Welke organisatie?*** | **3. Sinds wanneer (maand en jaar)** | **4a. Hoe vaak telefonisch contact per zes maanden** | **4b. Hoe vaak huisbezoek per zes maanden** | **4c. Hoe vaak bezoek aan hulpverlener per zes maanden** | **5a. Hoe lang duurt een telefonisch contact? (minuten)** | **5b. Hoe lang duurt een bezoek gemiddeld? (minuten)** |
| --- | --- | --- | --- | --- | --- | --- | --- |
| **A Verpleegkundige van de huisarts** |  |  |  |  |  |  |  |
| **B Verpleegkundige van de thuiszorg** |  |  |  |  |  |  |  |
| **C Verpleegkundige van de GGZ** |  |  |  |  |  |  |  |
| **D Maatschappelijk werker** |  |  |  |  |  |  |  |
| **E Welzijnswerker (bv Ouderenadviseur/ouderenconsulent)** |  |  |  |  |  |  |  |
| **F Medewerker ontmoetingscentrum** |  |  |  |  |  |  |  |
| **G Case manager/ trajectbegeleider / dementieconsulent/zorgcoördinator** |  |  |  |  |  |  |  |
| **H Anders, namelijk: …………………** |  |  |  |  |  |  |  |
| **I Geen van de bovenstaande (ga door naar vraag 7)** |  |  |  |  |  |  |  |

* bijvoorbeeld: Huisartspraktijk, Zorggroep Almere, Stichting Geriant, Welzijn ouderen, Thuiszorg:bv Cordaan, Osira, Icare, Buurtzorg, GGZ: bv Ingeest, Arkin, Symfora, Flevoland, Meerkanten, onbekend, overige namelijk:

6. Wat was de aanleiding voor deze begeleiding? Aangeven per begeleiding die genoemd is bij vraag (A-H).

(Vickrey)

7. Heeft een arts, verpleegkundige, maatschappelijk werker of andere zorgverlener in het afgelopen jaar samen met u een zorgplan opgesteld voor uw naaste met dementie?

□ Ja

□ Nee

8. Heeft u met een arts, verpleegkundige, maatschappelijk werker of andere zorgverlener samengewerkt om problemen voor het zorgplan te selecteren en manieren om hiermee om te gaan?

□ Ja

□ Nee

9. Bent u geadviseerd om, in de afgelopen 12 maanden een regelmatige, voorstelbare, vaste dagstructuur aan te brengen (zoals maaltijden, bedtijden, lichamelijke activiteit of beweging, etc) voor uw naaste met dementie?

□ Ja

□ Nee

10. Heeft een arts, verpleegkundige, maatschappelijk werker of andere zorgverlener gevraagd naar gedragssymptomen bij uw naaste en of deze gedragsproblemen door u als probleem worden ervaren?

□ Ja

□ Nee

11. Heeft een arts, verpleegkundige, maatschappelijk werker of andere zorgverlener met u en/of uw naaste overlegt over een wilsverklaring?

□ Ja

□ Nee

12. Heeft een arts, verpleegkundige, maatschappelijk werker of andere zorgverlener het met u gehad over mentors/curators? Is er vastgesteld/besloten wie mentor of curator is?

□ Ja, namelijk:……

□ Nee

13. Heeft een arts, verpleegkundige, maatschappelijk werker of andere zorgverlener met u gesproken over de beslisvaardigheid van uw naaste?

□ Ja

□ Nee

14. Heeft een arts, verpleegkundige, maatschappelijk werker of andere zorgverlener met u gesproken over de rijvaardigheid/rijstatus van uw naaste? Zijn hier afspraken over gemaakt?

□ Ja

□ Nee

(einde Vickrey)

| **Enkele vragen over uw naaste** |
| --- |

1. Hoe lang geleden zijn de symptomen van dementie bij uw naaste begonnen? (bijvoorbeeld verandering van gedrag of geheugen problemen)

|  |
| --- |

maanden geleden (probeer zo nauwkeurig mogelijk te schatten)

2. Is er een diagnose gesteld door een huisarts, geriater, neuroloog of andere arts?

□ Ja

□ Nee (ga naar vraag 5)

□ weet niet (ga naar vraag 5)

3. Wanneer is de diagnose gesteld? (beste schatting)

|  |
| --- |

Maand:

Jaar:

|  |
| --- |

□ Onbekend

4. Welk type dementie heeft uw naaste?

□ Ziekte van Alzheimer

□ Vasculaire dementie

□ Gemengde dementie (Alzheimer en vasculair)

□ Lewy body dementie

□ Frontotemporale dementie

□ Parkinson dementie

□ Onbekend

□ Weet niet

□ Anders, namelijk:

|  |
| --- |

5. Mogelijk heeft u een brief ontvangen over een zorgzwaartepakket van uw naaste. Indien u dit heeft ontvangen welk pakket stond hierin aangegeven?

*Pakketten en hun inhoudt mogen worden voorgelezen als voorbeeld*

□ Geen brief ontvangen over zorgzwaartepakket

□ Brief ontvangen maar weet niet welk zorgzwaartepakket

□ Pakket 1: Beschut wonen met enige begeleiding

□ Pakket 2: Beschut wonen met begeleiding en verzorging

□ Pakket 3: Beschut wonen met begeleiding en intensieve verzorging

□ Pakket 4: Beschut wonen met intensieve begeleiding en uitgebreide verzorging

□ Pakket 5: Beschermd wonen met intensieve dementiezorg

□ Pakket 6: Beschermd wonen met intensieve verzorging en verpleging

□ Pakket 7: Beschermd wonen met zeer intensieve zorg, vanwege specifieke aandoeningen, met de nadruk op begeleiding

□ Pakket 8: Beschermd wonen met zeer intensieve zorg, vanwege specifieke aandoeningen, met de nadruk op verzorging en verpleging

□ Pakket 9: Verblijf met herstelgerichte verpleging en verzorging

□ Pakket 10: Beschermd verblijf met intensieve palliatief-terminale zorg

| **Kwaliteit van leven** (MDS, EQ5D+C proxy-proxy) |
| --- |

| *Overhandig de deelnemer het formulier* |
| --- |

De volgende vragen gaan over hoe het vandaag met uw naaste gaat. Geef aan welke zin het beste past bij zijn/haar gezondheid zoals hij/zij die zelf ervaart.

Het gaat dus niet om uw indruk, maar om hoe u denkt dat uw naaste het ervaart.

1. Lopen:

□ Hij/zij heeft geen problemen met lopen

□ Hij/zij heeft enige problemen met lopen

□ Hij/zij is bedlegerig

2. Zelfzorg:

□ Hij/zij heeft geen problemen om zichzelf te wassen of aan te kleden

□ Hij/zij heeft enige problemen om zichzelf te wassen of aan te kleden

□ Hij/zij is niet in staat om zichzelf te wassen of aan te kleden

3. Dagelijkse activiteiten (bijvoorbeeld werk, studie, huishouden, gezins- en vrijetijdsactiviteiten):

□ Hij/zij heeft geen problemen met zijn/haar dagelijkse activiteiten.

□ Hij/zij heeft enige problemen met zijn/haar dagelijkse activiteiten.

□ Hij/zij is niet in staat zijn/haar dagelijkse activiteiten uit te voeren.

4. Pijn/klachten:

□ Hij/zij heeft geen pijn of andere klachten.

□ Hij/zij heeft matige pijn of andere klachten.

□ Hij/zij heeft zeer ernstige pijn of andere klachten.

5. Stemming:

□ Hij/zij is niet angstig of somber.

□ Hij/zij is matig angstig of somber.

□ Hij/zij is erg angstig of somber.

6. Hersenfuncties zoals geheugen, aandacht en denken:

□ Hij/zij heeft geen problemen met zijn/haar geheugen, aandacht en denken.

□ Hij/zij heeft problemen met zijn/haar geheugen, aandacht en denken.

□ Hij/zij heeft ernstige problemen met zijn/haar geheugen, aandacht en denken.

| **Gedragssymptomen** (NPI) |
| --- |

Overhandig de antwoordkaart, gebruik deze bij je uitleg

**Instructie**

*Onderstaande uitleg gebruiken om de vragen in te leiden:*

Deze vragen zijn bedoeld om het gedrag van uw naaste te beoordelen. U kunt de vragen over het algemeen beantwoorden met ja of nee, dus probeer zo beknopt mogelijk antwoord te geven.

*Stel alle vragen letterlijk. Als de persoon de vragen niet begrijpt kun je uitleg geven of verduidelijken. Je mag alleen synoniemen gebruiken. Let erop dat de vragen betrekking hebben op verandering in gedrag die zijn opgetreden sinds het begin van de ziekte. Gedragingen die daarvoor al aanwezig waren en die niet veranderd zijn gedurende de ziekte worden NIET gescoord, ook al zijn deze afwijkend. Gedrag dat altijd al aanwezig was maar dat veranderd is sinds de ziekte wordt wel gescoord. Het gaat om gedrag wat de afgelopen vier weken aanwezig is geweest. Als de patiënt bijvoorbeeld zes maanden geleden depressief was maar de afgelopen vier weken niet dan scoor je het gedrag als niet aanwezig.*

De vragen gaan om gedrag wat is ontstaan sinds uw naaste ziek is. Verder moet het gedrag de afgelopen vier weken aanwezig zijn geweest. Als het langer geleden is dan wordt dat nu niet meegenomen.

*Indien een screeningsvraag bevestigend wordt geantwoord controleer je dit door naar een voorbeeld te vragen. Als dit terug te vinden is in de subvragen stel je de vragen over ernst, frequentie en emotionele belasting. Voor frequentie zeg je:*

Ik wil nu graag weten hoe vaak (noem de meest problematisch ervaren gedragingen) is voorgekomen. Kwam het minder dan één keer per week voor, of ongeveer één keer per week, verschillende keren per week maar niet iedere dag, of iedere dag?

*Om ernst te bepalen zeg je:* Nu zou ik graag willen weten hoe ernstig dit gedraag is. Met ernstig bedoel ik, in welke mate verstoort of beperkte het hem/haar? Vindt u de ernst van het (het gedrag) licht, matig of ernstig?

*Vervolgens vraag je naar de emotionele belasting voor de mantelzorger. Deze scoort de mantelzorger zelf: van 0 tot 5.*

**A. WANEN**

Is hij/zij overtuigd van bepaalde gedachten, waarvan u weet dat ze niet waar zijn? Beweert hij/zij bijvoorbeeld dat andere mensen hem/haar kwaad willen doen of van hem/haar stelen. Heeft hij/zij gezegd dat familieleden **anderen** zijn dan ze zeggen te zijn of denkt hij/zij dat 't huis niet het huis is waar ze in wonen? Ik vraag u niet naar een beetje achterdochtig zijn, maar ik wil weten of hij/zij ervan **overtuigd** is dat deze zaken hem overkomen. Dus, of hij/zij denkt dat het **echt zo is**.

□ **Nee** (stel de volgende screeningsvraag)

□ **Ja** (vraag een voorbeeld en controleer dit met de subvragen)

□ **NVT** (leg uit waarom)

Subvragen (deze hoef je niet te stellen maar gebruik je ter controle, zet een kruisje bij het betreffende gedrag)

- Gelooft hij/zij dat er gevaar dreigt, dat anderen van plan zijn om hem/haar te kwetsen of pijn te doen?
- Denk hij/zij dat andere hem/haar bestelen?
- Denkt hij/zij dat zijn/haar partner vreemd gaat of ontrouw is?
- Denkt hij/zij dat er ongenode gasten in zijn/haar huis verblijven?
- Gelooft hij/zij dat zijn/haar partner of anderen niet zijn wie ze zeggen dat ze zijn?
- Gelooft hij/zij dat hij/zij niet in zijn/haar eigen huis woont?
- Denkt hij/zij dat familieleden hem/haar in de steek willen laten?
- Gelooft hij/zij dat personen van televisie of uit tijdschriften ook daadwerkelijk aanwezig zijn in huis? (Praat hij/zij met hen, of zoekt hij op een andere manier contact?)
- Gelooft hij/zij andere ongewone of vreemde zaken die ik hier nog niet genoemd heb?

Als de het voorbeeld overeenkomt met de subvragen, bepaal dan de frequentie en de ernst van de wanen.

Frequentie: 1. Soms - minder dan een keer per week

1. Regelmatig - ongeveer één keer per week
2. Vaak - meerdere malen per week, maar niet iedere dag
3. Heel vaak - één of meerdere keren per dag

Ernst: 1. Licht - wanen zijn aanwezig maar lijken onschuldig en benauwen, beangstigen patiënt niet of nauwelijks

1. Matig - patiënt raakt overstuur en ontregeld door de wanen
2. Ernstig - de wanen zijn zeer verstorend en vormen de belangrijkste bron van gedragsproblemen (indien neuroleptica zijn voorgeschreven, dan betekent dit dat de wanen ernstig tot zeer ernstig zijn)

Emotionele belasting: Hoe emotioneel belastend is dit gedrag voor u?

1. geen
2. minimaal
3. licht
4. matig
5. ernstig
6. zeer ernstig of extreem

**B. HALLUCINATIES**

Hallucineert hij/zij?; ziet of voelt hij/zij iets dat er niet is of hoort hij/zij stemmen die een ander niet kan horen? Ik bedoel met deze vraag niet vergissingen, zoals het idee hebben dat iemand nog leeft terwijl deze al overleden is. Ik vraag echter of hij/zij abnormale ervaringen heeft wat betreft iets horen, zien of voelen.

□ **Nee** (stel de volgende screeningsvraag)

□ **Ja** (vraag een voorbeeld en controleer dit met de subvragen)

□ **NVT** (leg uit waarom)

Subvragen (deze hoef je niet te stellen maar gebruik je ter controle, zet een kruisje bij het betreffende gedrag)

- Zegt hij/zij dat hij/zij stemmen hoort of gedraagt hij/zij zich alsof hij/zij stemmen hoort?
- Spreekt hij/zij tegen mensen die er niet zijn?
- Zegt hij/zij iets te zien dat een ander niet ziet of gedraagt hij/zij zich alsof hij/zij iets ziet wat een ander niet kan zien (zoals mensen, dieren, lichten, enz.)?
- Zegt hij/zij iets te ruiken wat een ander niet ruikt?
- Zegt hij/zij iets te voelen op zijn/haar huid? Of lijkt het alsof hij/zij iets voelt kruipen of kriebelen of dat iets hem/haar aanraakt?
- Zegt hij/zij iets te proeven zonder dat daar een duidelijke reden voor is?
- Heeft hij/zij u over andere ongewone gewaarwordingen verteld?.............................

Als de subvragen de screeningsvraag bevestigen, bepaal dan de frequentie en ernst van de hallucinaties.

Frequentie: 1. Soms - minder dan één keer per week

1. Regelmatig - ongeveer één keer per week
2. Vaak - verscheidene keren per week maar niet iedere dag
3. Heel vaak - één of meerdere keren per dag

Ernst: 1. Licht - hallucinaties zijn aanwezig maar onschuldig en beangstigen of benauwen patiënt niet of nauwelijks

1. Matig - patiënt raakt overstuur en ontregeld door de hallucinaties
2. Ernstig - de hallucinaties zijn zeer verstorend en vormen de belangrijkste bron van gedragsproblemen (neuroleptica kunnen geïndiceerd zijn om de hallucinaties te behandelen)

Emotionele belasting: Hoe emotioneel belastend is dit gedrag voor u?

1. geen
2. minimaal
3. licht
4. matig
5. ernstig
6. zeer ernstig of extreem

**C. AGITATIE/AGRESSIE**

Komt het voor dat hij/zij weigert mee te werken, of zich niet laat helpen door een ander? Is hij/zij lastig om mee om te gaan?

□ **Nee** (stel de volgende screeningsvraag)

□ **Ja** (vraag een voorbeeld en controleer dit met de subvragen)

□ **NVT** (leg uit waarom)

Subvragen (deze hoef je niet te stellen maar gebruik je ter controle, zet een kruisje bij het betreffende gedrag)

- Raakt hij/zij overstuur of wordt hij/zij kwaad op anderen die hem/haar willen verzorgen? Of verzet hij/zij zich bij het aankleden of wassen?
- Is hij/zij koppig of eigenwijs? Wil hij/zij dat alles moet gaan zoals hij/zij het wenst/
- Werkt hij/zij niet goed mee? Verzet hij/zij zich als anderen helpen?
- Zijn er andere gedragingen die het moeilijk maken om hem/haar te begeleiden?
- Vloekt of schreeuwt hij/zij kwaad?
- Slaat hij/zij met deuren, schopt hij/zij tegen meubilair of smijt hij/zij met voorwerpen?
- Probeert hij/zij anderen pijn te doen of anderen te slaan?
- Is hij/zij nog op een andere manier agressief of geagiteerd?

Als de subvragen de screeningsvraag bevestigen, bepaal dan de frequentie en de ernst van de agitatie.

Frequentie: 1. Soms - minder dan één keer per week

1. Regelmatig - ongeveer één keer per week
2. Vaak - verscheidene keren per week, maar niet iedere dag
3. Heel vaak - één of meerdere keren per dag

Ernst: 1. licht - het gedrag is verstorend maar is positief te beïnvloeden door afleiding of geruststelling

1. Matig - het gedrag is verstorend en moeilijk bij te sturen of te corrigeren
2. Ernstig - de agitatie is zeer verstorend en de belangrijkste bron van problemen;

het kan zijn dat de patiënt fysiek bedreigend is naar zijn omgeving. Vaak is medicatie geïndiceerd.

Emotionele belasting: Hoe emotioneel belastend is dit gedrag voor u?

1. geen
2. minimaal
3. licht
4. matig
5. ernstig
6. zeer ernstig of extreem

**D. DEPRESSIE/DYSFORIE**

Lijkt het alsof hij/zij verdrietig of depressief is? Zegt hij/zij dat hij/zij zich verdrietig of depressief voelt?

□ **Nee** (stel de volgende screeningsvraag)

□ **Ja** (vraag een voorbeeld en controleer dit met de subvragen)

□ **NVT** (leg uit waarom)

Subvragen (deze hoef je niet te stellen maar gebruik je ter controle, zet een kruisje bij het betreffende gedrag)

- Heeft hij/zij perioden dat hij/zij snel volschiet of veel huilt, wat er op wijst dat hij/zij verdrietig is?
- Doet of zegt hij/zij iets waaruit op te maken valt dat hij/zij verdrietig of down is?
- Haalt hij/zij zichzelf naar beneden of zegt hij/zij zichzelf niets waard te vinden, een mislukkeling te vinden?
- Zegt hij/zij dat hij/zij een slecht persoon is of straf verdient?
- Lijkt hij/zij erg ontmoedigd of zegt hij/zij dat de toekomst hem/haar niets te bieden heeft?
- Zegt hij/zij dat hij/zij anderen tot last is of dat familieleden beter af zouden zijn zonder hem/haar?
- Zegt hij/zij liever dood te willen zijn of zegt hij/zij zichzelf wat aan te willen doen, er een einde aan te willen maken?
- Zijn er andere signalen waaruit blijkt dat hij/zij verdrietig of depressief is?

Als de subvragen de screeningsvraag bevestigen, bepaal dan de frequentie en de ernst van de depressie.

Frequentie: 1. Soms - minder dan één keer per week.

1. Regelmatig - ongeveer één keer per week
2. Vaak - verscheidene keren per week maar niet iedere dag
3. Heel vaak - in essentie continu aanwezig

Ernst: 1.Licht - de depressie veroorzaakt leed, maar klaart meestal op bij afleiding of geruststelling

1. Matig - de depressie veroorzaakt leed, depressieve symptomen worden spontaan geuit door patiënt en deze zijn moeilijk te verlichten
2. Ernstig - de depressie veroorzaakt veel leed en vormt de belangrijkste bron van lijden voor de patiënt

Emotionele belasting: Hoe emotioneel belastend is dit gedrag voor u?

1. geen
2. minimaal
3. licht
4. matig
5. ernstig
6. zeer ernstig of extreem

**E. ANGST**

Is hij/zij erg nerveus, bezorgd, of schrikachtig zonder duidelijke reden? Lijkt hij/zij erg gespannen, rusteloos of zenuwachtig? Is hij/zij bang om alleen te zijn, zonder U?

□ **Nee** (stel de volgende screeningsvraag)

□ **Ja** (vraag een voorbeeld en controleer dit met de subvragen)

□ **NVT** (leg uit waarom)

Subvragen (deze hoef je niet te stellen maar gebruik je ter controle, zet een kruisje bij het betreffende gedrag)

- Zegt hij/zij dat hij/zij zich zorgen maakt over uitjes of activiteiten die gepland zijn?
- Voelt hij/zij zich wel eens trillerig, niet in staat zich te ontspannen of erg gespannen?
- Komt het voor dat hij/zij last heeft van kortademigheid, het moeten happen naar lucht, of zuchten (of klaagt hij/zij hierover), zonder duidelijke reden anders dan nervositeit?
- Klaagt hij/zij over kriebels in zijn/haar buik of over hartkloppingen die samengaan met nervositeit? (niet i.h.k.v. bijkomende ziekte)
- Vermijdt hij/zij bepaalde plaatsen of situaties die hem/haar nerveus maken, zoals autorijden, het ontmoeten van vrienden, of naar een plek gaan waar veel mensen zijn?
- Wordt hij/zij zenuwachtig of overstuur wanneer u (of verzorgende) weggaat? Houdt hij/zij zich dan krampachtig aan u vast om dat voorkomen?
- Is er nog iets anders dat erop wijst dat hij/zij angstig is?

Als de subvragen de screeningsvraag bevestigen, bepaal dan de ernst en de frequentie van de angst.

Frequentie: 1. Soms - minder dan één keer per week

1. Regelmatig - ongeveer één keer per week
2. Vaak - verscheidene keren per week maar niet iedere dag
3. Heel vaak - één of meerder keren per dag

Ernst: 1. Licht - de angst veroorzaakt leed, maar reageert meestal gunstig op afleiding of geruststelling

1. Matig - de angst veroorzaakt leed, angst symptomen worden spontaan geuit door patiënt en deze zijn moeilijk te verlichten
2. Ernstig - de angst veroorzaakt veel leed en vormt de belangrijkste bron van lijden voor de patiënt

Emotionele belasting: Hoe emotioneel belastend is dit gedrag voor u?

1. geen
2. minimaal
3. licht
4. matig
5. ernstig
6. zeer ernstig of extreem

**F. EUFORIE/OPGETOGENHEID**

Lijkt hij/zij te vrolijk, opgewekt of blij zonder duidelijke aanleiding? Ik bedoel niet de normale opgewektheid wanneer men vrienden ontmoet, een cadeau krijgt of tijd doorbrengt met familie. Ik vraag of hij/zij voortdurend een abnormaal goed humeur heeft of ergens om lacht waar een ander de humor niet van inziet.

□ **Nee** (stel de volgende screeningsvraag)

□ **Ja** (vraag een voorbeeld en controleer dit met de subvragen)

□ **NVT** (leg uit waarom)

Subvragen (deze hoef je niet te stellen maar gebruik je ter controle, zet een kruisje bij het betreffende gedrag)

- Lijkt hij/zij zich te goed of te opgewekt te voelen, vergeleken met hoe hij/zij normaal gesproken is?
- Vindt hij/zij iets grappig of lacht hij/zij ergens om waar een ander de humor niet van inziet?
- Lijkt het net alsof hij/zij een kinderachtig gevoel voor humor heeft en giechelt of ongepast lacht? (Bijvoorbeeld als iemand iets vervelends overkomt)
- Vertelt hij/zij moppen of plaatst hij/zij opmerkingen die hij/zij zelf wel grappig vindt, maar waar anderen niet om kunnen lachen?
- Haalt hij/zij kinderachtige streken uit, zoals verstoppertje spelen of iemand knijpen, gewoon voor de grap?
- Schept hij/zij op, bijvoorbeeld door te zeggen dat hij/zij rijk is of iets bijzonders kan, terwijl dat niet zo is?
- Is er nog iets anders waaruit blijkt dat hij/zij zich te goed of te opgewekt voelt?

Als de subvragen de screeningsvraag bevestigen, bepaal dan de frequentie en de ernst van de euforie.

Frequentie: 1. Soms - minder dan één keer per week

1. Regelmatig - ongeveer één keer per week
2. Vaak - verscheidene keren per week maar niet iedere dag
3. Heel vaak - in essentie constant aanwezig

Ernst: 1. Licht - uitgelatenheid valt op bij vrienden en familie maar is niet storend

1. Matig - de uitgelatenheid is duidelijk abnormaal
2. Ernstig - de uitgelatenheid is zeer opvallend; patiënt is eufoor en hij/zij vindt alles amusant en grappig

Emotionele belasting: Hoe emotioneel belastend is dit gedrag voor u?

1. geen
2. minimaal
3. licht
4. matig
5. ernstig
6. zeer ernstig of extreem

**G. APATHIE/ONVERSCHILLIGHEID**

Is hij/zij niet langer geïnteresseerd in de wereld om hem/haar heen? Heeft hij/zij geen belangstelling meer om iets te doen of ontbreekt de motivatie om aan iets nieuws te beginnen? Is hij/zij moeilijker te betrekken in een gesprek of het doen van huishoudelijke klusjes? Is hij/zij apathisch of onverschillig?

□ **Nee** (stel de volgende screeningsvraag)

□ **Ja** (vraag een voorbeeld en controleer dit met de subvragen)

□ **NVT** (leg uit waarom)

Subvragen (deze hoef je niet te stellen maar gebruik je ter controle, zet een kruisje bij het betreffende gedrag)

- Lijkt hij/zij minder spontaan en minder actief dan gewoonlijk?
- Begint hij/zij minder vaak een gesprek?
- Toont hij/zij minder affectie of misschien wel helemaal geen emoties, vergeleken met hoe hij/zij normaal gesproken was?
- Helpt hij/zij minder met huishoudelijke klussen?
- Lijkt hij/zij minder geïnteresseerd in de activiteiten en plannen van een ander?
- Heeft hij/zij geen belangstelling meer voor vrienden of familie?
- Is hij/zij minder enthousiast over zaken die hem gewoonlijk interesseren?
- Is er nog iets anders waaruit blijkt dat hij/zij geen interesse heeft om iets nieuws te gaan doen?

Als de subvragen de screeningsvraag bevestigen, bepaal dan de ernst en de frequentie van de apathie.

Frequentie: 1. Soms - minder dan één keer per week

1. Regelmatig - ongeveer één keer per week
2. Vaak - verscheidene keren per week maar niet iedere dag
3. Heel vaak - in essentie constant aanwezig

Ernst: 1. Licht – apathie valt op maar interfereert niet met de dagelijkse bezigheden; is slechts een beetje anders dan het gewone gedrag van de patiënt; hij/zij reageert op uitnodigingen om deel te nemen aan activiteiten.

2. Matig – apathie is evident; verzorgende kan ermee omgaan door op de patiënt in te praten of aan te moedigen; slechts indringende gebeurtenissen leiden tot een spontane actie, zoals het bezoek van naaste familieleden.

3. Ernstig – de apathie is evident en reageert niet langer op aanmoedigingen of gebeurtenissen in de omgeving.

Emotionele belasting: Hoe emotioneel belastend is dit gedrag voor u?

1. geen
2. minimaal
3. licht
4. matig
5. ernstig
6. zeer ernstig of extreem

**H. ONTREMD GEDRAG**

Lijkt hij/zij impulsief te handelen, zonder er bij na te denken? Doet of zegt hij/zij iets wat men normaal gesproken niet in het openbaar zegt of doet? Brengt hij/zij u of iemand anders in verlegenheid met wat hij/zij doet?

□ **Nee** (stel de volgende screeningsvraag)

□ **Ja** (vraag een voorbeeld en controleer dit met de subvragen)

□ **NVT** (leg uit waarom)

Subvragen (deze hoef je niet te stellen maar gebruik je ter controle, zet een kruisje bij het betreffende gedrag)

- Handelt hij/zij impulsief zonder over de gevolgen na te denken?
- Praat hij/zij tegen volstrekt onbekenden alsof hij/zij ze goed kent?
- Maakt hij/zij kwetsende of tactloze opmerkingen tegen anderen?
- Maakt hij/zij botte of seksueel getinte opmerkingen, die hij/zij normaal gesproken nooit gezegd zou hebben?
- Praat hij/zij openlijk over zeer persoonlijke of privé zaken, waarover men gewoonlijk niet spreekt in het openbaar?
- Gaat hij/zij te ver of is hij/zij handtastelijk of knuffelt hij/zij anderen, op zo’n manier dat het niet past bij zijn/haar karakter?
- Is er nog iets anders dat er op wijst dat hij/zij zijn/haar impulsen minder onder controle heeft?

Als de subvragen de screeningsvraag bevestigen, bepaal dan de frequentie en de ernst van het ontremde gedrag

Frequentie: 1. Soms - minder dan één keer per week

1. Regelmatig - ongeveer één keer per week
2. Vaak - verscheidene keren per week maar niet iedere dag
3. Heel vaak - in essentie continu aanwezig

Ernst: 1. Licht - ontremd gedrag is aanwezig, maar reageert op afleiding en begeleiding

1. Matig - ontremd gedrag is duidelijk aanwezig en moeilijk voor de verzorgende om mee om te gaan.
2. Ernstig - ontremd gedrag neemt niet af, wat de verzorgende ook probeert, het brengt anderen in verlegenheid of is een bron van ergernis

Emotionele belasting: Hoe emotioneel belastend is dit gedrag voor u?

1. geen
2. minimaal
3. licht
4. matig
5. ernstig
6. zeer ernstig of extreem

**I. PRIKKELBAARHEID/LABILITEIT**

Is hij/zij snel geïrriteerd of uit evenwicht? Is zijn/haar stemming nogal veranderlijk? Is hij/zij erg ongeduldig? Ik bedoel niet dat hij/zij zich gefrustreerd voelt over de vergeetachtigheid of alledaagse taken die niet meer lukken. Ik wil graag weten of hij/zij erg prikkelbaar en ongeduldig is of dat hij/zij snel van humeur verandert, anders dan u van hem/haar gewend bent?

□ **Nee** (stel de volgende screeningsvraag)

□ **Ja** (vraag een voorbeeld en controleer dit met de subvragen)

□ **NVT** (leg uit waarom)

Subvragen (deze hoef je niet te stellen maar gebruik je ter controle, zet een kruisje bij het betreffende gedrag)

- Is hij/zij opvliegend, schiet hij/zij gemakkelijk uit z’n slof om iets kleins?
- Verandert hij/zij snel van stemming, het ene moment is alles nog goed en het andere moment is hij/zij kwaad?
- Heeft hij/zij plotselinge woede uitbarstingen?
- Is hij/zij ongeduldig? Kan hij/zij moeilijk omgaan met een vertraging of het moeten wachten op een activiteit die gepland is?
- Is hij/zij humeurig of snel geïrriteerd?
- Gaat hij/zij snel in discussie en is het moeilijk met hem/haar op te schieten?
- Is er nog iets anders wat er op wijst dat hij/zij prikkelbaar is?

Als de subvragen de screeningsvraag bevestigen, bepaal dan de frequentie en de ernst van de prikkelbaarheid/labiliteit.

Frequentie: 1. Soms - minder dan één keer per week

1. Regelmatig - ongeveer één keer per week
2. Vaak - verscheidene keren per week maar niet iedere dag
3. Heel vaak - in essentie continu aanwezig

Ernst: 1. Licht - prikkelbaarheid wordt opgemerkt, maar is d.m.v. geruststelling

of afleiding te corrigeren

1. Matig - prikkelbaarheid is evident aanwezig en moeilijk mee om te gaan voor de verzorgende
2. Ernstig - prikkelbaarheid is evident aanwezig, is niet bij te sturen door de verzorgende en is zeer belastend

Emotionele belasting: Hoe emotioneel belastend is dit gedrag voor u?

1. geen
2. minimaal
3. licht
4. matig
5. ernstig
6. zeer ernstig of extreem

**J. DOELLOOS REPETITIEF GEDRAG**

Loopt hij/zij doelloos rond, te ijsberen? Doet hij/zij een handeling telkens weer, zoals keer op keer een la opentrekken, aan iets zitten te plukken of touwtjes of draadjes opwinden?

□ **Nee** (stel de volgende screeningsvraag)

□ **Ja** (vraag een voorbeeld en controleer dit met de subvragen)

□ **NVT** (leg uit waarom)

Subvragen (deze hoef je niet te stellen maar gebruik je ter controle, zet een kruisje bij het betreffende gedrag)

- Loopt hij/zij doelloos rond in huis?
- Loopt hij/zij rond te zoeken en te rommelen met spullen, zoals het openen van laden en het leeghalen van kasten?
- Kleedt hij/zij zich herhaaldelijk aan en dan weer uit?
- Zijn er gewoonten of activiteiten die hij/zij constant herhaalt?
- Doet hij/zij telkens dezelfde handeling zoals peuteren aan knopen, ergens aan plukken, draadjes opwinden etc?
- Beweegt hij/zij zenuwachtig, alsof hij/zij niet stil kan blijven zitten, of schuift hij/zij met de voeten heen en weer of trommelt hij/zij vaak met de vingers?
- Zijn er andere activiteiten die hij/zij telkens weer herhaalt?

Als de subvragen de screeningsvraag bevestigen, bepaal dan de frequentie en de ernst van het doelloos repetitieve gedrag

Frequentie: 1. Soms - minder dan één keer per week

1. Regelmatig - ongeveer één keer per week
2. Vaak - verscheidene keren per week maar niet iedere dag
3. Heel vaak - in essentie continu aanwezig

Ernst: 1. Licht - doelloos repetitief gedrag wordt opgemerkt, maar interfereert

nauwelijks met de dagelijkse bezigheden

1. Matig - doelloos repetitief gedrag is duidelijk aanwezig, maar de verzorgende weet er mee om te gaan

3. Ernstig - doelloos repetitief gedrag is duidelijk aanwezig, is nauwelijks te

corrigeren door de verzorgende en vormt een grote belasting

Emotionele belasting: Hoe emotioneel belastend is dit gedrag voor u?

1. geen
2. minimaal
3. licht
4. matig
5. ernstig
6. zeer ernstig of extreem

**K. NACHTELIJKE ONRUST/SLAAPSTOORNIS**

Heeft hij/zij moeite met slapen? (scoor als 'niet aanwezig' indien patiënt slechts een of twee keer per nacht opstaat om naar het toilet te gaan en daarna weer meteen in slaap valt). Is hij/zij 's nachts op? Loopt hij/zij 's nachts rond, kleedt hij/zij zich aan of verstoort hij/zij uw nachtrust?

□ **Nee** (stel de volgende screeningsvraag)

□ **Ja** (vraag een voorbeeld en controleer dit met de subvragen)

□ **NVT** (leg uit waarom)

Subvragen (deze hoef je niet te stellen maar gebruik je ter controle, zet een kruisje bij het betreffende gedrag)

- Heeft hij/zij moeite met in slaap vallen?
- Staat hij/zij 's nachts op? (scoor als afwezig indien patiënt een of twee keer opstaat om naar het toilet te gaan en snel weer inslaapt)
- Loopt hij/zij 's nachts doelloos rond, te ijsberen of is hij/zij dan bezig met ongebruikelijke activiteiten?
- Maakt hij/zij u s’nachts wakker?
- Wordt hij/zij 's nachts wakker en kleedt hij/zij zich dan aan in de veronderstelling dat het al morgen is en tijd om aan een nieuwe dag te beginnen?
- Wordt hij/zij 's ochtends te vroeg wakker (vroeger dan hij/zij gewend was)?
- Slaapt hij/zij buitensporig veel overdag?
- Is hij/zij 's nachts nog met iets anders bezig dat u hindert, waar we het nog niet over gehad hebben?

Als de subvragen de screeningsvraag bevestigen, bepaal dan de ernst en de frequentie van de slaapproblemen en nachtelijke onrust

Frequentie: 1. Soms - minder dan één keer per week

1. Regelmatig - ongeveer één keer per week
2. Vaak - verscheidene keren per week maar niet iedere dag
3. Heel vaak - in essentie continu aanwezig

Ernst: 1. Licht - nachtelijke onrust komt voor maar is niet echt hinderlijk

2. Matig - nachtelijke onrust komt voor, is storend voor de patiënt en voor de nachtrust van de verzorgende. Meerdere gedragingen kunnen zich per nacht voordoen.

1. Ernstig - nachtelijke onrust komt voor; er kunnen meerdere gedragingen voorkomen; patiënt is zeer ontdaan en verstoort in ernstige mate de nachtrust van de verzorgende

Emotionele belasting: Hoe emotioneel belastend is dit gedrag voor u?

1. geen
2. minimaal
3. licht
4. matig
5. ernstig
6. zeer ernstig of extreem

**L. EETLUST/EETGEDRAG VERANDERING**

Is hij/zij veranderd qua eetlust, gewicht of eetgewoonten (beoordeel NVT als de patiënt niet in staat is om zelf te eten en gevoed moet worden)? Is er verandering gekomen in het soort eten dat hij/zij lekker vindt?

□ **Nee** (stel de volgende screeningsvraag)

□ **Ja** (vraag een voorbeeld en controleer dit met de subvragen)

□ **NVT** (leg uit waarom)

Subvragen (deze hoef je niet te stellen maar gebruik je ter controle, zet een kruisje bij het betreffende gedrag)

- Is zijn/haar eetlust verminderd?
- Is zijn/haar eetlust toegenomen?
- Is hij/zij afgevallen?
- Is hij/zij aangekomen in gewicht?
- Is zijn/haar eetgedrag veranderd, zoals te veel eten tegelijkertijd in de mond stoppen?
- Is zijn/haar voorkeur voor bepaald eten veranderd? Bijvoorbeeld heeft hij/zij veel meer trek gekregen in zoetigheid of iets anders?
- Heeft hij/zij bepaald eetgedrag ontwikkeld, zoals elke dag precies hetzelfde willen eten of alles in dezelfde volgorde willen opeten?
- Zijn er andere veranderingen in de eetlust en het eetgedrag die ik hier nog niet genoemd heb?

Als de subvragen de screeningsvraag bevestigen, bepaal dan de ernst en de frequentie van de veranderingen in eetgedrag en eetlust.

Frequentie: 1. Soms - minder dan één keer per week

1. Regelmatig - ongeveer één keer per week
2. Vaak - verscheidene keren per week maar niet iedere dag
3. Heel vaak - in essentie continu aanwezig

Ernst: 1. Licht - veranderingen in eetlust en eetgewoonten zijn aanwezig, maar hebben niet geleid tot een verandering in het gewicht en zijn niet storend.

2. Matig - veranderingen in eetlust en eetgewoonten zijn aanwezig en leiden tot

kleine toe- of afname in lichaamsgewicht

3. Ernstig er zijn duidelijke veranderingen in eetlust en eetgewoonte aanwezig

die leiden tot toe- of afname in lichaamsgewicht, gênant zijn, of anderszins

storend zijn voor patiënt

Emotionele belasting: Hoe emotioneel belastend is dit gedrag voor u?

1. geen
2. minimaal
3. licht
4. matig
5. ernstig

5 zeer ernstig of extreem

| **kwaliteit van leven van uw naaste** (Qol-AD) |
| --- |

*Overhandig de deelnemer het formulier. Hij of zij mag de juiste antwoorden omcirkelen. Lukt dat niet dan mag het juiste antwoord aangewezen worden of worden uitgesproken.*

De volgende vragen gaan over de kwaliteit van leven van uw naaste.

Als u denkt over het leven van uw naaste, dan zijn er verschillende gebieden, waarvan sommige hieronder staan weergegeven. Wilt u over elke vraag nadenken en aangeven hoe de huidige kwaliteit van leven van uw naaste is voor elk gebied. Maak daarbij gebruik van één van de vier antwoordmogelijkheden: slecht, redelijk, goed, of uitstekend. Wilt u deze vragen beantwoorden voor de kwaliteit van leven van uw naaste op dit moment (in de afgelopen paar weken). Als er onduidelijkheden zijn over een vraag, kunt u om uitleg vragen.

*Het gaat dan OVER de patiënt. De mantelzorger mag antwoorden omcirkelen, aanwijzen of noemen.*

1. Ten eerste, wat vindt u van de lichamelijke gezondheid van uw naaste? Zou u zeggen dat die slecht, redelijk, goed of uitstekend is? Omcirkel nu welk woord u denkt dat het beste de lichamelijke gezondheid van uw naaste omschrijft.

Slecht Redelijk Goed Uitstekend

1. Wat is uw mening over de vitaliteit van uw naaste? Vindt u dat deze slecht, redelijk, goed of uitstekend is? Als de deelnemer zegt dat sommige dagen beter zijn dan andere, vraag hem/haar te bepalen hoe zijn/haar naaste zich meestal voelde de laatste tijd.

Slecht Redelijk Goed Uitstekend

1. Hoe was de stemming van uw naaste de laatste tijd? Voelde hij/zij zich prettig, of heeft hij/zij zich somber gevoeld? Zou u de stemming van uw naaste als slecht, redelijk, goed of uitstekend beoordelen?

Slecht Redelijk Goed Uitstekend

1. Wat vindt u van de woonomstandigheden van uw naaste? Wat vindt u van de plek waar uw naaste nu woont? Zou u zeggen dat deze slecht, redelijk, goed of uitstekend is?

Slecht Redelijk Goed Uitstekend

1. Wat vindt u van het geheugen van uw naaste? Vindt u dat dit slecht, redelijk, goed of uitstekend is?

Slecht Redelijk Goed Uitstekend

1. Wat vindt u van de familie en de relatie met de familieleden van uw naaste? Zou u dit omschrijven als slecht, redelijk, goed of uitstekend? Als de deelnemer zegt dat zijn/haar naaste geen familie (meer) heeft, vraag dan naar broers, zussen, kinderen, neven.

Slecht Redelijk Goed Uitstekend

1. Wat vindt u van het huwelijk van uw naaste? Hoe is de relatie met (naam partner) van uw naaste? Vindt u dat die slecht, redelijk, goed of uitstekend is? Sommige deelnemers zullen alleenstaand, weduwnaar/weduwe of gescheiden zijn. Wanneer dat het geval is, vraag wat zij vinden van de persoon met wie hun naaste de meest intieme relatie heeft, of dat nu een familielid is of niet. Als er een familie ‘caregiver’ is, vraag naar de relatie met deze persoon. Als er niemand in aanmerking komt, of de deelnemer is onzeker, scoor dit onderdeel dan als ‘missing’.

Slecht Redelijk Goed Uitstekend

1. Hoe zou u de relatie van uw naaste met zijn/haar vrienden omschrijven? Vindt u die slecht, redelijk goed of uitstekend? Als de deelnemer antwoordt dat de naaste geen vrienden heeft, of al zijn/haar vrienden overleden zijn, probeer verder. Heeft uw naaste iemand wiens gezelschap hij/zij op prijs stelt, naast zijn/haar familie? Zou hij/zij die persoon een vriend noemen? Als de deelnemer nog steeds zegt dat de geen vrienden heeft, vraag dan: Hoe voelt dat voor uw naaste om geen vrienden te hebben: slecht, redelijk, goed of uitstekend?

Slecht Redelijk Goed Uitstekend

1. Wat vindt u van uw naaste? Wanneer u denkt aan zijn/haar gehele persoon, en al de verschillende aspecten over hem/haarzelf, zou u zeggen dat u dat slecht, redelijk, goed of uitstekend vindt?

Slecht Redelijk Goed Uitstekend

1. Wat vindt u van het vermogen van uw naaste om dingen als karweitjes rond het huis te doen of andere dingen die hij/zij moet doen? Zou u zeggen dat, dat slecht, redelijk, goed of uitstekend is?

Slecht Redelijk Goed Uitstekend

1. Wat vindt u van het vermogen van uw naaste om leuke dingen te doen, waar hij/zij plezier aan beleeft? Vindt u dat slecht, redelijk, goed of uitstekend?

Slecht Redelijk Goed Uitstekend

1. Wat vindt u van de huidige situatie van uw naaste wat betreft geld, zijn/haar financiële situatie? Vindt u deze slecht, redelijk, goed of uitstekend? Als de deelnemer aarzelt, leg dan uit dat je niet wilt weten wat de situatie is (wat betreft hoeveelheid geld), maar dat je alleen wilt weten wat hij/zij vindt van de situatie van zijn/haar naaste.

Slecht Redelijk Goed Uitstekend

1. Hoe zou u het leven van uw naaste over het geheel omschrijven? Wanneer u nadenkt over zijn/haar leven als geheel, alles bij elkaar genomen, hoe denkt u dan over zijn/haar leven? Vindt u dat slecht, redelijk, goed of uitstekend?

Slecht Redelijk Goed Uitstekend

| **Hoe uw naaste zich voelt** (MDS, psychisch welbevinden MH5) |
| --- |

De volgende vragen gaan over hoe uw naaste zich de afgelopen maand heeft gevoeld. Kies het antwoord dat het beste bij uw naaste past.

| *Overhandig de deelnemer het formulier* |
| --- |

1. Hoe vaak is uw naaste in de afgelopen maand erg nerveus geweest?

□ Altijd

□ Heel vaak

□ Redelijk vaak

□ Soms

□ Bijna nooit

□ Nooit

2. Hoe vaak heeft uw naaste zich de afgelopen maand kalm en rustig gevoeld?

□ Altijd

□ Heel vaak

□ Redelijk vaak

□ Soms

□ Bijna nooit

□ Nooit

3. Hoe vaak heeft uw naaste zich de afgelopen maand neerslachtig en somber gevoeld?

□ Altijd

□ Heel vaak

□ Redelijk vaak

□ Soms

□ Bijna nooit

□ Nooit

4. Hoe vaak heeft uw naaste zich de afgelopen maand gelukkig gevoeld?

□ Altijd

□ Heel vaak

□ Redelijk vaak

□ Soms

□ Bijna nooit

□ Nooit

5. Hoe vaak heeft uw naaste zich de afgelopen maand zo somber gevoeld dat niets hem of haar kon opvrolijken?

□ Altijd

□ Heel vaak

□ Redelijk vaak

□ Soms

□ Bijna nooit

□ Nooit

| **Zorggebruik van uw naaste** (MDS, zorggebruik) |
| --- |

De volgende vragen gaan over hoeveel zorg uw naaste het afgelopen jaar heeft gebuikt bij professionele instellingen. Het kan lastig zijn om zover terug te denken. Probeert u alstublieft zo goed mogelijk weer te geven welke zorg er is gebruikt.

1. Is uw naaste de afgelopen 12 maanden opgenomen geweest in een ziekenhuis?

□ Nee

|  |
| --- |

□ Ja, namelijk dagen in totaal.

Zo ja, in welke ziekenhuizen?

Opname 1

Ziekenhuis:

Plaats:

Opname 2

Ziekenhuis:

Plaats:

Opname 3

Ziekenhuis:

Plaats:

Opname 4

Ziekenhuis:

Plaats:

Opname 5

Ziekenhuis:

Plaats:

2. Heeft uw naaste in de afgelopen 12 maanden de huisartsenpost bezocht of een visite van een huisarts gehad in avond, nacht of weekend?

□ Nee

□ Ja, namelijk keer in totaal.

|  |
| --- |

3. Heeft uw naaste thuiszorg? Bijvoorbeeld wijkverpleging, gezinsverzorging of alfahulp.

□ Nee

|  |
| --- |

□ Ja, namelijk uur per week

4. Is uw naaste in de afgelopen 12 maanden tijdelijk opgenomen geweest in een verzorgingshuis? Bijvoorbeeld omdat hij of zij na een ziekenhuisopname nog niet direct naar huis kon.

□ Nee

□ Ja, namelijk weken in totaal

|  |
| --- |

5. Is uw naaste in de afgelopen 12 maanden tijdelijk opgenomen geweest in een verpleeghuis? Bijvoorbeeld omdat hij of zij na een ziekenhuisopname nog niet direct naar huis kon.

□ Nee

□ Ja, namelijk weken in totaal

|  |
| --- |

6. Gaat uw naaste naar dagopvang?

□ Nee

□ Ja, namelijk dagen per week

|  |
| --- |

7. Gaat uw naaste naar dagbehandeling?

□ Nee

□ Ja, namelijk dagen per week

|  |
| --- |

| **Medicijngebruik** |
| --- |

| medicijnnaam | Concentratie mg, ml etc. | Aantal pillen, druppels, inhalaties etc. | Kies aantal per dag, week of maand |
| --- | --- | --- | --- |
|  |  |  | ….x per dag  ….x per week  ….x per maand |
|  |  |  | ….x per dag  ….x per week  ….x per maand |
|  |  |  | ….x per dag  ….x per week  ….x per maand |
|  |  |  | ….x per dag  ….x per week  ….x per maand |
|  |  |  | ….x per dag  ….x per week  ….x per maand |
|  |  |  | ….x per dag  ….x per week  ….x per maand |
|  |  |  | ….x per dag  ….x per week  ….x per maand |
|  |  |  | ….x per dag  ….x per week  ….x per maand |

| medicijnnaam | Concentratie mg, ml etc. | Aantal pillen, druppels, inhalaties etc. | Kies aantal per dag, week of maand |
| --- | --- | --- | --- |
|  |  |  | ….x per dag  ….x per week  ….x per maand |
|  |  |  | ….x per dag  ….x per week  ….x per maand |

1. Zitten hier medicijnen bij tegen depressie of gedragsproblemen? (Vickrey)

□ Nee (ga verder met de volgende vragenlijst: tijd besteed aan zorg voor uw naaste)

□ Ja 🡪 Hoe goed denkt u dat u de mogelijke voordelen, bijwerkingen en redenen waarom uw naaste deze medicatie gebruikt begrijpt?

□ Ik begrijp de voordelen, bijwerkingen en redenen goed.

□ Ik begrijp de voordelen, bijwerkingen en redenen enigszins maar heb nog wel vragen.

□ Ik begrijp de voordelen, bijwerkingen en redenen niet.

| **Tijd besteed aan zorg voor uw naaste** (MDS, iBMG) |
| --- |

*Let bij deze vragen goed op dat de afgelopen week in gedachte wordt genomen (indien dat een gemiddelde week was). Het is dus niet de bedoeling dat de mantelzorger over het geheel genomen gaat kijken hoeveel uur per week hij of zij bezig is met onderstaande taken.*

De volgende vragen gaan over de hoeveelheid tijd die u aan zorg voor uw naaste besteedt. U wordt gevraagd met welke verschillende bezigheden in het dagelijks leven u uw naaste heeft geholpen. Met ‘geholpen’ wordt bedoeld dat u uw naaste in de taak ondersteund hebt of dat u de taak overgenomen hebt. Probeer van alle bezigheden zo precies mogelijk in te schatten hoeveel tijd u eraan besteed hebt.

Sommige mantelzorgers hebben het gevoel de hele dag met de zorg voor hun naaste bezig te zijn. Bijvoorbeeld omdat de zorg heel zwaar is. Probeer ook dan zo precies mogelijk aan te geven hoeveel tijd u in werkelijkheid aan de zorg voor uw naaste besteedt.

Vul de vragen in zoals het de afgelopen week geweest is. Kies een andere week, als uw tijdsbesteding in de afgelopen week door bijvoorbeeld ziekte of vakantie heel anders was dan in een gemiddelde week.

1. Heeft u (naam) in de afgelopen week door zijn/haar gezondheidsproblemen moeten helpen bij huishoudelijke taken zoals klaarmaken van eten en drinken, schoonmaken van het huis, wassen, strijken en naaien van kleding, boodschappen doen of kleine klussen in huis of in de tuin?

□ Nee

□ Ja, namelijk uur per week

|  |
| --- |

2. Heeft u (naam) in de afgelopen week door zijn/haar gezondheidsproblemen moeten helpen bij persoonlijke verzorging (aan- en uitkleden, wassen, kammen, scheren), naar het toilet gaan, verplaatsen binnenshuis, eten, drinken of toedienen medicatie?

□ Nee

|  |
| --- |

□ Ja, namelijk uur per week

3. Heeft u (naam) in de afgelopen week door zijn/haar gezondheidsproblemen moeten helpen bij verplaatsen buitenshuis, maken van uitstapjes en bezoekjes aan familie of vrienden, contacten met de gezondheidszorg (mee naar bijvoorbeeld de huisarts, het ziekenhuis, een therapie), het regelen van hulp, hulpmiddelen en/of woningaanpassingen, en het regelen van financiële en administratieve zaken?

□ Nee

□ Ja, namelijk uur per week

|  |
| --- |

4. Krijgt (naam) behalve van u ook hulp van andere mantelzorger of vrijwilligers?

□ Nee

|  |
| --- |

□ Ja, namelijk uur per week

| **Verzuim betaald (en onbetaald) werk door zorg voor uw naaste en ziekte van uzelf** |
| --- |

1. Heeft u betaald werk?

□ ja

□ nee

Zo ja, hoeveel uur per week werkt u? ……… uren per week

En over hoeveel dagen zijn deze uren verspreid? …….. dagen

**Betaald werk**

|  | Aantal dagen |
| --- | --- |
| 2. Hoeveel dagen heeft u door ziekte niet kunnen werken? (in het afgelopen half jaar?) |  |
| 3. Hoeveel dagen heeft u door zorg voor uw naaste niet kunnen werken? (in het afgelopen half jaar?) |  |

**Onbetaalde bezigheden**

4. Hoeveel **uren** heeft u uw normale werkzaamheden **niet** kunnen doen door ziekte en zorg voor uw naaste? (in het afgelopen half jaar)

|  | Aantal uren (per week) |
| --- | --- |
| a. Huishoudelijk werk, klussen in huis |  |
| b. Vrijwilligerswerk |  |
| c. Studie/scholing |  |
| d. Andere dagelijkse bezigheden |  |

**Overname bezigheden vanwege gezondheidsklachten en zorg voor uw naaste**

5. Wie heeft bezigheden van u overgenomen in verband met gezondheidsklachten en zorg voor uw naaste?

|  | Aantal uren (per week) | Indien betaalt, totale kosten hiervoor |
| --- | --- | --- |
| a. Hulp van familie, vrienden, vrijwilligers |  | € |
| b. Betaalde hulp in de huishouding (bijvoorbeeld voor schoonmaakwerk/klussen) |  | € |
| c. Andere hulp |  | € |

| **Zorgbehoefte** (CANE) |
| --- |

**Instructie**

*De CANE is ingekort. Alle verdiepende vragen hoeven niet meer gesteld te worden. Je begint met de* **dikgedrukte** *vraag (of een eigen versie hiervan) te stellen. Achter deze vraag staat tussen haakjes (ontvang uw naaste hulp van vrienden, verwanten of plaatselijke voorzieningen bij het …?) Dit mag je als verdiepende vraag stellen wanneer je twijfelt over het antwoord. Je bepaalt zelf aan de hand van het antwoord of er geen behoefte, tegemoetgekomen behoefte, bestaande behoefte of onbekend gescoord moet worden. Vul een 0,1,2 of 9 in in de ruimte ‘vul hier in’.*

| **01. WONEN** |
| --- |

| HEEFT DE PERSOON EEN GESCHIKTE PLAATS OM TE WONEN? | **Vul hier in:** |
| --- | --- |

### Hoe is de woonsituatie van uw naaste? Heeft uw naaste problemen met zijn/haar woonsituatie? (ontvangt uw naaste hulp van vrienden, verwanten of plaatselijke voorzieningen bij het wonen?

0 = GEEN BEHOEFTE (bijv. heeft een gepaste en geschikte woonsituatie (ook indien momenteel in ziekenhuis). Heeft geen behoefte aan hulp bij wonen.)

1 = TEGEMOETGEKOMEN BEHOEFTE (bijv. de woning wordt aangepast/opgeknapt. Heeft hulp nodig en krijgt deze ook, bijvoorbeeld in een verzorgingshuis, beschermd wonen.)

2 = BESTAANDE BEHOEFTE (bijv. dakloos, geen geschikte onderkomen, er ontbreken basisvoorzieningen zoals water, elektriciteit, verwarming of essentiële aanpassingen)

9 = ONBEKEND

| **02. HUISHOUDEN** . |
| --- |

| HEEFT DE PERSOON PROBLEMEN MET HET HUISHOUDEN? | **Vul hier in:** |
| --- | --- |

**Kan uw naaste zelf het huishouden doen? Helpt iemand uw naaste? (ontvangt uw naaste hulp van vrienden, verwanten of plaatselijke voorzieningen bij het wonen?)**

0 = GEEN BEHOEFTE (bijv. Doet het huishouden zelfstandig. Het huis is misschien niet opgeruimd, maar wel schoon.)

1 = TEGEMOETGEKOMEN BEHOEFTE (bijv. Kan maar beperkt voor het huis zorgen en heeft regelmatig huishoudelijke hulp.)

2 = BESTAANDE BEHOEFTE (bijv. Krijgt geen gepaste hulp bij het huishouden. De woning is een potentieel gevaar voor de gezondheid, brandveiligheid of vluchtmogelijkheid.)

9 = ONBEKEND

| **03. VOEDING** . |
| --- |

| HEEFT DE PERSOON PROBLEMEN MET HET KRIJGEN VAN VOLDOENDE VOEDING? | **Vul hier in:** |
| --- | --- |

**Kan uw naaste zelf zijn of haar maaltijden bereiden en zijn of haar eigen boodschappen doen? Krijgt uw naaste de juiste voeding? (ontvangt uw naaste hulp van vrienden, verwanten of plaatselijke voorzieningen bij het krijgen van voldoende voeding?)**

0 = GEEN BEHOEFTE (bijv. In staat om gepaste maaltijden te kopen en/ of te bereiden.)

1 = TEGEMOETGEKOMEN BEHOEFTE (bijv. Niet in staat eten te bereiden, maar krijgt maaltijden of hulp om de behoefte te ondervangen.)

2 = BESTAANDE BEHOEFTE (bijv. Zeer beperkt dieet; eten past niet bij cultuur; kan niet het juiste voedsel verkrijgen; heeft moeite normaal voedsel door te slikken.)

9 = ONBEKEND

| **04. ZELFZORG** |
| --- |

| HEEFT DE PERSOON PROBLEMEN MET ZELFZORG? | **Vul hier in:** |
| --- | --- |

**Heeft uw naaste problemen met de persoonlijke verzorging, zoals wassen, nagels knippen of aankleden? Heeft uw naaste wel eens hulp nodig? (ontvangt uw naaste hulp van vrienden, verwanten of plaatselijke voorzieningen bij de zelfzorg?)**

0 = GEEN BEHOEFTE (bijv. Zorgt zelf voor een goed gekleed en verzorgd uiterlijk.)

1 = TEGEMOETGEKOMEN BEHOEFTE (bijv. Heeft passende hulp nodig bij zelfzorg en krijgt deze.)

2 = BESTAANDE BEHOEFTE (bijv. Slechte persoonlijke hygiëne, kan zich niet wassen en aankleden en krijgt hierbij geen passende hulp.)

9 = ONBEKEND

| **05. VOOR IEMAND ANDERS ZORGEN** . |
| --- |

| HEEFT DE PERSOON PROBLEMEN MET HET ZORGEN VOOR EEN ANDER? | **Vul hier in:** |
| --- | --- |

**Zorgt uw naaste voor iemand? Heeft uw naaste problemen met het zorgen voor die persoon? (ontvangt uw naaste hulp van vrienden, verwanten of plaatselijke voorzieningen bij het zorgen voor een ander?)**

| 0 = GEEN BEHOEFTE (bijv. Heeft niemand om voor te zorgen, of heeft geen problemen met zorgen.)  1 = TEGEMOETGEKOMEN BEHOEFTE (bijv. Problemen met zorgen, maar krijgt hulp.)    2 = BESTAANDE BEHOEFTE (bijv. Grote problemen met het zorgen voor en verzorgen van de persoon.)  9 = ONBEKEND |
| --- |

| **06. DAGBESTEDING** . |
| --- |

| HEEFT DE PERSOON PROBLEMEN MET REGELMATIGE, PASSENDE DAGBESTEDING? | **Vul hier in:** |
| --- | --- |

**Hoe brengt uw naaste de dag door? Heeft uw naaste genoeg te doen? (ontvangt uw naaste hulp van vrienden, verwanten of plaatselijke voorzieningen bij het vinden van een passende dagbesteding?)**

| 0 = GEEN BEHOEFTE (bijv. Gepaste sociale-, werk- of vrijetijdsactiviteiten, kan eigen activiteiten organiseren.)  1 = TEGEMOETGEKOMEN BEHOEFTE (bijv. Enigszins beperkt in zichzelf bezighouden, maar laat anderen passende activiteiten organiseren.)  2 = BESTAANDE BEHOEFTE (bijv. Geen gepaste sociale, werk- of vrijetijdsactiviteiten)  9 = ONBEKEND |
| --- |

| **07. GEHEUGEN** . |
| --- |

| HEEFT DE PERSOON PROBLEMEN MET ZIJN GEHEUGEN? | **Vul hier in:** |
| --- | --- |

**Heeft uw naaste vaak moeite om zich zaken die onlangs zijn voorgevallen te herinneren? Vergeet uw naaste vaak waar hij of zij dingen heeft opgeborgen? (ontvangt uw naaste hulp van vrienden, verwanten of plaatselijke voorzieningen in verband met geheugenproblemen?)**

| 0 = GEEN BEHOEFTE (bijv. Vergeet wel af en toe, maar herinnert het zich naderhand. Geen problemen met het geheugen.)  1 = TEGEMOETGEKOMEN BEHOEFTE (bijv. Enige problemen, maar wordt onderzocht/ heeft hulp.)    2 = BESTAANDE BEHOEFTE (bijv. Duidelijk beperkt in het zich herinneren van nieuwe informatie: raakt dingen kwijt, is gedesoriënteerd in tijd en/of plaats. Krijgt geen passende begeleiding.)  9 = ONBEKEND |
| --- |

| **08. GEZICHTSVERMOGEN, GEHOOR** **& COMMUNICATIE** . |
| --- |

| HEEFT DE PERSOON PROBLEMEN MET ZIEN OF HOREN? | **Vul hier in:** |
| --- | --- |

**Heeft uw naaste, in een stille kamer, moeite met het verstaan van wat iemand tegen hem of haar zegt? Heeft uw naaste moeite met de krant lezen of televisie kijken? Kan uw naaste duidelijk maken wat hij of zij bedoelt? (ontvangt uw naaste hulp van vrienden, verwanten of plaatselijke voorzieningen voor problemen met zien of horen?)**

| 0 = GEEN BEHOEFTE (bijv. Geen problemen (draagt misschien bril/ lenzen of gehoorapparaat, is zelfstandig).  1 = TEGEMOETGEKOMEN BEHOEFTE (bijv. Enige problemen, maar de hulpmiddelen helpen enigszins. Ontvangt de juiste onderzoeken en/ of hulp bij het onderhouden van de hulpmiddelen.)  2 = BESTAANDE BEHOEFTE (bijv. Veel problemen met zien of horen, ontvangt geen gepaste begeleiding.)  9 = ONBEKEND |
| --- |

| **09. MOBILITEIT/ VALLEN**  . |
| --- |

| HEEFT DE PERSOON BEWEGINGSBEPERKINGEN, VALNEIGINGEN OF PROBLEMEN BIJ GEBRUIK VAN OPENBAAR VERVOER? | **Vul hier in:** |
| --- | --- |

**Heeft uw naaste moeite om zich binnenshuis te verplaatsen? Valt uw naaste wel eens?**

**Heeft uw naaste problemen met transport? (ontvangt uw naaste hulp van vrienden, verwanten of plaatselijke voorzieningen in verband met mobiliteitsbehoefte?)**

| 0 = GEEN BEHOEFTE (bijv. Gezond en mobiel.)  1 = TEGEMOETGEKOMEN BEHOEFTE (bijv. Enige moeite met lopen, traplopen of gebruik van openbaar vervoer, maar functioneert met hulp (bijv. stok, looprek, rollater).  Valt af en toe. Veiligheidsplan aanwezig.)  2 = BESTAANDE BEHOEFTE (bijv. Zeer bewegingsbeperkt, zelfs met hulpmiddel. Valt meerdere keren per maand. Passende hulp ontbreekt.)  9 = ONBEKEND |
| --- |

| **10. CONTINENTIE** |
| --- |

| IS DE PERSOON INCONTINENT? | **Vul hier in:** |
| --- | --- |

**Heeft uw naaste wel eens een ongelukje/ dat uw naaste nat is als hij of zij niet snel genoeg bij de toilet is? Hoe groot is het probleem? Ook wel ontlasting? Krijgt uw naaste enige hulp? (ontvangt uw naaste hulp van vrienden, verwanten of lokale voorzieningen in verband met incontinentie?)**

| 0 = GEEN BEHOEFTE (bijv. Geen incontinentie. Zelfstandig in het omgaan met incontinentie.)  1 = TEGEMOETGEKOMEN BEHOEFTE(bijv. Enige incontinentie. Ontvangt gepaste hulp/ onderzoek.)    2 = BESTAANDE BEHOEFTE (bijv. Regelmatig nat of bevuild. Verslechterende continentie, wat beoordeling behoeft.)  9 = ONBEKEND |
| --- |

| **11. LICHAMELIJKE GEZONDHEID**  . |
| --- |

| LIJDT DE PERSOON AAN EEN LICHAMELIJKE ZIEKTE? | **Vul hier in:** |
| --- | --- |

**Hoe voelt uw naaste zich lichamelijk? Wordt uw naaste door zijn of haar dokter behandeld voor lichamelijke problemen? (ontvangt uw naaste hulp van vrienden, verwanten of plaatselijke voorzieningen in verband met lichamelijke gezondheidsproblemen?)**

| 0 = GEEN BEHOEFTE (bijv. Lichamelijk gezond. Ontvangt geen medische interventies.)  1 = TEGEMOETGEKOMEN BEHOEFTE (bijv. Lichamelijke kwaal, zoals hoge bloeddruk, is onder controle, ontvangt gepaste behandeling. Beoordelingen van de fysieke toestand.)  2 = BESTAANDE BEHOEFTE (bijv. Onbehandelde ernstige lichamelijke kwaal. Veel pijn. Wacht op grote medische ingreep.  9 = ONBEKEND |
| --- |

| **12. MEDICATIE** |
| --- |

| HEEFT DE PERSOON PROBLEMEN MET MEDICIJNEN? | **Vul hier in:** |
| --- | --- |

**Heeft uw naaste problemen (bijv. bijwerkingen) met medicatie? Hoeveel verschillende medicijnen gebruikt uw naaste? Is zijn of haar medicatie onlangs nog beoordeeld door zijn of haar dokter? Gebruikt uw naaste medicijnen die niet zijn voorgeschreven? (Ontvangt uw naaste hulp van vrienden, verwanten of plaatselijke voorzieningen bij zijn of haar medicatie)**

| 0 = GEEN BEHOEFTE (bijv. Geen probleem met medicijninname, bijwerkingen, medicatiemisbruik of afhankelijkheid.  1 = TEGEMOETGEKOMEN BEHOEFTE (bijv. Regelmatige beoordeling medicatie, advies, wijkverpleegkundige, huisarts, doseringsdoos/ hulpmiddelen.)  2 = BESTAANDE BEHOEFTE (bijv. Slechte inname medicijnen, neemt te veel of te weinig. Afhankelijk of misbruik van voorgeschreven of niet-voorgeschreven middelen.)  9 = ONBEKEND |
| --- |

| **13. PSYCHOTISCHE SYMPTOMEN** |
| --- |

| HEEFT DE PERSOON SYMPTOMEN ZOALS WANEN, HALLUCINATIES, FORMELE DENKSTOORNISSEN OF PASSIVITEIT? | **Vul hier in:** |
| --- | --- |

**Heeft uw naaste ooit stemmen gehoord, vreemde dingen gezien, of problemen met zijn of haar denken? Gebruikt uw naaste hiervoor medicatie? (Ontvangt uw naaste hulp van vrienden, familie of lokale voorzieningen voor psychotische symptomen?)**

| 0 = GEEN BEHOEFTE (bijv. Geen vaststelbare symptomen. Geen risico voor of in nood doorsymptomen en geen medicatie voor psychotische symptomen)  1 = TEGEMOETGEKOMEN BEHOEFTE (bijv. Symptomen verlicht door medicatie of andere hulp, bijvoorbeeld omgangsstrategieën, veiligheidsplan.)  2 = BESTAANDE BEHOEFTE (bijv. Heeft momenteel symptomen of risico daarop.)  9 = ONBEKEND |
| --- |

| **14. PSYCHISCHE NOOD** |
| --- |

| IS DE PERSOON MOMENTEEL IN PSYCHISCHE NOOD? | **Vul hier in:** |
| --- | --- |

**Heeft uw naaste zich onlangs somber gevoeld of dat uw naaste het zat was? Heeft uw naaste zich heel angstig, bang of bezorgd gevoeld? (ontvangt uw naaste hulp van vrienden, familie of plaatselijke voorzieningen voor nood?)**

| 0 = GEEN BEHOEFTE (bijv. Incidentele of milde spanning. Kan hier zelfstandig mee omgaan.)  1 = TEGEMOETGEKOMEN BEHOEFTE (bijv. Heeft doorlopend hulp nodig en krijgt deze.)    2 = BESTAANDE BEHOEFTE (bijv. Spanning tast leven wezenlijk aan, bijv. verhindert persoon om buiten te komen.)  9 = ONBEKEND |
| --- |

| **15. INFORMATIE**  (OVER GEZONDHEIDSTOESTAND & BEHANDELING) |
| --- |

| HEEFT DE PERSOON DUIDELIJKE VERBALE OF GESCHREVEN INFORMATIE OVER ZIJN GEZONDHEIDSTOESTAND EN BEHANDELING GEKREGEN? | **Vul hier in:** |
| --- | --- |

**Heeft uw naaste duidelijke informatie over zijn of haar toestand, medicatie of andere behandeling gekregen? Wenst uw naaste dergelijke informatie? Hoe nuttig is de informatie geweest? (Ontvangt uw naaste hulp van vrienden, verwanten of lokale voorzieningen om deze informatie te krijgen?)**

| 0 = GEEN BEHOEFTE (bijv. Heeft adequate informatie, ontvangen en begrepen. Heeft geen informatie ontvangen maar wil deze ook niet.)  1 = TEGEMOETGEKOMEN BEHOEFTE (bijv. Krijgt hulp om de informatie te begrijpen. De gegeven informatie sluit aan op het communicatie/ begripsniveau van dat van de oudere.)  2 = BESTAANDE BEHOEFTE (bijv. Heeft inadequate of geen informatie ontvangen.)  9 = ONBEKEND |
| --- |

| **16. OPZETTELIJK GEVAAR VOOR ZICHZELF** . |
| --- |

| IS DE PERSOON EEN GEVAAR VOOR ZICHZELF? | **Vul hier in:** |
| --- | --- |

**Denkt uw naaste er ooit over om zichzelf kwaad te doen of doet uw naaste zich daadwerkelijk kwaad? (ontvangt uw naaste hulp van vrienden, familie of plaatselijke voorzieningen om het risico op opzettelijk gevaarlijk gedrag te verminderen?)**

| 0 = GEEN BEHOEFTE (bijv. Geen gedachten over gevaarlijk gedrag of zelfmoord/ suïcide.)  1 = TEGEMOETGEKOMEN BEHOEFTE (bijv. Suïciderisico wordt in de gaten gehouden door hulpverlener, krijgt gesprekken.)  2 = BESTAANDE BEHOEFTE (bij. Uit zelfmoordneigingen, verwaarloost zichzelf opzettelijk of heeft zich de laatste maand aan groot gevaar blootgesteld.)  9 = ONBEKEND |
| --- |

| **17. ONOPZETTELIJK GEVAAR VOOR ZICHZELF** . |
| --- |

| IS DE PERSOON ONBEDOELD EEN GEVAAR VOOR ZICHZELF? | **Vul hier in:** |
| --- | --- |

**Doet uw naaste ooit iets dat hem of haar onbedoeld in gevaar brengt? (bijv. gas aanlaten, vuur onbewaakt laten of verdwalen)? (ontvangt uw naaste hulp van vrienden, familie of plaatselijke voorzieningen om onbedoeld gevaarlijk gedrag te verminderen?)**

| 0 = GEEN BEHOEFTE (bijv. Geen onopzettelijk gevaar voor zichzelf.)  1 = TEGEMOETGEKOMEN BEHOEFTE (bijv. Specifiek toezicht of hulp om gevaar te voorkomen: bijv. geheugensteuntjes, aansporingen, veilige omgeving en/ of observatie.)  2 = BESTAANDE BEHOEFTE (bijv. Frequent gevaarlijk gedrag, bijv. verdwalen, gas/ brandrisico.)  9 = ONBEKEND |
| --- |

| **18. MISBRUIK EN VERWAARLOZING**  . |
| --- |

| LOOPT DE PERSOON EEN RISICO DOOR ANDEREN? | **Vul hier in:** |
| --- | --- |

### Heeft iemand iets gedaan om uw naaste bang te maken, uw naaste kwaad te doen of van uw naaste te profiteren? (ontvangt uw naaste hulp van vrienden, familie of plaatselijke diensten om risico op misbruik, mishandeling te verminderen?)

| 0 = GEEN BEHOEFTE (bijv. Geen misbruik of verwaarlozing in de laatste maand.)  1 = TEGEMOET GEKOMEN BEHOEFTE (bij. Behoeft en krijgt voortdurend steun of bescherming.)    2 = BESTAANDE BEHOEFTE (bijv. Regelmatig schreeuwen, duwen of verwaarlozing. Onrechtmatig toe-eigenen van geld, fysiek geweld/ bedreiging.)  9 = ONBEKEND |
| --- |

| **19. GEDRAG**  . |
| --- |

| IS HET GEDRAG VAN DE PERSOON GEVAARLIJK, BEDREIGEND, BEMOEIZUCHTIG OF STOREND VOOR ANDEREN? | **Vul hier in:** |
| --- | --- |

**Komt uw naaste in conflict met anderen b.v. door bemoeienis met hun zaken, ze regelmatig te ergeren, te bedreigen of te storen? Wat gebeurt er dan? (Ontvangt uw naaste hulp van vrienden, familie of plaatselijke voorzieningen om het risico van ergerlijk of storend gedrag te verminderen?)**

| 0 = GEEN BEHOEFTE (bijv. Heeft geen geschiedenis van storend gedrag.  1 = TEGEMOET GEKOMEN BEHOEFTE (bij. Onder toezicht of behandeling vanwege mogelijk risico.)  2 = BESTAANDE BEHOEFTE (bijv. Recent geweld, dreigementen of zeer bemoeizuchtig gedrag.)  9 = ONBEKEND |
| --- |

| **20. ALCOHOL** |
| --- |

| DRINKT DE PERSOON BUITENSPORIG VEEL OF HEEFT HIJ EEN PROBLEEM MET HET ONDER CONTROLE HOUDEN VAN DRANKGEBRUIK? | **Vul hier in:** |
| --- | --- |

**Drinkt uw naaste alcohol? Hoeveel? Heeft uw naaste problemen met drinken? Voelt uw naaste zich daar ooit schuldig over? Wenst uw naaste wel eens dat hij of zij zijn of haar drankgebruik kon minderen? (Ontvangt uw naaste hulp van vrienden, familie of lokale voorzieningen in verband met drankgebruik?)**

| 0 = GEEN BEHOEFTE (bijv. Drinkt niet of gecontroleerd.)  1 = TEGEMOET GEKOMEN BEHOEFTE (bijv. Loopt risico op alcoholmisbruik en ontvangt hulp.)    2 = BESTAANDE BEHOEFTE (bijv. Huidig drinkgedrag schadelijk of oncontroleerbaar. Krijgt geen passende hulp.)  9 = ONBEKEND |
| --- |

| **21. GEZELSCHAP** |
| --- |

| HEEFT DE PERSOON HULP NODIG BIJ SOCIAAL CONTACT | **Vul hier in:** |
| --- | --- |

**Is uw naaste gelukkig met zijn of haar sociale leven? Zou uw naaste willen dat hij of zij meer sociaal contact had? (Ontvangt uw naaste hulp van vrienden, familie of plaatselijke voorzieningen voor sociale contacten?)**

| 0 = GEEN BEHOEFTE (bijv. In staat om voldoende sociaal contact te arrangeren, heeft voldoende contact met vrienden.)  1 = TEGEMOETGEKOMEN BEHOEFTE (bijv. Het gebrek aan contact wordt als probleem gezien. Krijgt ondersteuning vanwege behoefte aan gezelschap, b.v. is ’s avonds eenzaam, maar bezoekt inloop- of dagcentra etc. Inmenging van maatschappelijk werk.)  2 = BESTAANDE BEHOEFTE (bijv. Voelt zich vaker eenzaam en geïsoleerd. Heel weinig sociale contacten)  9 = ONBEKEND |
| --- |

| **22. INTIEME RELATIES** |
| --- |

| HEEFT DE PERSOON EEN PARTNER, VERWANTE OF VRIEND MET WIE HIJ EEN INTIEME/ HECHTE EMOTIONELE/ FYSIEKE RELATIE HEEFT? | **Vul hier in:** |
| --- | --- |

**Heeft uw naaste een partner, verwante of vriend met wie hij of zij zich verbonden voelt? Kan uw naaste goed met ze opschieten? Kan uw naaste spreken over zijn of haar zorgen en problemen? Ontbreekt het uw naaste aan fysiek contact/ intimiteit? (Ontvangt uw naaste hulp van vrienden, familie of plaatselijke voorzieningen in verband met intieme relaties of eenzaamheid?)**

| 0 = GEEN BEHOEFTE (bijv. Gelukkig met huidige relaties of wil geen intieme relatie.)  1 = TEGEMOETGEKOMEN BEHOEFTE (bijv. Counseling/ advies, dat behulpzaam is.)    2 = BESTAANDE BEHOEFTE (bijv. Wanhopig eenzaam. Gebrek aan vertrouweling.)  9 = ONBEKEND |
| --- |

| **23. GELD** |
| --- |

| HEEFT DE PERSOON PROBLEMEN OM ZIJN GELD TE BEHEREN OF TE BUDGETTEREN? | **Vul hier in:** |
| --- | --- |

**Heeft uw naaste problemen om zijn of haar geld te beheren? Kan uw naaste zijn of haar rekeningen betalen? (ontvangt uw naaste hulp van vrienden, familie of plaatselijke voorzieningen voor het beheren van zijn of haar geld?)**

| 0 = GEEN BEHOEFTE (bijv. In staat om noodzakelijke goederen te kopen en rekeningen te betalen.)  1 = TEGEMOETGEKOMEN BEHOEFTE (bijv. Profiteert van hulp bij het beheren en budgetteren van geld.)    2 = BESTAANDE BEHOEFTE (bijv. Heeft geen geld voor noodzakelijke goederen of rekeningen. Niet in staat om financiële zaken te regelen.)  9 = ONBEKEND |
| --- |

| **24. TOELAGEN / UITKERINGEN** |
| --- |

| ONTVANGT DE PERSOON ECHT ALLE TOELAGEN/ UITKERINGEN WAAR HIJ RECHT OP HEEFT? | **Vul hier in:** |
| --- | --- |

**Weet uw naaste zeker dat hij of zij al het geld krijgt waar hij of zij recht op heeft? (Ontvangt uw naaste hulp van vrienden, familie of plaatselijke voorzieningen voor het verkrijgen van alle toelagen en uitkeringen waar hij of zij recht op heeft?)**

| 0 = GEEN BEHOEFTE (bijv. Heeft geen behoefte aan uitkeringen of ontvangt alles waarop men recht heeft.)  1 = TEGEMOETGEKOMEN BEHOEFTE (bijv. Krijgt adequate hulp bij het vorderen van aanvraag uitkeringen.)    2 = BESTAANDE BEHOEFTE (bijv. Onzeker/ ontvangt niet alle gelden waarop men recht heeft.)    9 = ONBEKEND |
| --- |

*!! LET OP!!! Hierna volgen twee vragen die gaan* ***OVER DE MANTELZORGER***

| **A. INFORMATIEBEHOEFTE MANTELZORGER**  . |
| --- |

| HEEFT DE MANTELZORGER HELDERE INFORMATIE ONTVANGEN OVER DE GEZONDHEID VAN DE PERSOON EN BESCHIKBARE HANDELINGEN? | **Vul hier in:** |
| --- | --- |

**Heeft u duidelijke informatie gekregen over de gezondheid van uw naaste en de beschikbare**

**behandelingen en voorzieningen? Hoe nuttig is deze informatie geweest? (Ontvangt u hulp van vrienden, familie of plaatselijke voorzieningen voor het verkrijgen van informatie?)**

| 0 = GEEN BEHOEFTE (bijv. Ontvangen en begrepen.)  1 = TEGEMOETGEKOMEN BEHOEFTE (bijv. Heeft niet alle informatie gekregen of begrepen. Krijgt hulp met informatie.)  2 = BESTAANDE BEHOEFTE (bijv. Heeft weinig of geen informatie gekregen. Heeft de informatie niet begrepen.)  9 = ONBEKEND |
| --- |

| **B. PSYCHISCHE NOOD MANTELZORGER** |
| --- |

| HEEFT DE MANTELZORGER MOMENTEEL PSYCHISCHE NOOD? | **Vul hier in:** |
| --- | --- |

**Vindt u het moeilijk of belastend om voor uw naaste te zorgen? Heeft u behoefte aan een rustperiode of aan veel meer steun voor uzelf? (Ontvangt u hulp van vrienden, familie of lokale voorzieningen voor psychische nood?)**

| 0 = GEEN BEHOEFTE (bijv. Gaat er goed mee om.)  1 = TEGEMOET GEKOMEN BEHOEFTE (bijv. Enige belasting: ontvangt hulp/ contact/ steun die ook  BEHOEFTE daadwerkelijk helpt.  2 = BESTAANDE BEHOEFTE bijv. Beschouwd zichzelf zeer belast of gedeprimeerd. Wil ondersteund worden in de zorg.  9 = ONBEKEND |
| --- |

*Dit is het einde van het interview. Heeft de mantelzorger opmerkingen? Schrijf die dan hieronder op:*

| **Observaties van de interviewer** |
| --- |

*Vul hier belangrijke informatie in die mogelijk invloed heeft gehad op de antwoorden van de respondent. Hij of zij kan bijvoorbeeld ziek zijn of net zijn geweest of door een emotionele gebeurtenis (begrafenis) van slag zijn. Het is belangrijk deze zaken vast te leggen, daar kun je deze ruimte voor gebruiken.*
