# Supplementary material for: The Cost-Effectiveness of Two Forms of Case Management Compared to a Control Group for Persons with Dementia and Their Informal Caregivers from a Societal Perspective
Source: PLoS One. 2016 Sep 21;11(9):e0160908. doi: 10.1371/journal.pone.0160908 (PMC5031395; doi:10.1371/journal.pone.0160908)
Supplement: S2 Case Record Form — Interview with the person with dementia (in Dutch). (DOC) [file pone.0160908.s004.doc]

# Interview zorgvrager

# T0

# Inhoud

- Registratie

- leiding voor interviewer

- Inleiding voor zorgvrager

- Kwaliteit van leven (MDS, EQ5D+C)

- Werking van het geheugen (MMSE)

- Kwaliteit van leven (Qol-AD)

## - Ruimte voor opmerkingen

## Inleiding voor interviewer

Deze vragenlijsten worden afgenomen in de vorm van een interview bij personen met dementie. Voor een goede afname dienen alle instructies letterlijk te worden opgevolgd en de vragen precies volgens de bewoording te worden gesteld.

Begin met deze inleiding voor de zorgvrager. Vertel de inleiding en noem de aandachtspunten die hier staan. Zorg ervoor dat de inleiding zoals jij hem verteld overeenkomt met de inleiding die hier staat maar laat het overkomen alsof je uit je hoofd wat verteld. Ga dit dus niet voorlezen.

# Inleiding voor zorgvrager

Ik ga u een aantal vragen stellen over uw leven op dit moment. Daarna ga ik een aantal vragen stellen over uw behoeften op het gebied van zorg. Uw antwoorden worden gebruikt in onderzoek naar verbetering van de zorg voor ouderen. Uw ervaringen zijn dus waardevol, ook voor andere ouderen.

Uw antwoorden worden anoniem verwerkt en uw gegevens worden niet verder verspreid.

Uw antwoorden op de vragen hebben geen invloed op de zorg die u ontvangt.

- Het beantwoorden van de vragen kost u ongeveer een half uur.
- Ik wil u vragen om goed naar de hele vraag te luisteren voordat u een antwoord kiest.
- Geef het antwoord aan dat het beste bij u past.
- Geef bij elke vraag maar één antwoord.
- Als u meer dan één antwoord mag geven, dan wordt dat bij de vraag genoemd.
- Als u het moeilijk vindt om de vragen te begrijpen of te beantwoorden, vraagt u mij dan om hulp of uitleg .
- Sommige vragen lijken misschien dubbel maar het antwoord is toch heel waardevol. Ze zijn bedoeld om uw situatie nog eens van een andere kant te bekijken.

| **Registratie** |
| --- |

Studienummer:

Interviewer:

BSN nummer patiënt:

Respondentnummer (patiënt):

Respondentnummer (mantelzorger):

Geboortedatum respondent (patiënt):

Datum afname:

| **Kwaliteit van leven** (MDS, EQ-5D+c) |
| --- |

Overhandig de deelnemer het formulier

De volgende vragen gaan over hoe het vandaag met u gaat. Geef aan welke zin het beste past bij uw gezondheid zoals die nu is.

1. Lopen:

□ Ik heb geen problemen met lopen

□ Ik heb enige problemen met lopen

□ Ik ben bedlegerig

2. Zelfzorg:

□ Ik heb geen problemen om mezelf te wassen of aan te kleden

□ Ik heb enige problemen om mezelf te wassen of aan te kleden

□ Ik ben niet in staat om mezelf te wassen of aan te kleden

3. Dagelijkse activiteiten (bijvoorbeeld werk, studie, huishouden, gezins- en vrijetijdsactiviteiten):

□ Ik heb geen problemen met mijn dagelijkse activiteiten.

□ Ik heb enige problemen met mijn dagelijkse activiteiten.

□ Ik ben niet in staat mijn dagelijkse activiteiten uit te voeren.

4. Pijn/klachten:

□ Ik heb geen pijn of andere klachten.

□ Ik heb matige pijn of andere klachten.

□ Ik heb zeer ernstige pijn of andere klachten.

5. Stemming:

□ Ik ben niet angstig of somber.

□ Ik ben matig angstig of somber.

□ Ik ben erg angstig of somber.

6. Hersenfuncties zoals geheugen, aandacht en denken:

□ Ik heb geen problemen met mijn geheugen, aandacht en denken.

□ Ik heb enige problemen met mijn geheugen, aandacht en denken.

□ Ik heb ernstige problemen met mijn geheugen, aandacht en denken.

| **Werking van het geheugen** (MMSE) |
| --- |

Ik ga u een aantal vragen stellen om de werking van uw geheugen en concentratie na te gaan.

Vul de gegeven antwoorden in. Scoor één punt voor elk juist item.

1

1. Welk jaar is het? a.
2. Welk seizoen is het? b.
3. Welke maand van het jaar is het? c.
4. Wat is de datum vandaag? d.
5. Welke dag van de week is het? e.

Score (0-5): ____

Vul de gegeven antwoorden in. Scoor één punt voor elk juist item

2

1. In welke provincie zijn we nu? a.
2. In welke plaats zijn we nu? b.
3. Waar zijn we nu? c.
4. Wat is de naam van de straat? d.
5. Op welk huisnummer zijn we nu? e.

Score (0-5): ­­­____

3

Ik noem nu drie voorwerpen. Wilt u die herhalen nadat ik ze alle drie gezegd heb? Onthoud ze want ik vraag u over enkele minuten ze opnieuw te noemen.

(Noem: ‘’appel, sleutel, tafel’’, neem 1 seconde per woord) (1 punt voor elk goed antwoord, herhaal maximaal 5 keer tot de patiënt de drie woorden weet)

4

1. Wilt u van 100 zeven aftrekken en van wat overblijft weer zeven aftrekken en zo doorgaan tot ik stop zeg? (Herhaal eventueel 3 maal als de persoon stopt, herhaal dezelfde instructie, geef maximaal 1 minuut de tijd, stop bij 65)

Noteer hier het antwoord: ____ Score (0-5): ____

1. Wilt u het woord ‘’worst’’ achterstevoren spellen?

Noteer hier het antwoord: ____ Score (0-5): ____

5

Noemt u nogmaals de drie voorwerpen van zojuist.

1. Appel
2. Sleutel
3. Tafel

Scoor één punt voor elk goed antwoord

Score (0-3): ____

6

1. Wat is dit?
2. En wat is dat?

(Wijs een pen en een horloge aan. Eén punt voor elk goed antwoord

Score (0-2): ____

*7*

Wilt u de volgende zin herhalen: ‘Nu eens dit en dan weer dat.’

Scoor één punt als de complete zin goed is.

Score (0-1): ____

8

Wilt u deze woorden lezen en dan doen wat er staat?

(Laat een papier zien met daarop in grote letters: ‘’Sluit uw ogen’’)

Scoor één punt als de opdracht goed wordt uitgevoerd.

Score (0-1): ____

Vertel de zorgvrager dat zijn of haar ogen weer open mogen.

9

Wilt u dit papiertje pakken met uw rechterhand, het dubbelvouwen en het op uw schoot leggen?

(Scoor één punt voor iedere goede handeling)

Score (0-3): ____

10

Wilt u voor mij een volledige zin opschrijven op dit stuk papier?

(Scoor één punt wanneer de zin een onderwerp, een gezegde en betekenis heeft.)

Score (0-1): ____

11

Wilt u deze figuur natekenen?

(Laat de figuur op het papier zien en overhandig het papier zodat de deelnemer het kan natekenen. Eén punt als figuur geheel correct is nagetekend. Er moet een vierhoek te zien zijn tussen de twee vijfhoeken)

Score (0-1): ____

Totale Test Score: _____

| Kwaliteit van leven (QoL-AD) |
| --- |

Overhandig de deelnemer het formulier, zodat hij of zij ernaar kan kijken terwijl jij de vragen voorleest. De vragen dienen nauwkeurig en precies volgens de bewoording te worden gesteld.

Ik wil u een aantal vragen stellen over uw kwaliteit van leven en uw een waardering laten geven over verschillende aspecten van uw leven door gebruik te maken van vier woorden: slecht, redelijk, goed, of uitstekend.

Wijs naar elk woord (slecht, redelijk, goed of uitstekend) op het formulier terwijl je ze opnoemt.

Wanneer u nadenkt over uw leven, dan bestaat deze uit verschillende aspecten, zoals uw lichamelijke gezondheid, vitaliteit, familie, geld en dergelijke. Ik ga u vragen om elk van deze aspecten een waardering te geven. We willen nagaan hoe u zich voelt over uw huidige situatie op elk van deze gebieden.

Als u niet zeker weet wat de vraag betekent, kunt u mij daarover vragen stellen. Als u moeite heeft met het geven van een waardering op gebied, geef dan gewoon uw beste gok.

Het is over het algemeen duidelijk of een persoon de vragen begrijpt, en de meeste personen die in staat zijn om te communiceren en te antwoorden op simpele vragen kunnen de vragenlijst begrijpen. Als de deelnemer alle vragen hetzelfde beantwoordt, of iets zegt dat een gebrek aan begrip indiceert, wordt de interviewer aangemoedigd om de vraag te verhelderen. Hoe dan ook, de interviewer mag onder geen beding een specifiek antwoord suggereren. Elk van de vier mogelijke antwoorden moet worden aangeboden en de deelnemer moet een van de vier kiezen.

Als een deelnemer niet in staat is een antwoord te kiezen op een bepaald onderdeel of onderdelen, moet dit genoteerd worden in het commentaar. Als de deelnemer niet in staat is twee of meer onderdelen te begrijpen of te beantwoorden mag het onderzoek afgebroken worden, dit moet genoteerd worden in het commentaar.

Wanneer je de onderaan opgestelde vragen voorleest, vraag de deelnemer zijn/haar antwoorden te omcirkelen. Als de deelnemer moeite heeft met het omcirkelen van het woord, mag je hem/haar vragen het antwoord aan te wijzen of het antwoord te benoemen en jij mag het voor hem of haar omcirkelen. Je dient de deelnemer zijn of haar eigen formulier vast te laten houden en mee te laten lezen terwijl je ieder onderdeel voorleest.

1. Ten eerste, wat vindt u van uw lichamelijke gezondheid? Zou u zeggen dat die slecht, redelijk, goed of uitstekend is? Omcirkel nu welk woord u denkt dat het beste uw lichamelijke gezondheid omschrijft.

2. Wat is uw mening over uw vitaliteit? Vindt u dat deze slecht, redelijk, goed of uitstekend is?

Als de deelnemer zegt dat sommige dagen beter zijn dan andere, vraag hem/haar te bepalen hoe hij/zij zich meestal voelde de laatste tijd.

3. Hoe was uw stemming de laatste tijd? Voelde u zich prettig, of heeft u zich somber gevoeld? Zou u uw stemming als slecht, redelijk, goed of uitstekend beoordelen?

4. Wat vindt u van uw woonomstandigheden? Wat vindt u van de plek waar u nu woont? Zou u zeggen dat deze slecht, redelijk, goed of uitstekend is?

5. Wat vindt u van uw geheugen? Vindt u dat dit slecht, redelijk, goed of uitstekend is?

6. Wat vindt u van uw familie en uw relatie met uw familieleden? Zou u dit omschrijven als slecht, redelijk, goed of uitstekend?

Als de deelnemer zegt geen familie (meer) te hebben, vraag dan naar broers, zussen, kinderen, neven.

7. Wat vindt u van uw huwelijk? Hoe is uw relatie met (naam partner)? Vindt u dat die slecht, redelijk, goed of uitstekend is?

Sommige deelnemers zullen alleenstaand, weduwnaar/weduwe of gescheiden zijn. Wanneer dat het geval is, vraag wat zij vinden van de persoon met wie zij de meest intieme relatie hebben, of dat nu een familielid is of niet. Als er een familie ‘caregiver’ is, vraag naar hun relatie met deze persoon. Als er niemand in aanmerking komt, of de deelnemer is onzeker, scoor dit onderdeel dan als ‘missing’.

8. Hoe zou u uw relatie met uw vrienden omschrijven? Vindt u die slecht, redelijk goed of uitstekend?

Als de deelnemer antwoordt dat ze geen vrienden hebben, of al hun vrienden overleden zijn, probeer verder.

Heeft u iemand wiens gezelschap u op prijs stelt, naast uw familie? Zou u die persoon een vriend noemen?

Als de deelnemer nog steeds zegt dat ze geen vrienden hebben, vraag dan:

Hoe voelt dat om geen vrienden te hebben: slecht, redelijk, goed of uitstekend?

9. Wat vindt u van uzelf? Wanneer u denkt aan uw gehele persoon, en al de verschillende aspecten over uzelf, zou u zeggen dat u dat slecht, redelijk, goed of uitstekend vindt?

10. Wat vindt u van uw vermogen om dingen als karweitjes rond het huis te doen of andere dingen die u moet doen? Zou u zeggen dat, dat slecht, redelijk, goed of uitstekend is?

11. Wat vindt u van uw vermogen om leuke dingen te doen, waar u plezier aan beleeft? Vindt u dat slecht, redelijk, goed of uitstekend?

12. Wat vindt u van uw huidige situatie wat betreft geld, uw financiële situatie? Vindt u deze slecht, redelijk, goed of uitstekend?

Als de deelnemer aarzelt, leg dan uit dat je niet wilt weten wat hun situatie is (wat betreft hoeveelheid geld), maar dat je alleen wilt weten wat zij vinden van hun situatie.

13. Hoe zou u uw leven over het geheel omschrijven? Wanneer u nadenkt over uw leven als geheel, alles bij elkaar genomen, hoe denkt u dan over uw leven? Vindt u dat slecht, redelijk, goed of uitstekend?

**Dit is het einde van het interview**

Bedank de deelnemer en geef aan dat eventuele opmerkingen nog gemaakt kunnen worden.

Schrijf deze opmerkingen op de volgende pagina.

# Ruimte voor opmerkingen

Heeft de zorgvrager nog opmerkingen, schrijf ze dan hieronder op:

| **Observaties van de interviewer** |
| --- |

Vul hier belangrijke informatie in die mogelijk invloed heeft gehad op de antwoorden van de respondent. Hij of zij kan bijvoorbeeld ziek zijn of net zijn geweest of door een emotionele gebeurtenis (begrafenis) van slag zijn. Het is belangrijk deze zaken vast te leggen, daar kun je deze ruimte voor gebruiken.

1. Lopen:

□ Ik heb geen problemen met lopen

□ Ik heb enige problemen met lopen

□ Ik ben bedlegerig

2. Zelfzorg:

□ Ik heb geen problemen om mezelf te wassen of aan te kleden

□ Ik heb enige problemen om mezelf te wassen of aan te kleden

□ Ik ben niet in staat om mezelf te wassen of aan te kleden

3. Dagelijkse activiteiten (bijvoorbeeld werk, studie, huishouden, gezins- en vrijetijdsactiviteiten):

□ Ik heb geen problemen met mijn dagelijkse activiteiten.

□ Ik heb enige problemen met mijn dagelijkse activiteiten.

□ Ik ben niet in staat mijn dagelijkse activiteiten uit te voeren.

4. Pijn/klachten:

□ Ik heb geen pijn of andere klachten.

□ Ik heb matige pijn of andere klachten.

□ Ik heb zeer ernstige pijn of andere klachten.

5. Stemming:

□ Ik ben niet angstig of somber.

□ Ik ben matig angstig of somber.

□ Ik ben erg angstig of somber.

6. Hersenfuncties zoals geheugen, aandacht en denken:

□ Ik heb geen problemen met mijn geheugen, aandacht en denken.

□ Ik heb enige problemen met mijn geheugen, aandacht en denken.

□ Ik heb ernstige problemen met mijn geheugen, aandacht en denken.

**Sluit uw ogen**


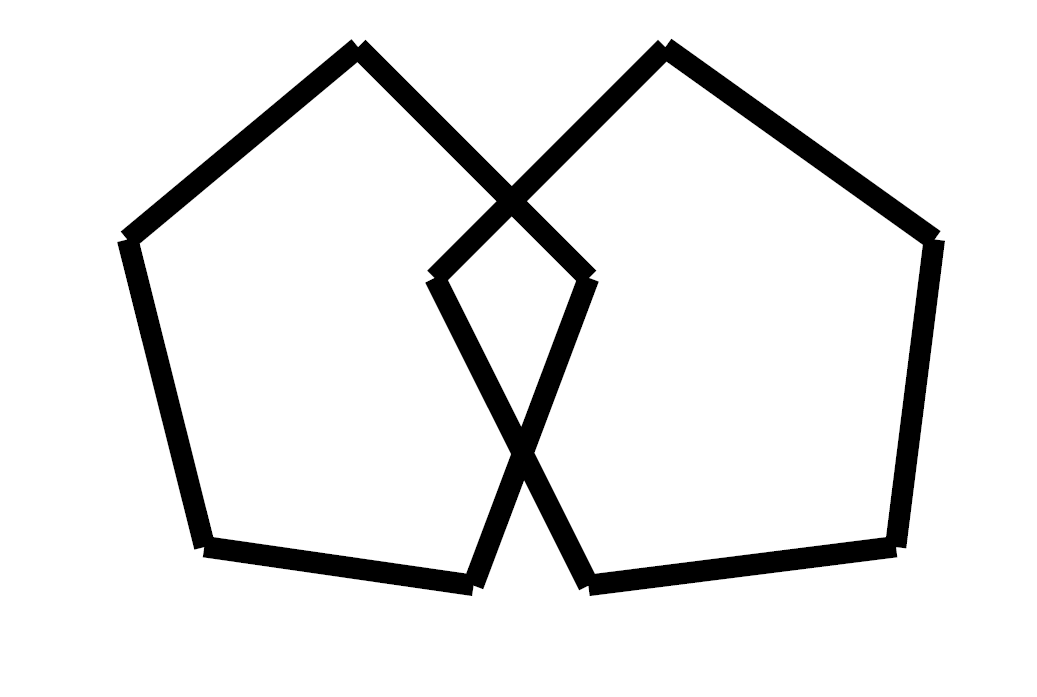


| 1. Lichamelijke gezondheid | Slecht | Redelijk | Goed | Uitstekend |
| --- | --- | --- | --- | --- |
| 2. Vitaliteit | Slecht | Redelijk | Goed | Uitstekend |
| 3. Stemming | Slecht | Redelijk | Goed | Uitstekend |
| 4. Woonomstandigheden | Slecht | Redelijk | Goed | Uitstekend |
| 5. Geheugen | Slecht | Redelijk | Goed | Uitstekend |
| 6. Familie | Slecht | Redelijk | Goed | Uitstekend |
| 7. Huwelijk | Slecht | Redelijk | Goed | Uitstekend |
| 8. Vrienden | Slecht | Redelijk | Goed | Uitstekend |
| 9. Persoon als geheel | Slecht | Redelijk | Goed | Uitstekend |
| 10. Vermogen om karweitjes te verrichten | Slecht | Redelijk | Goed | Uitstekend |
| 11. Vermogen om leuke dingen te doen | Slecht | Redelijk | Goed | Uitstekend |
| 12. Geld | Slecht | Redelijk | Goed | Uitstekend |
| 13. Leven als geheel | Slecht | Redelijk | Goed | Uitstekend |
