# Supplementary material for: The Cost-Effectiveness of Two Forms of Case Management Compared to a Control Group for Persons with Dementia and Their Informal Caregivers from a Societal Perspective
Source: PLoS One. 2016 Sep 21;11(9):e0160908. doi: 10.1371/journal.pone.0160908 (PMC5031395; doi:10.1371/journal.pone.0160908)
Supplement: S3 Case Record Form — Questionnaire filled in by informal caregiver (in Dutch). (DOC) [file pone.0160908.s005.doc]

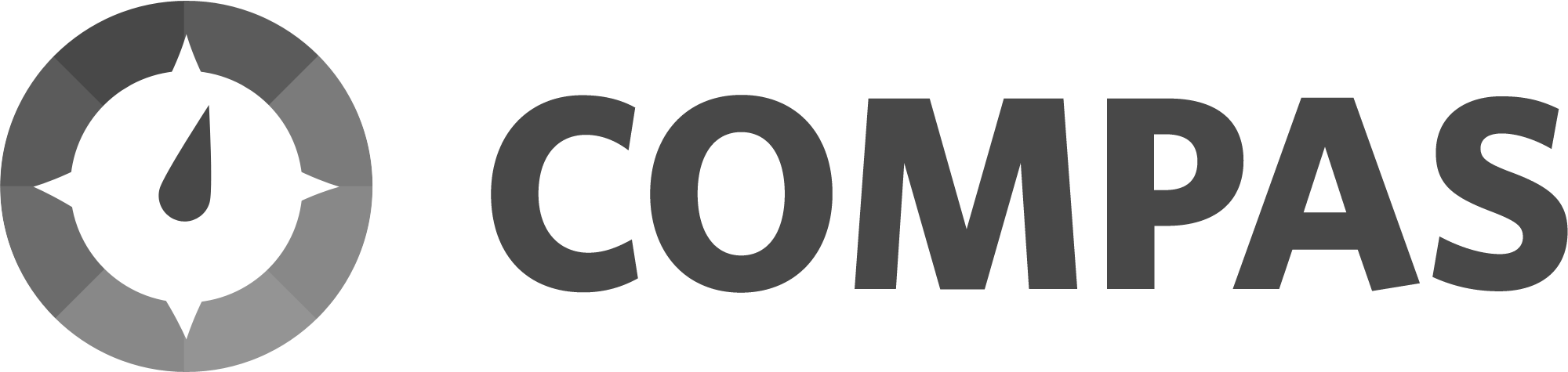


**Vragenlijst voor de mantelzorger**


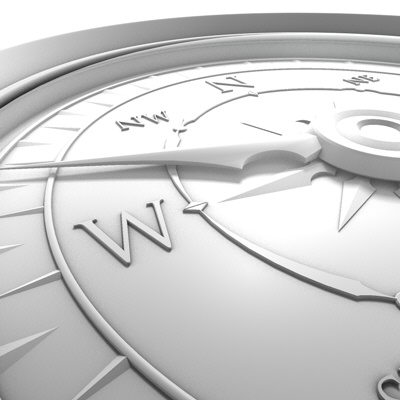


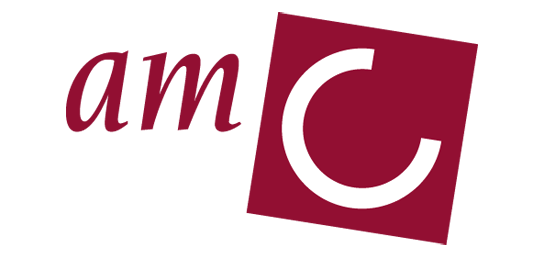


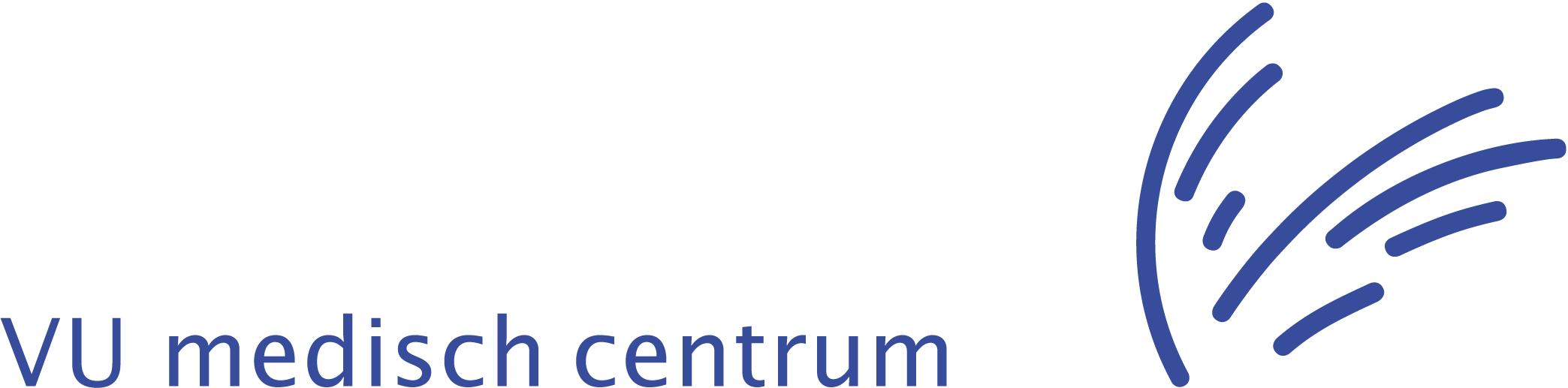


**Inhoudsopgave**

- Uw ervaringen zijn waardevol
- Aandachtspunten bij het invullen van de vragenlijst

**De vragenlijsten**

Over uzelf

- Persoonlijke gegevens
- Psychische gezondheid
- Gevoel van competentie
- Sociale steun en eenzaamheid
- Hoe gaat het met u?
- Controle over uw leven
- Uw ervaren problemen
- Uw gezondheid en kwaliteit van leven
- Hoe zwaar is het voor u om zorg te verlenen?
- Uw situatie als mantelzorger
- Hoe gelukkig bent u of zou u zijn?
- Welke informatie over dementie gebruikt u?
- Ontvangen ondersteuning
- Aangeraden diensten
- Uw gezondheid

Over uw naaste

- Persoonlijke gegevens van uw naaste
- Functioneren van uw naaste
- Gezondheid en kwaliteit van leven van uw naaste
- Gezondheid van uw naaste

**Uw ervaringen zijn waardevol**

U en degene voor wie u zorgt, maken deel uit van de COMPAS studie. Met behulp van uw ervaringen kan de zorg voor mensen met dementie en de hulp die geboden wordt aan mantelzorgers steeds beter worden afgestemd op de behoeftes die zij hebben. Uw ervaringen met betrekking tot het zorgen voor uw naaste zijn daarom voor ons van groot belang.

De vragenlijst die u voor u heeft liggen is zeer divers. We willen graag wat weten over uw kwaliteit van leven, uw gezondheid (zowel lichamelijk als psychisch) uw ervaren belasting en nog veel meer. Alle gegevens helpen ons een beter beeld te vormen over uw huidige situatie.

Omdat wij ook informatie over uw naaste willen verzamelen bevat deze vragenlijst ook vragen over degene voor wie u zorgt. Wij verwachten dat u alle vragen goed kunt beantwoorden. Als er echter onduidelijkheden zijn horen we dat graag.

U kunt bellen naar de veldwerkcoördinator Eveline Spalburg (020-4446327). U mag uw vragen ook aan de interviewer stellen wanneer deze bij u langskomt. We helpen u graag om de vragen te begrijpen.

Wanneer de onderzoekers uw antwoorden gaan bekijken zijn deze van te voren door een computerprogramma gecodeerd. Dat betekend dat het onbekend is wie welke antwoorden heeft gegeven. Ook worden uw gegevens nooit verder verspreid. Het invullen van deze vragenlijst heeft geen invloed op de zorg die u of uw naaste ontvangt.

Het invullen neemt ongeveer 30 minuten in beslag.

**Aandachtspunten bij het invullen van de vragenlijst**

- Als u leest ‘uw naaste’, dan gaat de vraag over de persoon voor wie u mantelzorger bent.
- Als de vragen over u gaan, staat er ‘uzelf’.
- Lees elke vraag eerst helemaal door voordat u antwoord geeft.
- Kruis dan het antwoord aan dat het beste bij u of uw naaste past.
- Als u meer dan één antwoord aan mag kruisen, dan wordt dat bij de vraag genoemd
- Kruis bij alle andere vragen maar één antwoord aan.
- Bent u klaar, controleer dan nog even of u geen vragen vergeten bent.

Het is belangrijk dat u alle vragen beantwoordt, ook al vindt u het soms moeilijk om een antwoord te geven. Er zijn geen goede of foute antwoorden.

Wij wensen u veel succes met het invullen van de vragenlijst en willen u graag bij voorbaat bedanken voor uw tijd. U kunt beginnen met invullen op de volgende pagina.

Vriendelijke groeten,

Het onderzoeksteam

In te vullen door de interviewer:

Studienummer: NL32949.029.10

Interviewernummer:

Care receiver number:

Geboortedatum mantelzorger:

Vragenlijst is afgenomen met behulp van: (omcirkel wat van toepassing is)

1= een face to face interview

2= een interview over de telefoon

3= een geschreven/gemailde of webbased vragenlijst (internet)

4= een gemailde vragenlijst welke achteraf gecontroleerd is met een interview

5= een gemailde vragenlijst welke achter gecontroleerd is met een telefonisch interview

**Dit is het begin van de vragenlijst**

Vult u alstublieft de datum van vandaag in: (Voorbeeld: 01-01-2011)

--

Over uzelf

| **Persoonlijke gegevens** |
| --- |

1. Wat is uw geboortedatum?

--

2. Wat is uw geslacht?

□ Man □ Vrouw

3. Wat zijn de cijfers van uw postcode?



4. Wat is uw relatie met uw naaste?

□ Echtgenoot, echtgenote of levenspartner

□ Schoonzus, zus, broer of zwager

□ Schoondochter, dochter, schoonzoon of zoon

□ Anders, namelijk:

|  |
| --- |

5. Woont u samen met uw naaste?

□ Ja □ Nee

6. Wat is de hoogste opleiding die u heeft afgerond?

□ Minder dan 6 klassen lagere school

□ 6 klassen lagere school / lom-school / mlk-school

□ Meer dan lagere school / basisschool zonder verder afgesloten opleiding

□ Mulo / mms / mavo / middelaarberoepsonderwijs

□ Hbs / gymnasium / atheneum

□ Universiteit / hoger onderwijs

| **Psychische Gezondheid** (GHQ12) |
| --- |

De volgende vragen gaan over uw psychische gezondheid. We willen graag weten of u medische problemen heeft gehad en hoe uw algemene gezondheid is geweest de afgelopen weken. Geef alstublieft het antwoord dat het beste bij uw situatie past. Onthoud dat we willen weten welke klachten u nu heeft of recent gehad heeft. Het gaat dus niet over klachten uit het verleden.

1. Bent u de laatste tijd door zorgen veel slaap tekort gekomen?

□ Helemaal niet

□ Niet meer dan gewoonlijk

□ Iets meer dan gewoonlijk

□ Veel meer dan gewoonlijk

2. Heeft u de laatste tijd het gevoel gehad dat u voortdurend onder druk stond?

□ Helemaal niet

□ Niet meer dan gewoonlijk

□ Iets meer dan gewoonlijk

□ Veel meer dan gewoonlijk

3. Heeft u zich de laatste tijd kunnen concentreren op uw bezigheden?

□ Beter dan gewoonlijk

□ Net zo goed als gewoonlijk

□ Slechter dan gewoonlijk

□ Veel slechter dan gewoonlijk

4. Heeft u de laatste tijd het gevoel gehad zinvol bezig te zijn?

□ Zinvoller dan gewoonlijk

□ Net zo zinvol als gewoonlijk

□ Minder zinvol dan gewoonlijk

□ Veel minder zinvol dan gewoonlijk

5. Bent u de laatste tijd in staat geweest uw problemen onder ogen te zien?

□ Beter in staat dan gewoonlijk

□ Net zo goed in staat als gewoonlijk

□ Minder goed in staat dan gewoonlijk

□ Veel minder goed in staat gewoonlijk

6. Voelde u zich de laatste tijd in staat om beslissingen (over dingen) te nemen?

□ Beter in staat dan gewoonlijk

□ Net zo goed in staat als gewoonlijk

□ Minder goed in staat dan gewoonlijk

□ Veel minder goed in staat gewoonlijk

7. Heeft u de laatste tijd het gevoel gehad dat u uw moeilijkheden niet de baas kon?

□ Nee, ik had dat gevoel helemaal niet

□ Niet minder de baas dan gewoonlijk

□ Iets minder de baas dan gewoonlijk

□ Veel minder de baas dan gewoonlijk

8. Heeft u zich de laatste tijd alles bij elkaar redelijk gelukkig gevoeld?

□ Gelukkiger dan gewoonlijk

□ Even gelukkig als gewoonlijk

□ Minder gelukkig dan gewoonlijk

□ Veel minder gelukkig dan gewoonlijk

9. Heeft u de laatste tijd plezier kunnen beleven aan uw gewone, dagelijkse bezigheden?

□ Meer dan gewoonlijk

□ Evenveel als gewoonlijk

□ Iets minder dan gewoonlijk

□ Veel minder dan gewoonlijk

10. Heeft u zich de laatste tijd ongelukkig en neerslachtig gevoeld?

□ Helemaal niet

□ Niet meer dan gewoonlijk

□ Iets meer dan gewoonlijk

□ Veel meer dan gewoonlijk

11. Bent u de laatste tijd het vertrouwen in uzelf kwijtgeraakt?

□ Helemaal niet

□ Niet meer dan gewoonlijk

□ Iets meer dan gewoonlijk

□ Veel meer dan gewoonlijk

12. Heeft u zich de laatste tijd als een waardeloos iemand beschouwd?

□ Helemaal niet

□ Niet meer dan gewoonlijk

□ Iets meer gewoonlijk

□ Veel meer dan gewoonlijk

| **Gevoel van competentie** (SSCQ) |
| --- |

De volgende uitspraken zijn samengesteld op basis van gesprekken met mensen, die zich in een zelfde situatie bevonden als u. De uitspraken gaan over gevoelens die sommigen van hen hadden. Wij willen nu graag weten of u die gevoelens herkent. Het gaat daarbij om uw gevoelens van de afgelopen week. Geeft u alstublieft per vraag aan in hoeverre u het met de uitspraak eens bent. Hieronder staat aangegeven wat de antwoordopties betekenen:

ja! = (helemaal mee eens)

ja = (mee eens)

min of meer = (enerzijds mee eens / anderzijds niet mee eens)

nee = (niet mee eens)

nee! = (helemaal niet mee eens)

|  | **Nee!** | **Nee** | **Min of meer** | **Ja** | **Ja!** |
| --- | --- | --- | --- | --- | --- |
| De huidige situatie met mijn naaste gunt mij niet zoveel privacy als ik zou willen | □ | □ | □ | □ | □ |
|  | | | | | |
| De verantwoordelijkheid voor mijn naaste valt mij zwaar naast al mijn andere verantwoordelijkheden (gezin, werk enz.) | □ | □ | □ | □ | □ |
|  | | | | | |
| Ik zou willen dat mijn naaste en ik een betere relatie hadden | □ | □ | □ | □ | □ |
|  | | | | | |
| Ik voel mij gespannen in mijn contact met mijn naaste | □ | □ | □ | □ | □ |
|  | | | | | |
| Ik heb het gevoel dat mijn naaste zich zo gedraagt om mij te manipuleren | □ | □ | □ | □ | □ |

ja! = (helemaal mee eens)

ja = (mee eens)

min of meer = (enerzijds mee eens / anderzijds niet mee eens)

nee = (niet mee eens)

nee! = (helemaal niet mee eens)

|  | **Nee!** | **Nee** | **Min of meer** | **Ja** | **Ja!** |
| --- | --- | --- | --- | --- | --- |
| Ik heb het gevoel dat mijn naaste zich zo gedraagt, omdat hij/zij mij dwars wil zitten | □ | □ | □ | □ | □ |
|  | | | | | |
| Ik heb het gevoel dat mijn naaste zich zo gedraagt om zijn/haar zin te krijgen | □ | □ | □ | □ | □ |

| **Eenzaamheid** (loneliness) |
| --- |

Wilt u van elk van de volgende uitspraken aangeven in hoeverre die op u, zoals u de laatste tijd bent, van toepassing is? Kruis het antwoord aan dat op u van toepassing is.

|  |  | **Nee** | **Min of meer** | **Ja** |
| --- | --- | --- | --- | --- |
| **1** | Er is altijd wel iemand in mijn omgeving bij wie ik met mijn dagelijkse probleempjes terecht kan | □ | □ | □ |
|  | | | | |
| **2** | Ik mis een echt goede vriend of vriendin | □ | □ | □ |
|  | | | | |
| **3** | Ik ervaar een leegte om me heen | □ | □ | □ |
|  | | | | |
| **4** | Er zijn genoeg mensen op wie ik in geval van narigheid kan terugvallen | □ | □ | □ |
|  | | | | |
| **5** | Ik mis gezelligheid om me heen | □ | □ | □ |
|  | | | | |
| **6** | Ik vind mijn kring van kennissen te beperkt | □ | □ | □ |
|  | | | | |
| **7** | Ik heb veel mensen op wie ik volledig kan vertrouwen | □ | □ | □ |
|  | | | | |
| **8** | Er zijn voldoende mensen met wie ik me nauw verbonden voel | □ | □ | □ |
|  | | | | |
| **9** | Ik mis mensen om me heen | □ | □ | □ |
|  | | | | |
| **10** | Vaak voel ik me in de steek gelaten | □ | □ | □ |
|  | | | | |
| **11** | Wanneer ik daar behoefte aan heb kan ik altijd bij mijn vrienden terecht | □ | □ | □ |

| **Hoe gaat het met u?** (EQ5D) |
| --- |

De volgende vragen gaan over hoe het vandaag met u gaat. Kruis aan

welke zin het beste past bij uw gezondheid zoals die nu is.

1. Lopen

□ Ik heb geen problemen met lopen

□ Ik heb enige problemen met lopen

□ Ik ben bedlegerig

2. Zelfzorg

□ Ik heb geen problemen om mezelf te wassen of aan te kleden

□ Ik heb enige problemen om mezelf te wassen of aan te kleden

□ Ik ben niet instaat mezelf te wassen of aan te kleden

3. Dagelijkse activiteiten (bijvoorbeeld: werk, studie, huishouden, gezins- en vrijetijdsactiviteiten)

□ Ik heb geen problemen met mijn dagelijkse activiteiten

□ Ik heb enige problemen met mijn dagelijkse activiteiten

□ Ik ben niet in staat mijn dagelijkse activiteiten uit te voeren

4. Pijn/klachten

□ Ik heb geen pijn of andere klachten

□ Ik heb matige pijn of andere klachten

□ Ik heb zeer ernstige pijn of andere klachten

5. Stemming

□ Ik ben niet angstig of somber

□ Ik ben matig angstig of somber

□ Ik ben erg angstig of somber

| **Controle over uw leven** (Mastery) |
| --- |

De volgende vragen gaan over uw gevoel van controle in uw leven. Kruis bij elke vraag één vakje aan.

1. Ik heb weinig controle over de dingen die me overkomen.

□ Helemaal mee oneens

□ Mee oneens

□ Noch mee eens noch mee oneens

□ Mee eens

□ Helemaal mee eens

2. Sommige van mijn problemen kan ik met geen mogelijkheid oplossen.

□ Helemaal mee oneens

□ Mee oneens

□ Noch mee eens noch mee oneens

□ Mee eens

□ Helemaal mee eens

3. Er is weinig dat ik kan doen om belangrijke dingen in mijn leven

te veranderen.

□ Helemaal mee oneens

□ Mee oneens

□ Noch mee eens noch mee oneens

□ Mee eens

□ Helemaal mee eens

4. Ik voel me vaak hulpeloos bij het omgaan met de problemen van het

leven.

□ Helemaal mee oneens

□ Mee oneens

□ Noch mee eens noch mee oneens

□ Mee eens

□ Helemaal mee eens

5. Soms voel ik dat ik een speelbal van het leven ben.

□ Helemaal mee oneens

□ Mee oneens

□ Noch mee eens noch mee oneens

□ Mee eens

□ Helemaal mee eens

| **Uw ervaren problemen** (NIVEL) |
| --- |

De volgende vragen gaan over problemen die u kunt ervaren. Wilt u aankruisen wat voor u van toepassing is?

**1. Niet pluisgevoel & Wat is er aan de hand en wat kan helpen**

A. Nam de huisarts u serieus bij de eerste vermoedens van dementie?

□ Ja

□ Eigenlijk wel

□ Eigenlijk niet

□ Nee

□ Niet van toepassing

B. Kreeg u voldoende uitleg over dementie en de verschijnselen die daarbij horen?

□ Ja

□ Eigenlijk wel

□ Eigenlijk niet

□ Nee

□ Niet van toepassing

C. Kreeg u voldoende uitleg over het verloop van dementie?

□ Ja

□ Eigenlijk wel

□ Eigenlijk niet

□ Nee

□ Niet van toepassing

D. Hoe belangrijk zijn bovenstaande problemen voor u op dit moment?

□ Van het allergrootste belang

□ Heel belangrijk

□ Belangrijk

□ Niet zo belangrijk

**2. Miscommunicatie met zorgverleners**

A. Sluit de professionele zorg/hulp die u ontvangt aan bij uw wensen?

□ Ja

□ Eigenlijk wel

□ Eigenlijk niet

□ Nee

□ Niet van toepassing

B. Vindt u dat zorg-/hulpverleners ook voldoende aandacht voor u hebben?

□ Ja

□ Eigenlijk wel

□ Eigenlijk niet

□ Nee

□ Niet van toepassing

C. Hoe belangrijk zijn bovenstaande problemen voor u op dit moment?

□ Van het allergrootste belang

□ Heel belangrijk

□ Belangrijk

□ Niet zo belangrijk

**3. Weerstand tegen opname**

A. Ziet u op tegen opname van uw naaste in een zorginstelling?

□ Ja

□ Eigenlijk wel

□ Eigenlijk niet

□ Nee

□ Niet van toepassing

B. Krijgt u voldoende ondersteuning bij een mogelijke opname van uw naaste?

□ Ja

□ Eigenlijk wel

□ Eigenlijk niet

□ Nee

□ Niet van toepassing

C. Hoe belangrijk zijn bovenstaande problemen voor u op dit moment?

□ Van het allergrootste belang

□ Heel belangrijk

□ Belangrijk

□ Niet zo belangrijk

| **Uw gezondheid en kwaliteit van leven** (MDS) |
| --- |

De volgende vragen gaan over uw gezondheid en uw kwaliteit van leven.

Kruis het hokje aan van het antwoord dat het beste bij u past.

1. Hoe is in het algemeen uw gezondheid?

□ Uitstekend

□ Erg goed

□ Goed

□ Redelijk

□ Slecht

2. Hoe is in het algemeen uw gezondheid, in vergelijking met een jaar geleden?

□ Veel beter

□ Iets beter

□ Ongeveer hetzelfde

□ Iets slechter

□ Veel slechter

3. Hoe is in het algemeen uw kwaliteit van leven?

□ Uitstekend

□ Erg goed

□ Goed

□ Redelijk

□ Slecht

4. Hoe is in het algemeen uw kwaliteit van leven, in vergelijking met een jaar geleden?

□ Veel beter

□ Iets beter

□ Ongeveer hetzelfde

□ Iets slechter

□ Veel slechter

5. Welk rapportcijfer geeft u uw leven op dit moment? (Voorbeeld: 07)

Vul het rapportcijfer (tussen 1-10) in: 

| **Hoe zwaar is het voor u om zorg te verlenen** (MDS) |
| --- |

Deze vraag gaat over hoe zwaar het voor u is om zorg te geven. Geef met een kruisje op de meetlat aan hoe zwaar u het vindt om zorg aan uw naaste te geven. Hoe dichter bij de ‘0’ hoe minder zwaar u de zorg vindt, hoe dichter bij de ‘10’ hoe zwaarder u de zorg vindt. Voor de duidelijkheid wordt er eerst een voorbeeld gegeven.

| **Voorbeeld:**  Hoe lekker vindt u chocolade-ijs?   | Helemaal niet lekker Heel erg lekker | | --- |  |  |  |  |  |  |  | X |  |  |  | | --- | --- | --- | --- | --- | --- | --- | --- | --- | --- | | 1 | 2 | 3 | 4 | 5 | 6 | 7 | 8 | 9 | 10 | |
| --- | --- | --- | --- | --- | --- | --- | --- | --- | --- | --- | --- | --- | --- | --- | --- | --- | --- | --- | --- | --- | --- |

**Zet een kruisje op de meetlat:**

Hoe zwaar vindt u het om zorg aan uw naaste te geven?

| Helemaal niet zwaar Veel te zwaar |
| --- |

|  |  |  |  |  |  |  |  |  |  |
| --- | --- | --- | --- | --- | --- | --- | --- | --- | --- |
| 1 | 2 | 3 | 4 | 5 | 6 | 7 | 8 | 9 | 10 |

| **Uw situatie als mantelzorger**  (MDS, Carer-QoL) |
| --- |

De volgende vragen gaan over uw zorgsituatie als mantelzorger.

Zet een kruisje bij het woord dat het beste past bij uw zorgsituatie. U kunt bij iedere vraag kiezen uit: geen, enige of veel.

|  |  | **Geen** | **Enige** | **Veel** |
| --- | --- | --- | --- | --- |
| 1. | Ik heb voldoening van het verrichten van mijn zorgtaken | □ | □ | □ |
|  | | | | |
| 2. | Ik heb problemen met mijn naaste (bijvoorbeeld: hij/zij is veeleisend, we hebben communicatieproblemen, hij/zij is zich anders gaan gedragen. | □ | □ | □ |
|  | | | | |
| 3. | Ik heb problemen met mijn eigen geestelijke gezondheid (bijvoorbeeld stress, angst, somberheid, bezorgdheid over de toekomst) | □ | □ | □ |
|  | | | | |
| 4. | Ik heb problemen met mijn eigen lichamelijke gezondheid (bijvoorbeeld vaker ziek, vermoeidheid, lichamelijke overbelasting) | □ | □ | □ |
|  | | | | |
| 5. | Ik heb problemen met mijn dagelijkse activiteiten (bijvoorbeeld werk, huishouden, studie, gezin en vrije tijd) te combineren met mijn zorgtaken | □ | □ | □ |
|  | | | | |
| 6. | Ik heb financiële problemen bij mijn zorgtaken | □ | □ | □ |
|  | | | | |
| 7. | Ik heb steun (van familie/vrienden/buren/kennissen/vrijwilligers) bij het verrichten van mijn zorgtaken | □ | □ | □ |

| **Hoe gelukkig bent u of zou u zijn?** (MDS) |
| --- |

Er volgen nu twee vragen waarbij de meetlat weer wordt gebruikt.

1. Geef met een kruisje op de meetlat aan hoe gelukkig u uzelf op dit moment voelt.

| Volledig ongelukkig Volmaakt gelukkig |
| --- |

|  |  |  |  |  |  |  |  |  |  |
| --- | --- | --- | --- | --- | --- | --- | --- | --- | --- |
| 1 | 2 | 3 | 4 | 5 | 6 | 7 | 8 | 9 | 10 |

2. Stelt u zich voor: u en uw naaste mogen zelf iemand uitkiezen die al uw zorgtaken van u overneemt. Het kost niets extra en de persoon verleent alle overgenomen zorg bij uw naaste thuis. Geeft met een kruisje op de meetlat aan hoe gelukkig u zich zou voelen als deze persoon de zorg van u zou overnemen.

| Volledig ongelukkig Volmaakt gelukkig |
| --- |

|  |  |  |  |  |  |  |  |  |  |
| --- | --- | --- | --- | --- | --- | --- | --- | --- | --- |
| 1 | 2 | 3 | 4 | 5 | 6 | 7 | 8 | 9 | 10 |

Wilt u in het schema op de volgende pagina aangeven op welke manier u informatie krijgt/zoekt over de zorg rond dementie. Dit kan informatie zijn over de ziekte zelf, maar ook informatie over de mogelijkheid voor dagbesteding voor uw naaste.

Wilt u bij het invullen hiervan de afgelopen drie maanden in gedachten nemen?

| **Welke informatie over dementie gebruikt u?** |
| --- |

| Kunt u aangeven hoe vaak u in de afgelopen 3 maanden informatie over dementie heeft gebruikt van …? | | | | | Indien u deze informatie heeft gebruikt: Hoe tevreden bent u hierover? | | |
| --- | --- | --- | --- | --- | --- | --- | --- |
|  | nooit | soms | regel-  matig | vaak | Ontevreden | tevreden | Zeer tevreden |
| Huisarts | □ | □ | □ | □ | □ | □ | □ |
| Casemanager | □ | □ | □ | □ | □ | □ | □ |
| GGZ (voorheen RIAGG) | □ | □ | □ | □ | □ | □ | □ |
| Ziekenhuis | □ | □ | □ | □ | □ | □ | □ |
| Apotheek | □ | □ | □ | □ | □ | □ | □ |
| Wijkpost voor ouderen | □ | □ | □ | □ | □ | □ | □ |
| Dagbehandeling/Ontmoetingscentrum | □ | □ | □ | □ | □ | □ | □ |
| Centrum Indicatiestelling Zorg (CIZ) | □ | □ | □ | □ | □ | □ | □ |
| Alzheimer Nederland | □ | □ | □ | □ | □ | □ | □ |
| Mantelzorgorganisaties (b.v. MEE) | □ | □ | □ | □ | □ | □ | □ |
| Gemeente | □ | □ | □ | □ | □ | □ | □ |
| Overheid | □ | □ | □ | □ | □ | □ | □ |
| Anders, nl. ……………………………………… | □ | □ | □ | □ | □ | □ | □ |
| 2. Hoe vaak gebruikt u informatie van internet? | □ | □ | □ | □ | □ | □ | □ |

| **Ontvangen ondersteuning** (Vickrey) |
| --- |

Geef alstublieft bij iedere uitdrukking met ja of nee aan of u enige informatie, voorlichting of ondersteuning heeft ontvangen om u te helpen omgaan met probleem gedrag van uw naaste.

|  | **Markeer een box in elke rij** | |
| --- | --- | --- |
| In het afgelopen jaar: | **Ja** | **Nee** |
| heb ik voorlichting gehad over hoe ik moet reageren  op mijn naaste, bijvoorbeeld hoe ik hem of haar kan kalmeren of afleiden, hoe ik hem of haar kan geruststellen en hoe ik een discussie over een  onderwerp kan vermijden. | □ | □ |
|  | | |
| ben ik geadviseerd om te zorgen dat mijn naaste met dementie een voorspelbare, regelmatige routine van lichamelijke activiteit of beweging heeft. | □ | □ |
|  | | |
| ben ik geadviseerd om over-stimulatie te verminderen,  bijvoorbeeld, het verminderen van bezoek aan drukke  plaatsen, huishoudelijk geluiden van de tv of radio, of  rommel in huis. | □ | □ |
|  | | |
| ben ik verwezen naar respijtzorg zoals volwassenen  dagopvang of hoe ik nog een verzorger kan inhuren. | □ | □ |
|  | | |
| ben ik verwezen naar een organisatie uit de buurt of  bureau voor sociale voorzieningen (bijv Alzheimer  Nederland) voor hulp met het gedragsprobleem. | □ | □ |

| **Aangeraden diensten** (Vickrey) |
| --- |

Geef alstublieft voor iedere dienst die hieronder genoemd is aan of een arts, verpleegkundige of maatschappelijk werker of een andere professioneel verzorgende, familielid og vriend hierover heeft gepraat of heeft aangeraden deze dienst te gebruiken of u voor dit programma aan te melden in de afgelopen 12 maanden

|  | **Heeft iemand in de afgelopen 12 maanden met u gesproken of aangeraden deze dienst te gebruiken of u aan te melden voor dit programma?** | |
| --- | --- | --- |
|  | **Ja** | **Nee** |
| A. Mantelzorger ondersteuningsgroep | □ | □ |
|  | | |
| B. Respijtzorg, zoals dagbehandeling en oppas aan huis of andere respijt diensten | □ | □ |
|  | | |
| C. Juridisch advies omtrent financiële planning vanwege de dementie van uw naaste | □ | □ |

Geef alstublieft voor iedere organisatie die hieronder genoemd is aan of een dokter, verpleegkundige of maatschappelijk werker of een andere professioneel verzorgende, familielid of vriend hierover heeft gepraat of heeft aangeraden de diensten van deze organisatie te gebruiken of hier informatie over aan te vragen in de afgelopen 12 maanden

|  | **Heeft iemand in de afgelopen 12 maanden met u gesproken of aangeraden diensten van deze organisatie te gebruiken of hierover informatie aan te vragen?** | |
| --- | --- | --- |
|  | **Ja** | **Nee** |
| A. Een Alzheimer café in uw regio | □ | □ |
|  | | |
| B. Alzheimer Nederland | □ | □ |
|  | | |
| C. Mantelzorger steunpunt | □ | □ |
|  | | |
| D. Tafeltje – dek – je | □ | □ |
|  | | |
| E. Thuiszorg of een welzijnsvoorziening in uw gemeente | □ | □ |

| **Uw gezondheid** (MDS, GGD monitor) |
| --- |

De volgende vragen gaan over eventuele ziekten en aandoeningen die u heeft of heeft gehad. Zet een kruisje bij de ziekten en aandoeningen die u heeft of in de afgelopen 12 maanden heeft gehad. U kunt meer dan 1 antwoord aankruisen.

□ Suikerziekte

□ Beroerte, hersenbloeding, herseninfarct of TIA

□ Hartfalen

□ Een vorm van kanker (kwaadaardige aandoening)

□ Astma, chronische bronchitis, longemfyseem of CARA/COPD

□ Onvrijwillig urineverlies (incontinentie)

□ Gewrichtsslijtage (artrose, slijtagereuma van heupen of knieen)

□ Botontkalking (osteoporose)

□ Gebroken heup

□ Andere botbreuken dan gebroken heup

□ Duizeligheid met vallen

□ Prostaatklachten door goedaardige prostaatvergroting

□ Depressie

□ Angst- / paniekstoornis

□ Dementie

□ Gehoorproblemen

□ Problemen met zien

Over uw naaste

| **Persoonlijke gegevens van uw naaste** (MDS) |
| --- |

1. Wat is de geboortedatum van uw naaste?

--

2. Wat is het geslacht van uw naaste?

□ Man □ Vrouw

3. Wat zijn de cijfers van de postcode van uw naaste?



4. In welk land is uw naaste geboren?

□ Nederland

□ Ander land, namelijk:

|  |
| --- |

5. In welk land is de vader van uw naaste geboren?

□ Nederland

□ Ander land, namelijk:

|  |
| --- |

6. In welk land is de moeder van uw naaste geboren?

□ Nederland

□ Ander land, namelijk:

7. Wat is de hoogste opleiding die uw naaste heeft afgerond?

□ Minder dan 6 klassen lagere school

□ 6 klassen lagere school / lom-school / mlk-school

□ Meer dan lagere school / basisschool zonder verder afgesloten opleiding

□ Mulo / mms / mavo / middelaarberoepsonderwijs

□ Hbs / gymnasium / atheneum

□ Universiteit / hoger onderwijs

8. Wat is het laatste beroep wat uw naaste heeft uitgevoerd?

|  |
| --- |

9.Wat is de burgerlijke staat van uw naaste?

□ Getrouwd

□ Gescheiden

□ Weduwe / weduwnaar / partner overleden

□ Alleenstaand

□ Duurzaam samenlevend / ongehuwd

10. Wat is de woonsituatie van uw naaste?

□ Zelfstandig / alleen

□ Zelfstandig met anderen (partner, kinderen, enzovoorts)

□ Verzorgingshuis / woonzorgcentrum, sinds

--

□ Verpleeghuis, sinds

--

11. Wanneer u denkt aan de gezondheidszorg organisatie waarvan uw naaste zijn of haar meeste zorg ontvangt, hoe zou u in het algemeen de zorg die uw naaste de afgelopen zes maanden heeft ontvangen beoordelen?

(Vickrey)

| Slechts denkbare zorg Best denkbare zorg |
| --- |

|  |  |  |  |  |  |  |  |  |  |
| --- | --- | --- | --- | --- | --- | --- | --- | --- | --- |
| 1 | 2 | 3 | 4 | 5 | 6 | 7 | 8 | 9 | 10 |

□ Kruis aan indien u de afgelopen zes maanden geen contact hierover heeft gehad.

| **Functioneren van uw naaste**  (MDS,Katz15) |
| --- |

De volgende vragen gaan over hoe uw naaste functioneert in het dagelijks leven. Kies uw antwoord voor de situatie zoals deze nu is. Kruis het hokje aan van het antwoord dat het beste bij uw naaste past.

|  | **Ja** | **Nee** |
| --- | --- | --- |
| Heeft uw naaste hulp nodig bij het baden of douchen? | □ | □ |
|  |  |  |
| Heeft uw naaste hulp nodig bij het aankleden? | □ | □ |
|  |  |  |
| Heeft uw naaste hulp nodig bij het haren kammen of het scheren? | □ | □ |
|  |  |  |
| Heeft uw naaste hulp nodig met naar het toilet gaan? | □ | □ |
|  |  |  |
| Maakt uw naaste gebruik van incontinentiemateriaal? | □ | □ |
|  |  |  |
| Heeft uw naaste hulp nodig bij het opstaan uit een stoel? | □ | □ |
|  |  |  |
| Heeft uw naaste hulp nodig bij het lopen? | □ | □ |
|  |  |  |
| Heeft uw naaste hulp nodig bij het eten? | □ | □ |
|  |  |  |
| Heeft uw naaste hulp nodig bij het gebruiken van de telefoon? | □ | □ |
|  |  |  |
| Heeft uw naaste hulp nodig bij het reizen? | □ | □ |
|  |  |  |
| Heeft uw naaste hulp nodig bij het boodschappen doen? | □ | □ |
|  |  |  |
| Heeft uw naaste hulp nodig bij het bereiden van een maaltijd? | □ | □ |
|  |  |  |
| Heeft uw naaste hulp nodig bij huishoudelijk werk? | □ | □ |
|  |  |  |
| Heeft uw naaste hulp nodig bij het innemen van medicijnen? | □ | □ |
|  |  |  |
| Heeft uw naaste hulp nodig bij het omgaan met geld? | □ | □ |

| **Gezondheid en kwaliteit van leven van uw naaste** (MDS) |
| --- |

De volgende vragen gaan over de gezondheid en kwaliteit van leven van uw naaste.

Kruis het hokje aan van het antwoord dat het beste bij uw naaste past.

1. Hoe is in het algemeen de gezondheid van uw naaste?

□ Uitstekend

□ Erg goed

□ Goed

□ Redelijk

□ Slecht

2. Hoe is in het algemeen de gezondheid van uw naaste, in vergelijking met een jaar geleden?

□ Veel beter

□ Iets beter

□ Ongeveer hetzelfde

□ Iets slechter

□ Veel slechter

3. Hoe is in het algemeen de kwaliteit van leven van uw naaste?

□ Uitstekend

□ Erg goed

□ Goed

□ Redelijk

□ Slecht

4. Hoe is in het algemeen de kwaliteit van leven van uw naaste, in vergelijking met een jaar geleden?

□ Veel beter

□ Iets beter

□ Ongeveer hetzelfde

□ Iets slechter

□ Veel slechter

5. Welk rapportcijfer schat u in dat uw naaste zijn of haar leven op dit moment zou geven? (Voorbeeld: 07)

Vul het rapportcijfer, tussen 1-10 in: 

(1 is het laagste cijfer en 10 is het hoogste cijfer wat u kunt geven)

6. Hoe vaak hebben de lichamelijke gezondheid of emotionele problemen van uw naaste in de afgelopen 4 weken zijn of haar sociale activiteiten (zoals bezoek aan vrienden of naaste familieleden) belemmerd?

□ Voortdurend

□ Meestal

□ Soms

□ Zelden

□ Nooit

| **Gezondheid van uw naaste** (MDS, GGD monitor ) |
| --- |

De volgende vragen gaan over eventuele ziekten en aandoeningen die uw naaste heeft of heeft gehad. Zet een kruisje bij de ziekten en aandoeningen die uw naaste heeft of heeft gehad in de afgelopen 12 maanden. U mag meerdere antwoorden aankruisen.

□ Suikerziekte

□ Beroerte, hersenbloeding, herseninfarct of TIA

□ Hartfalen

□ Een vorm van kanker (kwaadaardige aandoening)

□ Astma, chronische bronchitis, longemfyseem of CARA/COPD

□ Onvrijwillig urineverlies (incontinentie)

□ Gewrichtsslijtage (artrose, slijtagereuma van heupen of knieen)

□ Botontkalking (osteoporose)

□ Gebroken heup

□ Andere botbreuken dan gebroken heup

□ Duizeligheid met vallen

□ Prostaatklachten door goedaardige prostaatvergroting

□ Depressie

□ Angst- / paniekstoornis

□ Dementie

□ Gehoorproblemen

□ Problemen met zien

**Dit is het eind van de vragenlijst**

Hartelijk bedankt voor het invullen!

De interviewer neemt de vragenlijst mee wanneer hij of zij bij u langskomt om te interviewen.

Als u nog opmerkingen heeft schrijft u die dan alstublieft hieronder op. Schrijft u alstublieft het nummer van de betreffende vraag erbij.

________________________________________________________________________________________________________________________________________________________________________________________________________________________________________________________________________________________________________________________________________________________________________________________________________________________________________________________________________________________________________________________________________________________________________________________________________________________________________________________________________________________________________________________________________________________________________________________________________________________________________________________________________________________________________________________________________________________________________________________________________________________________________________________________________________________________________________________________________________________________________________________________________________________________________________
