# Supplementary material for: The Cost-Effectiveness of Two Forms of Case Management Compared to a Control Group for Persons with Dementia and Their Informal Caregivers from a Societal Perspective
Source: PLoS One. 2016 Sep 21;11(9):e0160908. doi: 10.1371/journal.pone.0160908 (PMC5031395; doi:10.1371/journal.pone.0160908)
Supplement: S4 Case Record Form — Cost diary filled in by the informal caregiver (in Dutch). (DOC) [file pone.0160908.s006.doc]

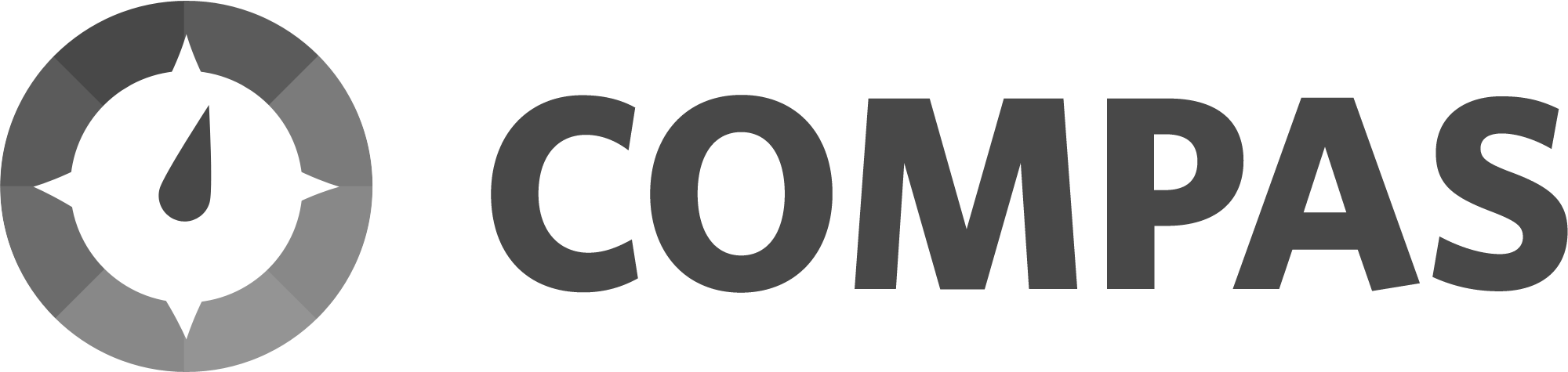


**Dagboek Zorggebruik**

**van uw naaste**

Periode: **__ - __ - 20__** t/m **__- __ - 20__** (half jaar)

# In te vullen door interviewer:

Studienummer: NL32949.029.10

Interviewernummer:

Zorgvrager nummer:

Geboortedatum mantelzorger:

# Introductie

Fijn dat u meedoet aan de COMPAS studie. In dit onderzoek willen we onder andere een indruk krijgen van de mate waarin mensen met geheugenproblemen gebruik maken van de gezondheidszorg. We willen u vragen om elk half jaar het zorggebruik van uw naaste in dit boekje bij te houden.

U krijgt dit boekje van de interviewer die bij u thuis komt. Elk half jaar neemt hij of zij het ingevulde boekje weer mee. U ontvangt dan weer een nieuw boekje voor het volgende half jaar.

In de boekjes kunt u bijhouden of uw naaste:

- de huisarts, therapeuten of alternatieve genezers heeft bezocht
- specialisten heeft bezocht
- is opgenomen in het ziekenhuis, al dan niet op de intensive care
- dagbehandeling heeft gehad (bijv. in een verpleeghuis of revalidatiecentrum)
- aanvullende onderzoeken (in het ziekenhuis) heeft gehad
- gespreksgroepen heeft gevolgd
- thuiszorg heeft ontvangen

Op de laatste pagina kunt u nog een toelichting geven op de vragen. Ook kunt u daar eventuele opmerkingen kwijt.

Wij adviseren om telkens als uw naaste naar de huisarts of een andere hulpverlener is geweest dit direct te noteren in dit boekje. Is uw naaste bijvoorbeeld naar de huisarts geweest vanwege griep, dan vult u dit in.

**Vragen?**

Indien u bij het invullen vragen heeft kunt u op werkdagen tijdens kantooruren contact opnemen met:

Eveline Spalburg, veldwerkcoördinator van de COMPAS studie

Haar telefoonnummer: 020 - 444 6327

U kunt ook e-mailen naar compas@vumc.nl

De interviewer zal aan het einde van het half jaar het boekje met u doornemen. Ook dan kunt u vragen stellen.

**Voorbeeld**

Hieronder volgt een voorbeeld van hoe het kostendagboekje ingevuld moet worden.

Uw naaste is in het afgelopen half jaar 2 keer bij de geriater in het ziekenhuis geweest. Uw naaste heeft 5 keer een bezoek gebracht aan de orthopeed.

U dient dit dan als volgt in te vullen:

**6. Specialist**

Heeft uw naaste contact gehad met een medisch specialist op de polikliniek in het ziekenhuis (zonder dat uw naaste was opgenomen in het ziekenhuis)?

□ ja □ nee

Bij welke specialist was dit?

|  | Aantal contacten |
| --- | --- |
| Geriater | **|** |
| Andere medisch specialist: | **||||** |

| **A. Huisarts, verpleegkundige van de huisarts en therapeuten** |
| --- |

**1. Huisarts**

Heeft uw naaste contact gehad met de huisarts of praktijkverpleegkundige?

□ ja □ nee

Indien ja: wat voor soort contact was dit?

|  | **Aantal contacten** |
| --- | --- |
| **Bezoek huisartsenpraktijk aan de huisarts** |  |
| **Telefonisch contact met de huisarts** |  |
| **Visite aan huis door de huisarts** |  |
| **Bezoek aan de huisartsenpost (contact met huisarts in de avonduren en in het weekend)** |  |
| **Bezoek huisartspraktijk aan de verpleegkundige van de huisarts** |  |
| **Telefonisch contact met de verpleegkundige van de huisarts** |  |
| **Visite aan huis door de verpleegkundige van de huisarts** |  |

**2. Specialist ouderengeneeskunde**

Heeft uw naaste thuis bezoek gehad van een specialist ouderengeneeskunde (verpleeghuisarts)?

□ ja □ nee

|  | **Aantal contacten** |
| --- | --- |
| **Indien ja: Hoeveel contacten met de specialist ouderengeneeskunde (verpleeghuisarts) zijn er geweest?** |  |

**3. Therapeut**

Heeft uw naaste contact gehad met een therapeut?

□ja □ nee

Indien ja: bij welke therapeut was dit?

|  | **Aantal contacten** |
| --- | --- |
| **Fysiotherapeut** |  |
| **Mensendieck/Cesar therapeut** |  |
| **Logopedist** |  |
| **Ergotherapeut** |  |
| **Manueel therapeut** |  |
| **Anders, namelijk:** |  |
| **Anders, namelijk:** |  |
| **Anders, namelijk:** |  |

**4. Alternatieve genezer**

Heeft uw naaste contact gehad met een alternatieve genezer?

□ ja □ nee

Indien ja: bij welke alternatieve genezer was dit?

|  | **Aantal contacten** |
| --- | --- |
| **Homeopaat** |  |
| **Acupuncturist** |  |
| **Natuurgenezer** |  |
| **Haptonoom** |  |
| **Chiropractor** |  |
| **Iriscopist** |  |
| **Anders, namelijk:** |  |
| **Anders, namelijk:** |  |

### 5. Psychische hulpverlening

Heeft uw naaste contact gehad met een hulpverlener voor psychische zorg?

□ ja □ nee

Indien ja: bij welke hulpverlener was dit?

|  | **Aantal contacten** |
| --- | --- |
| Psychiater in een ziekenhuis |  |
| Medewerker van GGZ instelling: |  |
| Sociaal Psychiatrisch Verpleegkundige (SPV’er) |  |
| Psycholoog |  |
| Arts |  |
| (ouderen) maatschappelijk werker |  |
| **Vrijgevestigde psychotherapeut** |  |
| **Vrijgevestigde psychiater** |  |
| **Consultatiebureau Alcohol & Drugs** |  |
| **Anders, namelijk:** |  |
| **Anders, namelijk:** |  |
| **Anders, namelijk:** |  |

| **B. Ziekenhuis en polikliniek** |
| --- |

**6. Specialist**

Heeft uw naaste contact gehad met een medisch specialist op de polikliniek in het ziekenhuis (zonder dat uw naaste was opgenomen in het ziekenhuis)?

□ ja □ nee

Indien ja: met welke medisch specialist was dit?

|  | **Aantal contacten** |
| --- | --- |
| Geriater |  |
| **Andere medisch specialist:** |  |

**7. Spoedeisende hulp**

Heeft uw naaste een bezoek gebracht aan de spoedeisende hulp van een ziekenhuis?

□ ja □ nee

|  | **Aantal bezoeken** |
| --- | --- |
| **Spoedeisende hulp** |  |

**8. Ziekenhuisopname**

Is uw naaste **langer dan 1 dag** opgenomen geweest in een ziekenhuis?

□ ja □nee

|  | **Aantal keren** |
| --- | --- |
| **Ziekenhuis opname** |  |

Namen ziekenhuizen en afdeling:

1..…………………………………………………………………….

2.………………………………………………………………………

3.………………………………………………………………………

|  | **Aantal dagen** | | |
| --- | --- | --- | --- |
| **Hoeveel dagen is uw naaste opgenomen geweest? (graag noteren per ziekenhuis)** | Ziekenhuis 1 | Ziekenhuis 2 | Ziekenhuis 3 |
| **Hoeveel dagen lag uw naaste hiervan op Intensive care? (graag noteren per ziekenhuis)** | Ziekenhuis 1 | Ziekenhuis 2 | Ziekenhuis 3 |
| **Was dit een geplande of ongeplande opname?** | □gepland  □ongepland | □gepland  □ongepland | □gepland  □ongepland |

**9. Dagopname ziekenhuis**

Is uw naaste **voor 1 dag** opgenomen geweest in een ziekenhuis?

□ ja □ nee

| **Ziekenhuis dagopname** | **Aantal keren** |
| --- | --- |
| **Naam ziekenhuis:** |  |

**10. Aanvullend onderzoek**

Heeft uw naaste aanvullende onderzoeken gehad?

□ ja □nee

Indien ja: welke aanvullende onderzoeken waren dit?

|  | **Aantal onderzoeken** |
| --- | --- |
| **Bloedonderzoek** |  |
| **Urineonderzoek** |  |
| **Röntgenfoto** |  |
| **Echoscopie** |  |
| **CT-scan** |  |
| **MRI-scan** |  |
| **Anders, namelijk:** |  |
| **Anders, namelijk:** |  |
| **Anders, namelijk:** |  |

| **C. Overige instellingen** |
| --- |

**11. Opnames**

Is uw naaste **langer dan 1 dag** opgenomen geweest in een andere instelling dan het ziekenhuis?

□ ja □ nee

Indien ja: waar is uw naaste opgenomen geweest?

|  | **Aantal dagen** | **Opname gepland/**  **ongepland** |
| --- | --- | --- |
| **Psychiatrisch ziekenhuis** |  | □gepland  □ongepland |
| **Revalidatiecentrum** |  | □gepland  □ongepland |
| **Verzorgingshuis** |  | □gepland  □ongepland |
| **Verpleeghuis** |  | □gepland  □ongepland |
| **Anders, namelijk:** |  | □gepland  □ongepland |
| **Anders, namelijk:** |  | □gepland  □ongepland |
| **Anders, namelijk:** |  | □gepland  □ongepland |

**12. Dagbehandeling/Dagopvang**

Heeft uw naaste dagbehandeling gehad of van dagopvang gebruik gemaakt in een andere instelling dan het ziekenhuis?

□ ja □ nee

Indien ja: waar was dit?

|  | **Aantal dagen** |
| --- | --- |
| **Psychiatrisch ziekenhuis** |  |
| **Revalidatiecentrum** |  |
| **Verzorgingshuis** |  |
| **Verpleeghuis** |  |
| **Anders, namelijk:** |  |
| **Anders, namelijk:** |  |
| **Anders, namelijk:** |  |

**13. Thuiszorg**

Heeft uw naaste thuiszorg gehad?

□ ja □ nee

Indien ja: welk soort thuiszorg was dit?

|  | **Aantal uren/week** | **Sinds wanneer**  **(datum)** |
| --- | --- | --- |
| **Huishoudelijke zorg van een medewerker van een thuiszorginstelling (voor het totale huishouden/gezin van uw naaste)** |  |  |
| **Huishoudelijke zorg via een (alpha)hulp die uw naaste zelf betaalt (uit het persoonsgebonden budget)** |  |  |
| **Persoonlijke verzorging (alleen voor uw naaste)**  **(*bijv. hulp bij eten, aankleden, douchen)*** |  |  |
| **Verpleging (alleen voor uw naaste )**  ***(bijv. wondverzorging, injecties)*** |  |  |

| **D. Gespreksgroepen en voorzieningen** |
| --- |

**14. Gespreksgroepen**

Heeft uw naaste deelgenomen aan een gespreksgroep?

□ ja □ nee

Indien ja: welke gespreksgroep was dit?

|  | **Aantal gesprekken** |
| --- | --- |
| **AA-groep (Anonieme Alcoholisten)** |  |
| **Gespreksgroep van de GGZ/het RIAGG** |  |
| **Anders, namelijk:** |  |
| **Anders, namelijk:** |  |
| **Anders, namelijk:** |  |

**15. Aanvullende voorzieningen**

Heeft uw naaste gebruik gemaakt van aanvullende voorzieningen?

□ ja □ nee

|  | **Aantal**  **keer/week** | **Sinds**  **wanneer**  **(datum)** |
| --- | --- | --- |
| **Maaltijdservice of tafeltje-dek-je** |  |  |
| **Voorzieningen voor vervoer**  **(bijv. regiotaxi)**  **naam van de voorziening:**  **……………………………………………** |  |  |
| **Bezoek aan ontmoetingscentrum** |  |  |
| **Bezoek aan Alzheimercafé** |  |  |
| **Anders, namelijk:** |  |  |
| **Anders, namelijk:** |  |  |

Indien ja: welke voorziening was dit?

Heeft u niet genoeg ruimte? Of heeft u informatie die u wilt aanvullen?

Op deze pagina kunt u de gegevens opschrijven die u niet in de tabellen kwijt kon. Schrijft u alstublieft het nummer van de betreffende vraag erbij.

_____________________________________________________________________________________________________________________________________________________________________________________________________________________________________________________________________________________________________________________________________________________________________________________________________________________________________________________________________________________________________________________________________________________________________________________________________________________________________________________________________________________________________________________________________________________________________________________________________________________________________________________________________________________________________________________________________________________________________________________________________________________________________________________________________________________________________________________________________________________________________________________________________________________________________________________________________________________________________
