# Supplementary figures and images for: The Cost-Effectiveness of Two Forms of Case Management Compared to a Control Group for Persons with Dementia and Their Informal Caregivers from a Societal Perspective
Source: PLoS One. 2016 Sep 21;11(9):e0160908. doi: 10.1371/journal.pone.0160908 (PMC5031395; doi:10.1371/journal.pone.0160908)

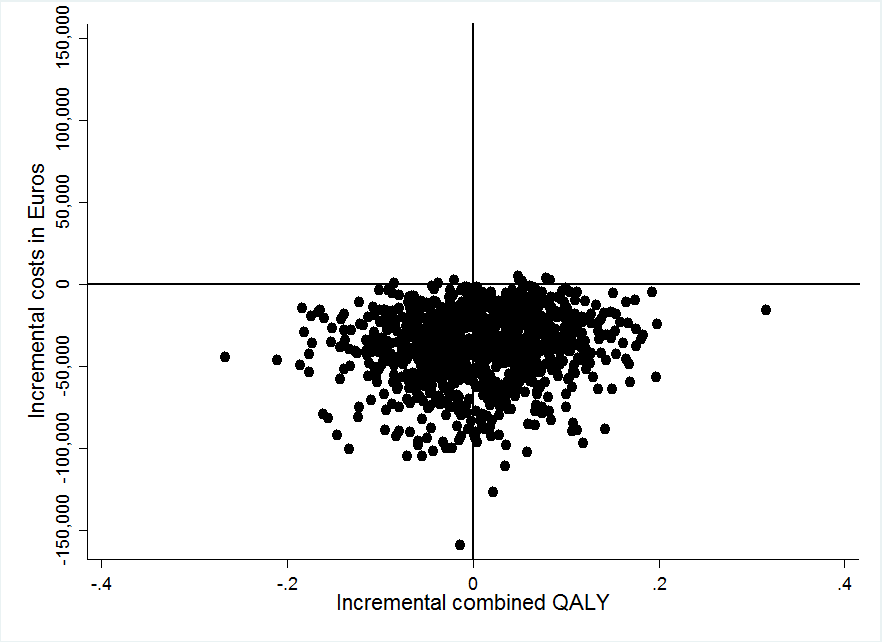

Supplement: S1 Fig — (TIF) [file pone.0160908.s014.tif]

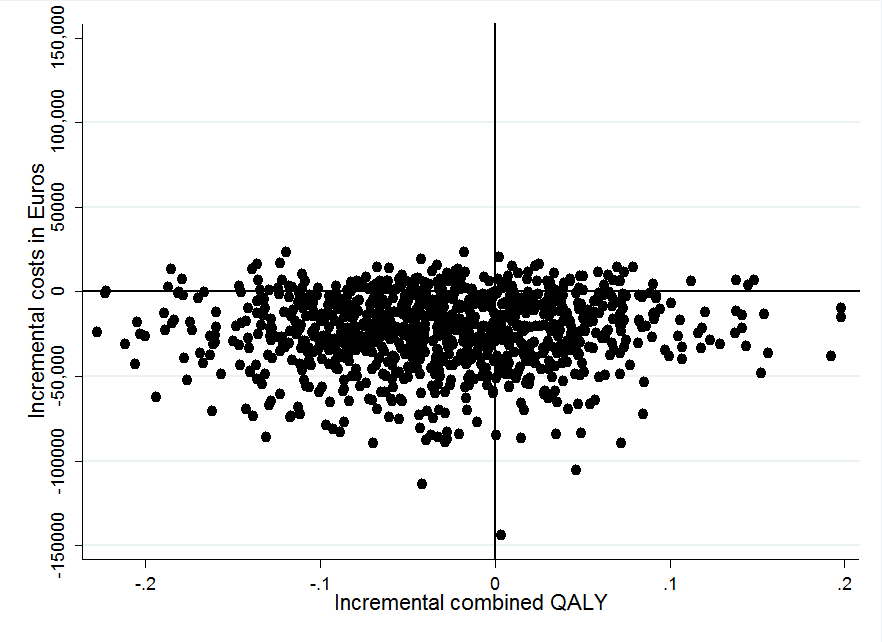

Supplement: S2 Fig — (TIF) [file pone.0160908.s015.tif]
