# Supplementary material for: The Cost-Effectiveness of Two Forms of Case Management Compared to a Control Group for Persons with Dementia and Their Informal Caregivers from a Societal Perspective
Source: PLoS One. 2016 Sep 21;11(9):e0160908. doi: 10.1371/journal.pone.0160908 (PMC5031395; doi:10.1371/journal.pone.0160908)
Supplement: S1 Table — Unit costs used for economic evaluation. All prices were adjusted for the year 2010 using consumer price index figures [26]. Healthcare utilization, and absenteeism from paid and unpaid work was valued using Dutch standard costs [27]. Costs of medications were valued using prices from the Royal Dutch Society for Pharmacy [28]. (DOCX) [file pone.0160908.s017.docx]

Supplementary Table 1 Unit costs – in the economic evaluation using consumer price index figures (in Euros)

| Cost category (2010) | Unit costs in euros |
| --- | --- |
| General practitioner, nurses and therapy |  |
| General practitioner |  |
| Visit to GP (per visit) | 28.35 |
| Telephone contact to GP | 14.17 |
| Visit from GP (per visit) to house | 43.53 |
| Visit to GP in the evening | 56.70 |
| visit to nurse practitioner | 9.02 |
| Telephone contact to nurse practitioner | 4.51 |
| Visit from nurse practitioner to house | 13.52 |
| Nursing home doctor | 44.56 |
| Therapy |  |
| Physiotherapy (per visit) | 37.90 |
| logotherapy | 33.41 |
| Ergotherapy (per visit) | 22.27 |
| Manual Therapy | 38.80 |
| Dietician | 27.33 |
| Alternative therapy |  |
| Homeopathy | 61.26 |
| Acupuncture | 45.38 |
| Haptonomy | 45.00 |
| chiropractor | 45.38 |
| Psychological care |  |
| Psychiatrist in a hospital (per visit) | 104.27 |
| Psychiatric Nurse | 65.80 |
| Psychologist (per visit) | 80.99 |
| Psychiatric doctor | 173.11 |
| Social worker | 65.80 |
| Freelance Psychologist | 77.95 |
| Freelance Psychiatrist | 104.27 |
| Hospital and polyclinic costs |  |
| Medical specialist | 72.89 |
| Geriatrician (per visit) | 72.89 |
| Other specialists (per visit) | 72.89 |
| Emergency help |  |
| ER visit | 152.86 |
| Hospital admissions |  |
| Hospital admission (per day) | 462.64 |
| ICU days | 2,209.93 |
| Day admission from hospital | 254.90 |
| other investigations |  |
| lab costs | 13.06 |
| x-rays | 50.36 |
| ultrasounds | 77.02 |
| CT scans | 194.85 |
| MRIs | 250.05 |
| Other settings |  |
| admissions |  |
| psychiatric hospital | 234.80 |
| rehabilitation care | 344.19 |
| elderly home | 91.11 |
| nursing home | 240.94 |
| Day Care | 91.11 |
| psychiatric hospital | 155.90 |
| rehabilitation care | 111.36 |
| elderly home | 54.67 |
| nursing home | 144.56 |
| Home care |  |
| self-paid | 24.30 |
| care only for the person with dementia | 44.54 |
| Nursing | 65.80 |
| meals on wheels per meal | 6.83 |
| taxi |  |
| Informal care (per hour) | 12.65 |
| case management costs |  |
| hourly wage of case managers in Linkage group | 40.80 |
| hourly wage of case managers in Linkage group | (40.8-46.35) |
|  |  |
