# Supplementary material for: The Cost-Effectiveness of Two Forms of Case Management Compared to a Control Group for Persons with Dementia and Their Informal Caregivers from a Societal Perspective
Source: PLoS One. 2016 Sep 21;11(9):e0160908. doi: 10.1371/journal.pone.0160908 (PMC5031395; doi:10.1371/journal.pone.0160908)
Supplement: S2 Table — (DOCX) [file pone.0160908.s018.docx]

| **Characteristics of different models** | **Linkage Model** | **Intensive Case management/ joint agency model** | **Care as usual** |
| --- | --- | --- | --- |
| **Central point for registration of cognitively impaired** | New clients are referred by GP or health specialist to the central registration point | New clients are referred by GP or health specialist to the Multidisciplinary team at central registration point | No |
| **Delivery of services** | Independent services and networks | Mainly offered by one organization | Various networks |
| **Possibility to diagnose dementia** | By GP or referral to e.g. memory clinic or elderly care physician of mental health care service | By GP or by Multidisciplinary team after referral to central registration point | By GP or referral to memory clinic or elderly care physician of mental health care service |
| **Case manager/ dementia nurse** | Yes | Yes | nurse present, incidentally |
| **Social psychiatric nurse** | No | No | Possible |
| **Multidisciplinary team** | External team that case managers can consult as per required | Case manager, elderly care physicians, neuropsychologist, neurologist, geriatrician, psychiatrist, dementia consultant all work within the same organisation as the case managers | No |
